# Quantum metrology with imperfect measurements

**DOI:** 10.1038/s41467-022-33563-8

**Published:** 2022-11-15

**Authors:** Yink Loong Len, Tuvia Gefen, Alex Retzker, Jan Kołodyński

**Affiliations:** 1 Centre for Quantum Optical Technologies, Centre of New Technologies, University of Warsaw, Banacha 2c, 02-097 Warszawa, Poland; 2 Institute for Quantum Information and Matter, Caltech, Pasadena, CA USA; 3 Racah Institute of Physics, The Hebrew University of Jerusalem, Jerusalem, 91904 Givat Ram Israel; 4 AWS Center for Quantum Computing, Pasadena, CA 91125 USA; 5 Present Address: Yale-NUS College, 16 College Avenue West, Singapore, 138527 Singapore

**Keywords:** Quantum metrology, Quantum information, Theoretical physics

## Abstract

The impact of measurement imperfections on quantum metrology protocols has not been approached in a systematic manner so far. In this work, we tackle this issue by generalising firstly the notion of quantum Fisher information to account for noisy detection, and propose tractable methods allowing for its approximate evaluation. We then show that in canonical scenarios involving *N* probes with local measurements undergoing readout noise, the optimal sensitivity depends crucially on the control operations allowed to counterbalance the measurement imperfections—with global control operations, the ideal sensitivity (e.g., the Heisenberg scaling) can always be recovered in the asymptotic *N* limit, while with local control operations the quantum-enhancement of sensitivity is constrained to a constant factor. We illustrate our findings with an example of NV-centre magnetometry, as well as schemes involving spin-1/2 probes with bit-flip errors affecting their two-outcome measurements, for which we find the input states and control unitary operations sufficient to attain the ultimate asymptotic precision.

## Abstract (web summary)

The effects of detection noise on quantum metrology performances have not been rigorously investigated yet. Here, the authors fill this gap by generalising the quantum Fisher information to the case of noisy readout, and showing the consequences the imperfect measurements bring.

## Introduction

One of the most promising quantum-enhanced technologies are the quantum sensors^1^ that by utilising quantum features of platforms such as solid-state spin systems^2,3^, atomic ensembles^4^, and interferometers^5^, or even gravitational-wave detectors^6^ are capable of operating at unprecedented sensitivities. They all rely on the architecture in which the parameter to be sensed (e.g., a magnetic or gravitational field) perturbs a well-isolated quantum system, which after being measured allows to precisely infer the perturbation and, hence, estimate well the parameter from the measurement data. In case the sensor consists of multiple probes (atoms, photons) their inter-entanglement opens doors to beating classical limits imposed on the estimation error^7^—a fact that ignited a series of breakthrough experiments^8–13^, being responsible also for the quantum-enhancement in gravitational-wave detection^6^.

These demonstrations are built upon various seminal theoretical works, in particular Refs. 14–16 that adopted parameter-inference problems to the quantum setting, and generalised the *Fisher information* (FI)^17^ to quantum systems. This general formalism provides tools to identify optimal probe states and measurements for any given quantum metrology task^18^. Interestingly it was shown that in many multi-probe scenarios, even those that involve entangled probes, optimal readout schemes turn out to be local—each of the probes can in principle be measured independently^18^.

In practice, however, engineering a measurement of a quantum system is a challenge per se—it relies on a scheme in which a meter component, typically light, interacts with the quantum sensor before being subsequently detected^19,20^. This allows the state of the probes to be separately controlled, at the price of the meter component carrying intrinsic noise that cannot be completely eradicated. As a result, the implemented measurement becomes *imperfect* with the measured data being noisy due to, e.g., finite resolution of the readout signal. Such an issue naturally arises across different sensing platforms: in nitrogen-vacancy (NV) centres in diamond^21–23^, superconducting-based quantum information processors^24–27^, trapped ions^28–31^, and interferometers involving photodetection^32,33^. Although for special detection-noise models (e.g., Gaussian blurring) the impact on quantum metrological performance and its compensation via the so-called interaction-based readout schemes has been studied^34–37^ and demonstrated^38^, a general analysis has been missing thus far.

Crucially, such a detection noise affecting the measurement cannot be generally put on the same grounds as the “standard” decoherence disturbing the (quantum) dynamics of the sensor before being measured^39^. In the latter case, the impact on quantum metrological performance has been thoroughly investigated^40–42^ and, moreover, shown under special conditions to be fully compensable by implementing methods of *quantum error correction*^43–47^. This contrasts the setting of readout noise that affects the classical output (outcomes) of a measurement, whose impact cannot be inverted by employing, e.g., the methods of *error mitigation*^48,49^ designed to recover statistical properties of the ideal readout data at the price of overhead, which cannot be simply ignored in the context of parameter estimation by increasing the sample size.

In our work, we formalize the problem of imperfect measurements in quantum metrology by firstly generalising the concept of *quantum Fisher information* (QFI)^16^ to the case of noisy readout. For pure probe-states, we explicitly relate the form of the resulting *imperfect QFI* to the perfect QFI, i.e., to the one applicable in presence of ideal detection. However, as we find the imperfect QFI not always to be directly computable, we discuss two general methods allowing one to tightly bound its value, as illustrated by a specific example of precision magnetometry performed with help of a NV centre^50,51^, for which the measurement imperfection is naturally inbuilt in the readout procedure^52,53^. Using the *conjugate-map decomposition* formalism, we also study when the measurement imperfections can be effectively interpreted as an extra source of “standard” decoherence, in order to show that this may occur only under very strict conditions.

Secondly, we focus on the canonical metrology schemes involving multiple probes^18^, in order investigate how do the measurement imperfections affect then the attainable sensitivity as a function of the probe number *N*, which in the ideal setting may scale at best quadratically with *N*—following the so-called ultimate *Heisenberg scaling* (HS)^7^. Considering general local measurements undergoing detection noise, we demonstrate that the achievable precision strongly depends on the type, i.e., global vs local, of control operations one is allowed to apply on the probes before the readout is performed.

In the former case, we prove a *go-theorem* which states that there always exists a *global* control unitary such that for pure states the imperfect QFI converges to the perfect QFI with *N*, and the detection noise can then be effectively ignored in the *N*→*∞* limit. We provide a recipe how to construct the required global unitary operation, and conjecture the general form of the optimal unitary from our numerical evidence. On the contrary, when restricted to *local* control unitaries, we resort to the concept of *quantum-classical channels*^54^ that describe then not only the evolution of each probe, but also the noisy measurement each probe is eventually subject to. For this complementary scenario, we establish a *no-go theorem* which states that whenever measurements exhibit any non-trivial local detection noise, attaining the HS becomes “elusive”^42^—the maximal quantum-enhancement becomes restricted to a constant factor with the estimation error asymptotically following at best a classical behaviour (∝1/*N*), which we refer to as the *standard scaling* (SS).

In order to illustrate the applicability of both theorems, we consider the phase-estimation example involving *N* spin-1/2 probes, whose binary measurements undergo bit-flip errors. On one hand, we explicitly construct the global unitary control operation, thanks to which the sensitivity quickly attains the HS with *N*, using for example the *GHZ state*^55^. On the other, when only local control operations are allowed, we evaluate the asymptotic SS-like bound on precision analytically, and prove its saturability with *N*→*∞* by considering the probes to be prepared in a *spin-squeezed state*^56,57^ and measuring effectively the mean value of their total angular momentum by adequately interpreting the noisy readout data. Furthermore, we apply the above analysis in Methods to the setting of optical interferometry involving *N*-photon states and imperfect detection, which suffers from both photonic losses and dark counts.

## Results

### Metrology with imperfect measurements

**Fig. 1:**
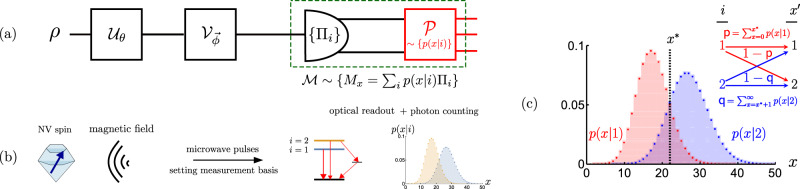
Imperfect measurements in quantum metrology. **a** Scheme of quantum metrology with an imperfect measurement. A quantum state *ρ* is fed into a unitary channel ${{{{{{{{\mathcal{U}}}}}}}}}_{\theta }$ which encodes the parameter of interest, *θ*. The probe is then rotated by a unitary ${{{{{{{{\mathcal{V}}}}}}}}}_{\vec{\phi}}$, so that a given projective measurement {Π_*i*_} can be performed in the preferred basis. The measurement ${{{{{{{\mathcal{M}}}}}}}}$ is, however, *imperfect*, i.e.,: different {*i*} outcomes are ‘inaccessible’ being mapped onto another set of ‘observable’ outcomes {*x*}, as specified by the *noisy detection channel* (stochastic map) ${{{{{{{\mathcal{P}}}}}}}} \sim \{p(x|i)\}$. **b** Phase sensing with the nitrogen-vacancy (NV) centre used as a spin probe. The spin of the NV is initialised in an equal superposition between the *m*_*s*_ = 0, 1 (*i* = 1, 2) energy-level states and evolves in presence of an external magnetic field, which induces a relative phase proportional to its strength. Microwave pulse is then applied to transform the relative phase into the population difference of the energy levels, which is then readout optically. The measurement procedure is inherently imperfect: the two populations indicating either *i* = 1 $\vee$ 2 each yield a (photon-number) signal that is stochastic and distributed according to a Poissonian profile, whose overlap renders the observed outcome *x* ambiguous. **c** Binary binning strategy or the threshold method**:** Infinite outcomes from Poissonian imperfections are categorised into two “*bins*” containing *x*≤x* and *x* > x*, respectively. As a result, the effective post-processing map ${{{{{{{\mathcal{P}}}}}}}}$ simplifies to an *asymmetric bit-flip channel* of the (projective) measurement outcomes summarised in the inset.

Let us consider a quantum metrology scenario depicted in Fig. 1(a), in which a *d*-dimensional qudit probe is prepared in a quantum state *ρ*, before it undergoes the dynamics encoding the parameter of interest *θ* that is represented by a unitary channel ${{{{{{{{\mathcal{U}}}}}}}}}_{\theta } \sim \, \{{U}_{\theta }\}$. Note that, here we focus on unitary encodings for clarity, but we formulate the theorems and lemmas in their most general form, often applicable beyond the unitary setting. The probe state thus transforms onto $\rho (\theta )={{{{{{{{\mathcal{U}}}}}}}}}_{\theta }[\rho ]={U}_{\theta }\rho {U}_{\theta }^{{{{\dagger}}} }$, and is subsequently rotated by a control unitary transformation ${{{{{{{{\mathcal{V}}}}}}}}}_{\vec{\phi }} \sim \, \{{V}_{\vec{\phi }}\}$ specified by the vector of parameters $\vec{\phi }$. It is then subjected to a fixed projective (von Neumann) measurement formally represented by a set of projection operators ${\{{\Pi }_{i}\}}_{i=1}^{d}$, i.e., Π_*i*_Π_*j*_ = *δ*_*i*,*j*_Π_*i*_ and $\mathop{\sum }\nolimits_{i=1}^{d}{\Pi }_{i}={{\mathbb{1}}}_{d}$. As a consequence, any projective measurement  with *d* outcomes can be implemented, where the purpose of the unitary operation ${{{{{{{{\mathcal{V}}}}}}}}}_{\vec{\phi }}$ is to select a particular measurement basis. In an *ideal* setting, every outcome *i* can be directly observed with its probability being given by the Born’s rule ${p}_{\theta,\vec{\phi }}(i)={{{{{{{\rm{Tr}}}}}}}}\{\rho (\theta ){\Pi }_{i,{\vec{\phi}}}\}$. Repeating the procedure over many rounds, an estimate $\tilde{\theta }$ can then be constructed based on all the collected data, which most accurately reproduces the true parameter value *θ*.

In particular, it is then natural to seek $\tilde{\theta }$ that minimises the *mean squared error* (MSE), ${\Delta }^{2}\tilde{\theta }$, while also minimising it over different measurement bases and initial states of the probe. For unbiased estimators, considering *ν* repetitions, the MSE is generally lower limited by the *quantum Cramér-Rao bound* (QCRB)^16,17^:

1 $$\nu {\Delta }^{2}\tilde{\theta }\ge \frac{1}{{{{{{{{\mathcal{F}}}}}}}}}\ge \frac{1}{\bar{{{{{{{{\mathcal{F}}}}}}}}}},$$

where ${{{{{{{\mathcal{F}}}}}}}}$ is the *quantum Fisher information* (QFI) that corresponds to the maximal (classical) *Fisher information* (FI), *F*, defined for a given distribution ${p}_{\theta,\vec{\phi }}$ and its derivative w.r.t. the estimated parameter, $\{{\dot{p}}_{\theta,\vec{\phi }}(i)\equiv {\partial }_{\theta }{p}_{\theta,\vec{\phi }}(i)\}$, i.e.,^16,17^:2that is optimised over all possible measurement bases $\vec{\phi }$. $\bar{{{{{{{{\mathcal{F}}}}}}}}}$ in Eq. (1) is the *channel QFI* which includes a further optimisation over all possible input probe states *ρ*, i.e., 

For perfect projective measurements this theory is well established—close analytical expressions for the QFI and the channel QFI exist. The QFI for any *ρ*(*θ*) reads^16^:

3 $${{{{{{{\mathcal{F}}}}}}}}[\rho (\theta )]={{{{{{{\rm{Tr}}}}}}}}\{\rho (\theta ){L}^{2}\},$$

where *L* is the symmetric-logarithmic derivative operator defined implicitly as ${\partial }_{\theta }\rho (\theta )=\frac{1}{2}(L\rho (\theta )+\rho (\theta )L)$, whose eigenbasis provides then the optimal measurement basis $\vec{\phi }$ that yields the QFI. Moreover, as the QFI is convex over quantum states^58^, its maximum is always achieved by pure input states $\psi= | \psi \rangle \langle \psi |$. Hence, for the unitary encoding $\psi (\theta )={{{{{{{{\mathcal{U}}}}}}}}}_{\theta }[\psi ]$ the channel QFI in Eq. (1) just reads^59^:

4 $$\bar{{{{{{{{\mathcal{F}}}}}}}}}[{{{{{{{{\mathcal{U}}}}}}}}}_{\theta }]={\left({\lambda }_{\max }({h}_{\theta })-{\lambda }_{\min }({h}_{\theta })\right)}^{2},$$

where ${h}_{\theta }=-{{{{{{{\rm{i}}}}}}}}({\partial }_{\theta }{U}_{\theta }){U}_{\theta }^{{{{\dagger}}} }$, ${\lambda }_{\max }({h}_{\theta })$ and ${\lambda }_{\min }({h}_{\theta })$ are the maximum and minimum eigenvalues of *h*_*θ*_, respectively, and $\bar{{{{{{{{\mathcal{F}}}}}}}}}$ is attained by *ψ*(*θ*) being an equal superposition of the corresponding two eigenvectors^18,59^.

In practical settings, however, perfect measurements are often beyond reach. Instead, one must deal with an *imperfect measurement* ${{{{{{{\mathcal{M}}}}}}}}$ that is formally described by a positive operator-valued measure (POVM)—a set consisting of ∣*X*∣ positive operators ${{{{{{{\mathcal{M}}}}}}}} \sim {\{{M}_{x}\}}_{x}$ that satisfy ${\sum }_{x}{M}_{x}={{\mathbb{1}}}_{d}$ and are now no longer projective. In Fig. 1(a) we present an important scenario common to many quantum-sensing platforms—e.g., NV-centre-based sensing depicted in Fig. 1(b). In particular, it includes a *noisy detection channel* ${{{{{{{\mathcal{P}}}}}}}}$ which distorts the ideal projective measurement ${\{{\Pi }_{i,\vec{\phi }}\}}_{i=1}^{d}$, so that its *d* outcomes become ‘inaccessible’, as they get randomised by some stochastic post-processing map ${{{{{{{\mathcal{P}}}}}}}} \sim \{p(x|i)\}$ into another set *X*∼{*x*} of ∣*X*∣ outcomes. The noise of the detection channel is then specified by the transition probability *p*(*x*∣*i*), which describes the probability of observing an outcome *x*, given that the projective measurement *i* was actually performed. In such a scenario any ‘observable’ outcome *x* occurs with probability ${q}_{\theta,\vec{\phi }}(x)=\mathop{\sum }\nolimits_{i=1}^{d}p(x|i)\,{p}_{\theta,\vec{\phi }}(i)={{{{{{{\rm{Tr}}}}}}}}\{\rho (\theta ){M}_{x,\vec{\phi }}\}$, where the corresponding imperfect measurement is then described by ${M}_{x,\vec{\phi }}=\mathop{\sum }\nolimits_{i=1}^{d}p(x|i)\,{\Pi }_{i,\vec{\phi }}$.

In presence of measurement imperfections, the QCRB (1) must be modified, so that it now contains instead the *imperfect QFI* and the *imperfect channel QFI*, which are then respectively defined as:5Once the assumption of perfect measurements is lifted, very little is known. In particular, although ${\bar{{{{{{{{\mathcal{F}}}}}}}}}}^{({{{{{{{\rm{im}}}}}}}})}$ can still be attained with some pure encoded state *ψ*(*θ*) by the convexity argument, there are no established general expression for ${{{{{{{{\mathcal{F}}}}}}}}}^{{{{{{{{\rm{(im)}}}}}}}}}$ and ${\bar{{{{{{{{\mathcal{F}}}}}}}}}}^{({{{{{{{\rm{im}}}}}}}})}$, as in Eqs. (3) and (4).

Firstly, we establish a formal relation between ${{{{{{{{\mathcal{F}}}}}}}}}^{{{{{{{{\rm{(im)}}}}}}}}}$ and ${{{{{{{\mathcal{F}}}}}}}}$ for all quantum metrology protocols involving pure states with arbitrary *θ*-encoding and imperfect measurements, which can be summarised as follows:

#### Lemma 1

(Quantum Fisher information with imperfect measurements). For any given pure encoded probe state, $\psi \left(\theta \right)$, and imperfect measurement, ${{{{{{{\mathcal{M}}}}}}}}$, the imperfect QFI reads

6 $${{{{{{{{\mathcal{F}}}}}}}}}^{{{{{{{{\rm{(im)}}}}}}}}}={\gamma }_{{{{{{{{\mathcal{M}}}}}}}}}\ {{{{{{{\mathcal{F}}}}}}}}[\psi (\theta )],$$

where

7 $${\gamma }_{{{{{{{{\mathcal{M}}}}}}}}}=\mathop{\max }\limits_{\left | \xi \right\rangle,\left | {\xi }_{\perp }\right\rangle }\mathop{\sum}\limits_{x}\frac{{{{{{{{\rm{Re}}}}}}}}{\left\{\left\langle {\xi }_{\perp }\right | {M}_{x}\left | \xi \right\rangle \right\}}^{2}}{\left\langle \xi \right | {M}_{x}\left | \xi \right\rangle }$$

is a constant $0\le {\gamma }_{{{{{{{{\mathcal{M}}}}}}}}}\le 1$ depending solely on the imperfect measurement, with the maximisation being performed over all pairs of orthogonal pure states $| \xi \rangle$ and $| {\xi }_{\perp }\rangle$.

We leave the explicit proof of Lemma 1 to the Supplementary Note A, but let us note that when assuming a unitary encoding, $\psi \left(\theta \right)={{{{{{{{\mathcal{U}}}}}}}}}_{\theta }[\psi ]$ and maximising Eq. (6) over all pure input states, *ψ*, it immediately follows that:

8 $${\bar{{{{{{{{\mathcal{F}}}}}}}}}}^{({{{{{{{\rm{im}}}}}}}})}={\gamma }_{{{{{{{{\mathcal{M}}}}}}}}}\bar{{{{{{{{\mathcal{F}}}}}}}}}[{{{{{{{{\mathcal{U}}}}}}}}}_{\theta }].$$

The constant ${\gamma }_{{{{{{{{\mathcal{M}}}}}}}}}$ specified in Eq. (7) has an intuitive meaning: it quantifies how well a particular known imperfect measurement ${{{{{{{\mathcal{M}}}}}}}}$ can distinguish at best a pair of orthogonal states. In fact, we prove explicitly in the Supplementary Note B that if there exist two orthogonal states that can be distinguished perfectly using ${{{{{{{\mathcal{M}}}}}}}}$, then ${\gamma }_{{{{{{{{\mathcal{M}}}}}}}}}=1$ and ${{{{{{{{\mathcal{F}}}}}}}}}^{{{{{{{{\rm{(im)}}}}}}}}}={{{{{{{\mathcal{F}}}}}}}}$.

Unfortunately, ${\gamma }_{{{{{{{{\mathcal{M}}}}}}}}}$ need not be easily computable, even numerically—consider, for instance, noisy detection channels ${{{{{{{\mathcal{P}}}}}}}}$ (e.g., the NV-centre example of Fig. 1 discussed below) that yield imperfect measurements with infinitely many outcomes *X* and, hence, the sum in Eq. (7) not even tractable. For this, we introduce in Methods two techniques that allow us to approximate well both ${{{{{{{{\mathcal{F}}}}}}}}}^{{{{{{{{\rm{(im)}}}}}}}}}$ and ${\bar{{{{{{{{\mathcal{F}}}}}}}}}}^{({{{{{{{\rm{im}}}}}}}})}$ in Eqs. (6) and (8), respectively, by considering tight lower bounds on the corresponding FIs. Note that Lemma 1 applies to any quantum metrology scheme involving pure states and imperfect measurements. Hence, it holds also when sensing, e.g., ‘critical’ parameters at phase transitions with noisy detection^60^.

### Example: Phase sensing with an NV centre

The utilisation of NV centres as quantum spin probes allows for precise magnetic-field sensing with unprecedented resolution^1^. For detailed account on sensors based on NV centres we refer the reader to Refs. 3, 61, 62; here, we focus on the very essence and briefly outline the canonical NV-centre-based sensing protocol based on a Ramsey-type sequence of pulses, schematically depicted in Fig. 1(b), and as described in the Methods section.

In short, the sensing of a magnetic field with an NV centre fits into the general formalism introduced above, whereby now the encoding channel is ${{{{{{{{\mathcal{U}}}}}}}}}_{\theta } \sim \{{U}_{\theta }={{{{{{{{\rm{e}}}}}}}}}^{{{{{{{{\rm{i}}}}}}}}h\theta }\}$, $h={\sigma }_{{{{{{{{\rm{z}}}}}}}}}/2=(\left|0\right\rangle \left\langle 0\right | -\left | 1\right\rangle \left\langle 1\right | )/2$, with ${\Pi }_{1}=\left|0\right\rangle \left\langle 0\right|$ and ${\Pi }_{2}=\left|1\right\rangle \left\langle 1\right|$, which can be rotated into another measurement basis by a Ramsey pulse. These projective measurements are, however, not ideally implemented, as the fluoresence readout technique is inherently noisy. The final ‘observed’ outcomes are the number of collected photons *X* = {0, 1, 2,...}, distributed according to the two Poissonian distributions $p(x|1)={{{{{{{{\rm{e}}}}}}}}}^{-{\lambda }_{\left|0\right\rangle }}{({\lambda }_{\left|0\right\rangle })}^{x}/x!$ and $p(x|2)={{{{{{{{\rm{e}}}}}}}}}^{-{\lambda }_{\left|1\right\rangle }}{({\lambda }_{\left|1\right\rangle })}^{x}/x$, whose means, ${\lambda }_{\left|0\right\rangle }$ and ${\lambda }_{\left|1\right\rangle }$, differ depending on which energy state the NV spin was previously projected onto by ${\Pi }_{1,\vec{\phi }}$ or ${\Pi }_{2,\vec{\phi }}$.

**Fig. 2:**
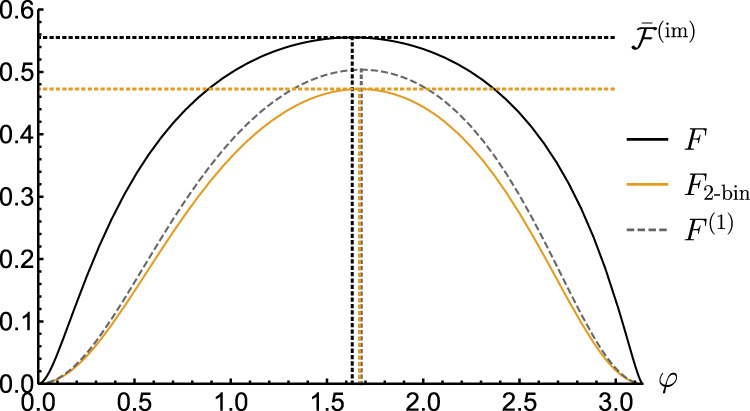
Computing FI for sensing phase *θ* with measurements experiencing Poissonian noise. The FIs are presented as a function of the relative measurement-basis angle, *φ* = *θ* + *ϕ*, whose changes are equivalent to varying the input-state angle, *ϕ*, and/or the estimated phase, *θ* (we set ${\lambda }_{\left|1\right\rangle }/{\lambda }_{\left|0\right\rangle }=0.65$ and ${\lambda }_{\left|0\right\rangle }=27$^76^). The exact *F* (solid black) is numerically approximated by summing over *x*≤100 in Eq. (9), while *F*_2-bin_ (solid orange) accounts for the binning method in Eq. (11) with the choice of the binning boundary x* further optimised over. We also plot *F*^(1)^ (dashed grey), the FI approximated with using just the first two moments of the observed probability distribution. The vertical dotted lines indicate the (optimal) *φ* at each of the respective quantities is maximised. Note that when the measurement is perfect, $\bar{{{{{{{{\mathcal{F}}}}}}}}}[{{{{{{{{\mathcal{U}}}}}}}}}_{\theta }]$ is unity, and is for all choices of the angle *φ* (not shown). The horizontal dotted lines depict the (numerically approximated) values of Eq. (8), ${\bar{{{{{{{{\mathcal{F}}}}}}}}}}^{({{{{{{{\rm{im}}}}}}}})}={\gamma }_{{{{{{{{\mathcal{M}}}}}}}}}$, for respective cases of the exact *F* and its two-binned version.

In order to determine ${\bar{{{{{{{{\mathcal{F}}}}}}}}}}^{({{{{{{{\rm{im}}}}}}}})}$ in this case, we first note that only pure input states and projective measurements, whose elements lie in the equatorial plane in the Bloch-ball representation need to be considered (see Supplementary Note C for the proof). Hence, after fixing the measurement to ${\Pi }_{1(2),\vec{\phi }}=\left|\pm \right\rangle \left\langle \pm \right|,\left|\pm \right\rangle=(\left|0\right\rangle \pm \left|1\right\rangle )/\sqrt{2}$, the maximisation in Eq. (2) simplifies to optimising over a single parameter *ϕ* of the input state $\left|\psi \right\rangle=(\left|0\right\rangle+{{{{{{{\rm{i}}}}}}}}{{{{{{{{\rm{e}}}}}}}}}^{-{{{{{{{\rm{i}}}}}}}}\phi }\left|1\right\rangle )/\sqrt{2}$, so that ${\bar{{{{{{{{\mathcal{F}}}}}}}}}}^{({{{{{{{\rm{im}}}}}}}})}=\mathop{\max }\limits_{\varphi }F$ with

9 $$F=\mathop{\sum}\limits_{x}\frac{\frac{1}{2}{\left(p(x|1)-p(x|2)\right)}^{2}{\cos }^{2}\varphi }{p(x|1)+p(x|2)+\left(p(x|1)-p(x|2)\right)\sin \varphi },$$

and *φ* ≔ *θ* + *ϕ*. As neither *F* nor ${\bar{{{{{{{{\mathcal{F}}}}}}}}}}^{({{{{{{{\rm{im}}}}}}}})}$ can be evaluated analytically due to the infinite summation in Eq. (9), their values may only be approximated numerically by considering a sufficient cut-off—as done in Fig. 2 (see the solid and dashed black lines).

A systematic and practically motivated approach allowing to lower-bound well *F* and ${\bar{{{{{{{{\mathcal{F}}}}}}}}}}^{({{{{{{{\rm{im}}}}}}}})}$ corresponds to grouping the infinite outcomes *X* into a finite number of categories: “bins”. Although complex “binning” strategies are possible (see Methods), the crudest one considers just two bins (2-bin)—an approach known as the “threshold method” in the context of NV-readout^52,53^. The binary outcome ${X}^{\prime}$ is then formed by interpreting all the photon-counts from *x* = 0 up to a certain x* as ${x}^{\prime}=1$, while the rest as ${x}^{\prime}=2$. This results in an effective *asymmetric bit-flip channel*^63^, ${{{{{{{\mathcal{P}}}}}}}}$, mapping the ideal outcomes *I* onto ${X}^{\prime}$, which we depict in Fig. 1(c) for the case of photon-counts following Poissonian distributions, upon defining ${\mathsf{p}}$ ≔  and , as well as *η* ≔ ${\mathsf{p}}+{\mathsf{q-1}}$ and *δ* ≔ ${\mathsf{p}} - {\mathsf{q}}$.

As a result, we can analytically compute for the 2-bin strategy both the corresponding imperfect QFI and the imperfect channel QFI as, respectively:

10 $${F}_{{{{{{{{\rm{2-bin}}}}}}}}}^{*}=\frac{{\eta }^{2}{\cos }^{2}\varphi }{1-{(\delta+\eta \sin \varphi )}^{2}},$$

11 $${\bar{F}}_{{{{{{{{\rm{2-bin}}}}}}}}}^{*}	=\mathop{\max }\limits_{\varphi }{F}_{{{{{{{{\rm{2-bin}}}}}}}}}^{*}=\eta (\eta+\delta \sin {\varphi }_{{{{{{{{\rm{opt}}}}}}}}})\\ 	=1-{\left(\sqrt{{\mathsf{p}}\left(1-{\mathsf{q}}\right)}+\sqrt{{\mathsf{q}}\left(1-{\mathsf{p}}\right)}\right)}^{2},$$

where the optimal angle parametrising the input state reads *ϕ*_opt_ = *φ*_opt_ − *θ*, with ${\varphi }_{{{{{{{{\rm{opt}}}}}}}}}={\sin }^{-1}(\Theta )$ and

12 $$\Theta=\frac{1-{\delta }^{2}-{\eta }^{2}-\sqrt{{(1-{\delta }^{2}-{\eta }^{2})}^{2}-4{\delta }^{2}{\eta }^{2}}}{2\delta \eta }.$$

In Fig. 2 we plot *F*_2-bin_ that corresponds to ${F}_{{{{{{{{\rm{2-bin}}}}}}}}}^{*}$ being further maximised over the binning boundary x*—it allows us to verify that *ϕ*_opt_ provides indeed a very good approximation of the optimal input state.

We close the analysis of imperfect measurements in the single-probe scenario by briefly discussing another general method to approximate *F* and ${\bar{{{{{{{{\mathcal{F}}}}}}}}}}^{({{{{{{{\rm{im}}}}}}}})}$. It relies on a construction (see Methods for the full methodology) of a convergent hierarchy of lower bounds on the FI, *F*^(*k*)^≤*F*, which are obtained by considering subsequent 2*k* moments of the probability distribution ${q}_{\theta,\vec{\phi }}$ describing the set of ‘observed’ outcomes *X*, even if infinite^64^. In Fig. 2, we present *F*^(1)^ based on only first two moments of ${q}_{\theta,\vec{\phi }}$, which, however, contain most information about the estimated phase *θ*, so that the method also predicts the optimal input state very well.

### Relations to quantum metrology with noisy encoding

**Fig. 3:**
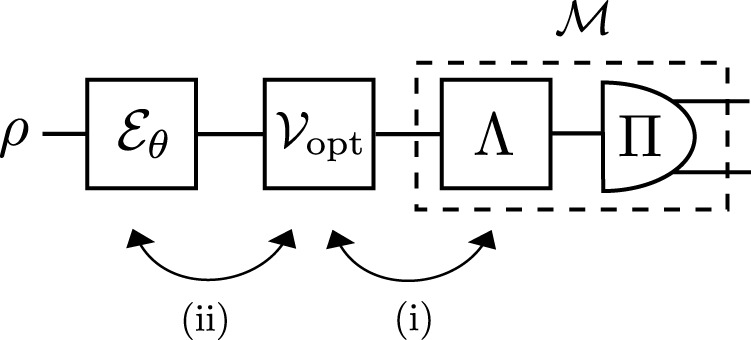
Conjugate-map decomposition of an imperfect measurement. The scheme of Fig. 1(a) with the imperfect measurement ${{{{{{{\mathcal{M}}}}}}}}$ decomposed according to its conjugate-map decomposition, i.e., ${{{{{{{\mathcal{M}}}}}}}}={\Lambda }^{{{{\dagger}}} }[\Pi ]$ where Λ is a quantum channel such that {Π_*x*_} forms a projective measurement in the output space of ∣*X*∣ ‘observable’ outcomes. By ${{{{{{{{\mathcal{V}}}}}}}}}_{{{{{{{{\rm{opt}}}}}}}}}$ we denote the optimal unitary control required by the imperfect (channel) QFI ${{{{{{{{\mathcal{F}}}}}}}}}^{{{{{{{{\rm{(im)}}}}}}}}}$ (${\bar{{{{{{{{\mathcal{F}}}}}}}}}}^{({{{{{{{\rm{im}}}}}}}})}$), which in principle depends on the particular form of the input state *ρ*, encoding ${{{{{{{{\mathcal{E}}}}}}}}}_{\theta }$ and the measurement ${{{{{{{\mathcal{M}}}}}}}}$. Still, if (i): ${{{{{{{{\mathcal{V}}}}}}}}}_{{{{{{{{\rm{opt}}}}}}}}}$ commutes with the map Λ, then ${{{{{{{{\mathcal{F}}}}}}}}}^{{{{{{{{\rm{(im)}}}}}}}}}\le {{{{{{{\mathcal{F}}}}}}}}[\Lambda \circ {{{{{{{{\mathcal{E}}}}}}}}}_{\theta }[\rho ]]$ and, hence, ${\bar{{{{{{{{\mathcal{F}}}}}}}}}}^{({{{{{{{\rm{im}}}}}}}})}\le \bar{{{{{{{{\mathcal{F}}}}}}}}}[\Lambda \circ {{{{{{{{\mathcal{E}}}}}}}}}_{\theta }]$. However, the latter is also true if (ii): ${{{{{{{{\mathcal{V}}}}}}}}}_{{{{{{{{\rm{opt}}}}}}}}}$ commutes with the encoding ${{{{{{{{\mathcal{E}}}}}}}}}_{\theta }$.

Any imperfect measurement ${{{{{{{\mathcal{M}}}}}}}}$ admits a *conjugate-map decomposition*, ${{{{{{{\mathcal{M}}}}}}}}={\Lambda }^{{{{\dagger}}} }[\Pi ]$, i.e., all its elements can be expressed as *M*_*x*_ = Λ^†^[Π_*x*_], where $\Pi \sim {\{{\Pi }_{x}\}}_{x=1}^{|X|}$ form a projective measurement in ${{{{{{{{\mathcal{H}}}}}}}}}_{|X|}$ and $\Lambda :\,{{{{{{{\mathcal{B}}}}}}}}({{{{{{{{\mathcal{H}}}}}}}}}_{d})\to {{{{{{{\mathcal{B}}}}}}}}({{{{{{{{\mathcal{H}}}}}}}}}_{|X|})$ is a quantum channel that may always be constructed (see Supplementary Note D, and Supplementary Figure 1), where ${{{{{{{\mathcal{B}}}}}}}}({{{{{{{{\mathcal{H}}}}}}}}}_{\ell })$ denotes the set of bounded linear operators on the Hilbert space ${{{{{{{{\mathcal{H}}}}}}}}}_{\ell }$ of dimension *ℓ*. Hence, given the channel Λ, for any two operators $A\in {{{{{{{\mathcal{B}}}}}}}}({{{{{{{{\mathcal{H}}}}}}}}}_{d})$, $B\in {{{{{{{\mathcal{B}}}}}}}}({{{{{{{{\mathcal{H}}}}}}}}}_{|X|})$, Λ^†^ is defined by ${{{{{{{\rm{Tr}}}}}}}}\{{\Lambda }^{{{{\dagger}}} }[B]A\}\equiv {{{{{{{\rm{Tr}}}}}}}}\{B\Lambda [A]\}$. This implies that any imperfect measurement ${{{{{{{\mathcal{M}}}}}}}}$ can always be represented by the action of a fictitious channel Λ, followed by a projective (“ideal”) measurement {Π_*x*_} that acts in the space of the ‘observed’ outcomes—compare Fig. 3 with Fig. 1(a).

However, the channel Λ acts after both the (here, arbitrary) parameter encoding ${{{{{{{{\mathcal{E}}}}}}}}}_{\theta }$ and the optimal unitary control denoted by ${{{{{{{{\mathcal{V}}}}}}}}}_{{{{{{{{\rm{opt}}}}}}}}}$ in Fig. 3, of which the latter generally depends on a particular form of all: the input state *ρ*, the encoding ${{{{{{{{\mathcal{E}}}}}}}}}_{\theta }$, and the imperfect measurement ${{{{{{{\mathcal{M}}}}}}}}$. Only in the very special case when one can find Λ such that it commutes with ${{{{{{{{\mathcal{V}}}}}}}}}_{{{{{{{{\rm{opt}}}}}}}}}$—case (i) in Fig. 3—the problem can be interpreted as an instance of the “standard” noisy metrology scenario^39^. It is so, as the corresponding imperfect QFI must then obey ${{{{{{{{\mathcal{F}}}}}}}}}^{{{{{{{{\rm{(im)}}}}}}}}}\le {{{{{{{\mathcal{F}}}}}}}}[\Lambda \circ {{{{{{{{\mathcal{E}}}}}}}}}_{\theta }[\rho ]]$, which upon maximisation over input states implies also ${\bar{{{{{{{{\mathcal{F}}}}}}}}}}^{({{{{{{{\rm{im}}}}}}}})}\le \bar{{{{{{{{\mathcal{F}}}}}}}}}[\Lambda \circ {{{{{{{{\mathcal{E}}}}}}}}}_{\theta }]$ for the imperfect channel QFI. The latter inequality may be independently assured if the optimal control ${{{{{{{{\mathcal{V}}}}}}}}}_{{{{{{{{\rm{opt}}}}}}}}}$ commutes with the parameter encoding ${{{{{{{{\mathcal{E}}}}}}}}}_{\theta }$ instead—case (ii) in Fig. 3—as this allows ${{{{{{{{\mathcal{V}}}}}}}}}_{{{{{{{{\rm{opt}}}}}}}}}$ to be incorporated into the maximisation over *ρ*.

Although we demonstrate that both above bounds can be computed via a semi-definite programme via a ‘seesaw’ method (see Supplementary Note D and Supplementary Figure 4), which may also incorporate optimisation over all valid conjugate-map decompositions of ${{{{{{{\mathcal{M}}}}}}}}$, their applicability is very limited. In particular, their validity can only be a priori verified if the problem exhibits some symmetry—the *G*-covariance that we discuss in Methods—that must ensure the commutativity (i) or (ii) of the optimal control ${{{{{{{{\mathcal{V}}}}}}}}}_{{{{{{{{\rm{opt}}}}}}}}}$ in Fig. 3, without knowing its actual form.

Even in the simple qubit case with unitary encoding, ${U}_{\theta }={{{{{{{{\rm{e}}}}}}}}}^{{{{{{{{\rm{i}}}}}}}}\frac{{\sigma }_{{{{{{{{\rm{z}}}}}}}}}}{2}\theta }$, and the binary outcome of a projective measurement being randomly flipped—equivalent to the NV-motivated scenario with 2-binning that yields the imperfect channel QFI (11)—Λ must be *phase-covariant*^65^ for ${{{{{{{{\mathcal{F}}}}}}}}}^{{{{{{{{\rm{(im)}}}}}}}}}\le {{{{{{{\mathcal{F}}}}}}}}[\Lambda \circ {{{{{{{{\mathcal{U}}}}}}}}}_{\theta }[\rho ]]$ to hold (see Supplementary Note D). While this may be satisfied only if there exists some *ϕ*∈(0, 2*π*) such that $\frac{4{\eta }^{2}}{{\sin }^{2}\phi }+\frac{{\delta }^{2}}{{\cos }^{2}\phi }\le 1$, the resulting bound is tight only for symmetric bit-flips (*δ* = 0); see Supplementary Figure 2 for graphical illustration. Furthermore, considering already a two-qubit system with local imperfect measurements of this type, ${{{{{{{{\mathcal{V}}}}}}}}}_{{{{{{{{\rm{opt}}}}}}}}}$ ceases to commute with the encoding, ${U}_{\theta }^{\otimes 2}$, so that even ${\bar{{{{{{{{\mathcal{F}}}}}}}}}}^{({{{{{{{\rm{im}}}}}}}})}\le \bar{{{{{{{{\mathcal{F}}}}}}}}}[{(\Lambda \circ {{{{{{{{\mathcal{U}}}}}}}}}_{\theta })}^{\otimes 2}]$ cannot be assured (see Supplementary Note D and Supplementary Figure 3). This opens doors to circumvent the no-go theorems of quantum metrology with uncorrelated noise^41,42^, as exploited in the multi-probe schemes discussed below.

### Multi-probe scenarios

**Fig. 4:**
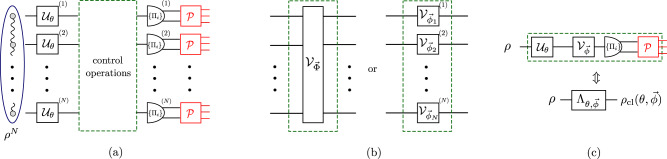
Multi-probe scenarios supplemented by control operations. **a** Canonical multi-probe scenario of quantum metrology with imperfect measurements. *N* probes, generally prepared in a entangled state, *ρ*^*N*^, undergo identical parameter encoding, ${{{{{{{{\mathcal{U}}}}}}}}}_{\theta }$, and are subject to identical local projective measurements {Π_*i*_}, whose outcomes are affected by the noisy detection channel (stochastic map) ${{{{{{{\mathcal{P}}}}}}}}$. In between the encoding and measurement, control operations are applied and optimised in order to compensate for local measurement imperfections, so that the minimal error in estimating *θ* can be attained. **b** Control operations in **a** may always be represented by a *global* unitary transformation, ${{{{{{{{\mathcal{V}}}}}}}}}_{\vec{\Phi }}$; or be rather constrained to a product of general *local* unitaries, ${\bigotimes }_{j=1}^{N}{{{{{{{{\mathcal{V}}}}}}}}}_{{\vec{\phi }}_{j}}^{(j)}$. **c** Single-probe evolution as a *quantum-classical channel*, denoted as ${\Lambda }_{\theta,\vec{\phi }}$ that transforms the *d*-dimensional state *ρ* of the probe into a classical state ${\rho }_{{{{{{{{\rm{cl}}}}}}}}}(\theta,\vec{\phi })$ defined in a fictitious Hilbert space, whose dimension is specified by the number of outcomes of the noisy detection channel ${{{{{{{\mathcal{P}}}}}}}}$.

We turn now our focus to *multi-probe* scenarios of quantum metrology, in particular, the canonical one in which the parameter is encoded locally onto each probe, so that the inter-probe entanglement can prove its crucial usefulness, e.g., to reach the HS of precision, whereas the ideal projective measurement can be considered to be *local* without loss of generality^18^. While including imperfect measurements into the picture, we depict such a scheme in Fig. 4(a), in which *N* qudits are prepared in a (possibly entangled) state *ρ*^*N*^ before undergoing a unitary transformation ${{{{{{{{\mathcal{U}}}}}}}}}_{\theta }^{N} \sim \{{U}_{\theta }^{N}\}$, so that ${\rho }^{N}(\theta )={U}_{\theta }^{N}{\rho }^{N}{U}_{\theta }^{N{{{\dagger}}} }$, where ${U}_{\theta }^{N}={U}_{\theta }^{\otimes N}$ in the canonical scenario^18^. Each probe is still measured independently but in an imperfect manner, so that the overall POVM corresponds now to ${{{{{{{{\mathcal{M}}}}}}}}}^{\otimes N} \sim \{{M}_{{{{{{{{\boldsymbol{x}}}}}}}}}\}=\{{M}_{{x}_{1}}\otimes {M}_{{x}_{2}}\otimes \cdots \otimes {M}_{{x}_{N}}\}$. For instance, as shown in Fig. 4(a), each {*M*_*x*_} may be obtained by randomising outcomes of projectors {Π_*i*_} according to some stochastic map ${{{{{{{\mathcal{P}}}}}}}} \sim \{p(x|i)\}$ representing the noisy detection channel.

In order to compensate for measurement imperfections, we allow for control operations to be performed on all the probes before being measured. However, we differentiate between the two extreme situations, see Fig. 4(b), in which the control operations can act collectively on all the probes—being represented by a *global* unitary channel ${{{{{{{{\mathcal{V}}}}}}}}}_{\vec{\Phi }} \sim \{{V}_{\vec{\Phi }}\}$ specified by the vector of parameters $\vec{\Phi }$; or can affect them only locally—corresponding to a product of (possibly non-identical) *local* unitary channels ${\bigotimes }_{\ell=1}^{N}{{{{{{{{\mathcal{V}}}}}}}}}_{{\vec{\phi }}_{\ell }}^{(\ell )}$ with ${{{{{{{{\mathcal{V}}}}}}}}}_{{\vec{\phi }}_{\ell }} \sim \{{V}_{{\vec{\phi }}_{\ell }}\}$, each of which is specified by a separate vector of parameters ${\vec{\phi }}_{\ell }$.

As in the general case, the QCRB (1) determines then the ultimate attainable sensitivity. In particular, given a large number *ν* of protocol repetitions, the MSE ${\Delta }^{2}{\tilde{\theta }}_{N}$, which now depends on the number of probes *N* employed in each protocol round, is ultimately dictated by the lower bounds:

13 $$\nu {\Delta }^{2}{\tilde{\theta }}_{N}\ge \frac{1}{{{{{{{{{\mathcal{F}}}}}}}}}_{N}^{{{{{{{{\rm{(im)}}}}}}}}}}\ge \frac{1}{{\bar{{{{{{{{\mathcal{F}}}}}}}}}}_{N}^{({{{{{{{\rm{im}}}}}}}})}},$$

where14are again the imperfect QFI and the imperfect channel QFI, respectively, see Eq. (5), but evaluated now for the case of *N* probes. Similarly, *F*_*N*_ is the *N*-probe version of Eq. (2), whose maximisation over all local measurement settings becomes now incorporated into the optimisation over control operations, either global $\vec{\Phi }$ or local ${\{{\vec{\phi }}_{\ell }\}}_{\ell }$.

### Global control operations

We first consider multi-probe scenarios in which one is allowed to perform global unitary control operations, ${{{{{{{{\mathcal{V}}}}}}}}}_{\vec{\Phi }}$ in Fig. 4(b), to compensate for measurement imperfections. In such a case, let us term an imperfect measurement ${{{{{{{\mathcal{M}}}}}}}}$ *information-erasing*, if all its elements *M*_*x*_ are proportional to identity, so that no information can be extracted. Then, it follows from Lemma 1 that:

#### Theorem 1

(Multi-probe metrology scheme with global control). For any pure encoded *N*-probe state ${\psi }^{N}(\theta )=\left|{\psi }^{N}\left(\theta \right)\right\rangle \left\langle {\psi }^{N}\left(\theta \right)\right|$, and any imperfect measurement ${{{{{{{{\mathcal{M}}}}}}}}}^{\otimes N}$ that is not information-erasing and operates independently on each of the probes, the imperfect QFI converges to the perfect QFI for large enough *N*:

15 $${{{{{{{{\mathcal{F}}}}}}}}}_{N}^{{{{{{{{\rm{(im)}}}}}}}}}\mathop{=}\limits_{N\to \infty }{{{{{{{\mathcal{F}}}}}}}}[{\psi }^{N}(\theta )].$$

We differ the proof to the Supplementary Note E, where we explicitly show that for any non–information-erasing imperfect measurement ${{{{{{{\mathcal{M}}}}}}}}$, the resulting constant factor ${\gamma }_{{{{{{{{{\mathcal{M}}}}}}}}}^{\otimes N}}$ appearing in Lemma 1—which now depends and must monotonically grow with the probe number *N*—satisfies ${\gamma }_{{{{{{{{{\mathcal{M}}}}}}}}}^{\otimes N}}\to 1$ as ${N\to \infty }$, as generally ${\gamma }_{{{{{{{{\mathcal{M}}}}}}}}}\le {\gamma }_{{{{{{{{\mathcal{M}}}}}}}}\otimes I}\le {\gamma }_{{{{{{{{\mathcal{M}}}}}}}}\otimes {{{{{{{\mathcal{M}}}}}}}}}$; see the Supplementary Note B for the proof. Intuitively, recall that ${\gamma }_{{{{{{{{{\mathcal{M}}}}}}}}}^{\otimes N}}$ quantifies how well one can distinguish at best some two orthogonal states $|{\xi }^{N}\rangle$ and $|{\xi }_{\perp }^{N}\rangle$. Hence, we can always consider $|{\xi }^{N}\rangle={|\xi \rangle }^{\otimes N}$ and $|{\xi }_{\perp }^{N}\rangle={|{\xi }_{\perp }\rangle }^{\otimes N}$, whose effective “overlap” for the resulting imperfect measurement ${{{{{{{{\mathcal{M}}}}}}}}}^{\otimes N} \sim \, \{{M}_{{{{{{{{\boldsymbol{x}}}}}}}}}\}$ reads

16 $$\mathop{\sum}\limits_{{{{{{{{\boldsymbol{x}}}}}}}}}\sqrt{\langle {\xi }^{N}|{M}_{{{{{{{{\boldsymbol{x}}}}}}}}}|{\xi }^{N}\rangle \langle {\xi }_{\perp }^{N}|{M}_{{{{{{{{\boldsymbol{x}}}}}}}}}|{\xi }_{\perp }^{N}\rangle }={c}^{N}$$

with $c={\sum }_{x}\sqrt{\langle \xi |{M}_{x}|\xi \rangle \langle {\xi }_{\perp }|{M}_{x}|{\xi }_{\perp }\rangle } < 1$, and is thus assured to be exponentially decaying to zero with *N*. This implies perfect distinguishability and, hence, attaining perfect QFI as ${N\to \infty }$, with the convergence rate depending solely on the single-probe POVM, ${{{{{{{\mathcal{M}}}}}}}} \sim \, \{{M}_{x}\}$.

More formally, we establish the existence of a global unitary ${V}_{\vec{\Phi }}$, such that the following lower bound holds:17where 0 ≤ *c* ≤ 1 depends only on ${{{{{{{\mathcal{M}}}}}}}}$ and the unitary ${V}_{\vec{\Phi }}$ used. As ${\gamma }_{{{{{{{{{\mathcal{M}}}}}}}}}^{\otimes N}}\ge 1-{c}^{N}$ should be interpreted as a distinguishability measure similar to the ones of quantum hypothesis testing^66–68^, it is rather its *asymptotic rate exponent*,  that quantifies metrological capabilities of ${{{{{{{{\mathcal{M}}}}}}}}}^{\otimes N}$ in the asymptotic *N* limit. Hence, we formally determine the lower bound  Nonetheless, the form of ${V}_{\vec{\Phi }}$ we use, and the discussion on its optimality we leave to the Supplementary Note E. Crucially, in case of the canonical multi-probe scenario of Fig. 4(a), we may directly conclude from Eq. (17) that:

#### Corollary 1

(Go-theorem for the HS with imperfect measurements and global control). For any non–information-erasing detection channel, the HS (${\Delta }^{2}{\tilde{\theta }}_{N} \sim 1/{N}^{2}$) can always be asymptotically attained, by choosing any global unitary ${V}_{\vec{\Phi }}$ such that Eq. (17) holds, and any pure input state with QFI ${{{{{{{\mathcal{F}}}}}}}}[{\psi }^{N}(\theta )] \sim \, {N}^{2}$ for ${N\to \infty }$.

Note that in the view of relations to “standard” noisy quantum metrology protocols^39^, ${V}_{\vec{\Phi }}$ required by Eq. (17) must not allow for its commutation as in (i) or (ii) of Fig. 3—as shown explicitly in the Supplementary Note D already for two qubits (*N* = 2), each measured projectively with bit-flip errors—so that the corresponding no-go theorems^41,42^ forbidding the HS no longer apply.

However, one should also verify whether the above corollary, relying on convergence (15), is not a “measure-zero” phenomenon. In particular, whether, if the assumption of state purity in Eq. (15) is dropped, the preservation of different scalings in *N* is still maintained. That is why, we prove the robustness of Thm. 1 by generalising it to the case of noisy (mixed) input states, which after *θ*-encoding take the form:18and can be interpreted in the canonical multi-probe scenario of Fig. 4(a) as *white noise* (or *global depolarisation*) of fixed strength 0 < *r* < 1 being admixed to a pure input state *ψ*^*N*^. Note that, in the picture of “standard” noisy metrology protocols, global depolarisation corresponds to correlated decoherence and, hence, the no-go theorems precluding the HS no longer apply. Nonetheless, all our claims hold if one replaces ${{\mathbb{1}}}_{{d}^{N}}/{d}^{N}$ in Eq. (18) by any product state. In particular, we prove (see Supplementary Note F) the following lemma:

#### Lemma 2

(Robustness of Thm. 1). For any mixed encoded state ${\rho }_{r}^{N}(\theta )$ of the form (18), and any detection channel that is non–information-erasing, the imperfect QFI about *θ* converges to the perfect QFI as ${N\to \infty}$:

19 $${{{{{{{{\mathcal{F}}}}}}}}}_{N}^{{{{{{{{\rm{(im)}}}}}}}}}\mathop{=}\limits_{N\to \infty }{{{{{{{\mathcal{F}}}}}}}}\left[{\rho }_{r}^{N}(\theta )\right]\mathop{=}\limits_{N\to \infty }r{{{{{{{\mathcal{F}}}}}}}}\left[{\psi }^{N}(\theta )\right].$$

The proof is very similar to that of Thm. 1, while it relies also (see Eq. (17)) on existence of lower bounds ${{{{{{{{\mathcal{F}}}}}}}}}_{N}^{{{{{{{{\rm{(im)}}}}}}}}}\ge {F}_{N}({V}_{\vec{\Phi }})\ge {F}_{N}^{\downarrow }({V}_{\vec{\Phi }},r)$, where ${F}_{N}^{\downarrow }({V}_{\vec{\Phi }},r)\to r{{{{{{{\mathcal{F}}}}}}}}[{\psi }^{N}(\theta )]$ as ${N\to \infty }$. Focussing on the asymptotic scaling of precision in the canonical multi-probe scenario, it directly follows that despite the white noise, if ${{{{{{{\mathcal{F}}}}}}}}[{\psi }^{N}(\theta )] \sim {N}^{2}$, then ${{{{{{{{\mathcal{F}}}}}}}}}_{N}^{{{{{{{{\rm{(im)}}}}}}}}} \sim r{N}^{2}$ and the HS is still attained.

As an example, let us explicitly discuss how the Thm. 1 and Lemma 2 apply in the canonical multi-qubit scenario of Fig. 4(a), in which ${U}_{\theta }^{N}={U}_{\theta }^{\otimes N}$ with *U*_*θ*_ = e^i*hθ*^ and *h* = *σ*_z_/2, while measurement imperfections arise due to a noisy detection channel ${{{{{{{\mathcal{P}}}}}}}}$ that flips the binary outcome for each qubit with probabilities p and q, respectively—as depicted within the inset of Fig. 1(c). Then, by initialising the probes in the GHZ state, ${\psi }^{N}=|{\psi }^{N}\rangle \langle {\psi }^{N}|$ with $|{\psi }^{N}\rangle=({|0\rangle }^{\otimes N}+{|1\rangle }^{\otimes N})/\sqrt{2}$, we find (see Supplementary Note E) a global control unitary ${V}_{\vec{\Phi }}$ for which the lower bound in Eq. (17) reads:

20 $${F}_{N}^{\downarrow }({V}_{\vec{\Phi }})	={N}^{2}\left[1-{(\sqrt{{\mathsf{p}}(1-{\mathsf{q}})}+\sqrt{{\mathsf{q}}(1-{\mathsf{p}})})}^{N}\right]\\ 	={N}^{2}\left[1-{{{{{{{{\rm{e}}}}}}}}}^{-\chi N}\right]$$

with $\chi \approx \frac{1}{4}\frac{{\left({\mathsf{p}}+{\mathsf{q}}-1\right)}^{2}}{{\mathsf{p}}\left(1-{\mathsf{p}}\right)+{\mathsf{q}}\left(1-{\mathsf{q}}\right)}$. The ultimate precision with *N* is attained and, hence, the HS—as illustrated in Fig. 5 for ${\mathsf{p}}=0.95$ and ${\mathsf{q}}=0.9$. Furthermore, we repeat the above procedure of finding ${V}_{\vec{\Phi }}$ to attain the ultimate asymptotic precision for an input GHZ state subjected to white noise according to Eq. (18). In such a setting, we determine analytically the required lower bound ${F}_{N}^{\downarrow }({V}_{\vec{\Phi }},r)$ (see Supplement Sections E, F), which we similarly depict in Fig. 5 for *r* = 0.7, together with the exact behaviour of ${F}_{N}({V}_{\vec{\Phi }})$ determined numerically. Note that an expression similar to Eq. (20) has been established for the noisy detection channel corresponding to Gaussian coarse-graining^35,37^, while in Methods we derive its form for lossy photonic interferometry with dark counts.

**Fig. 5:**
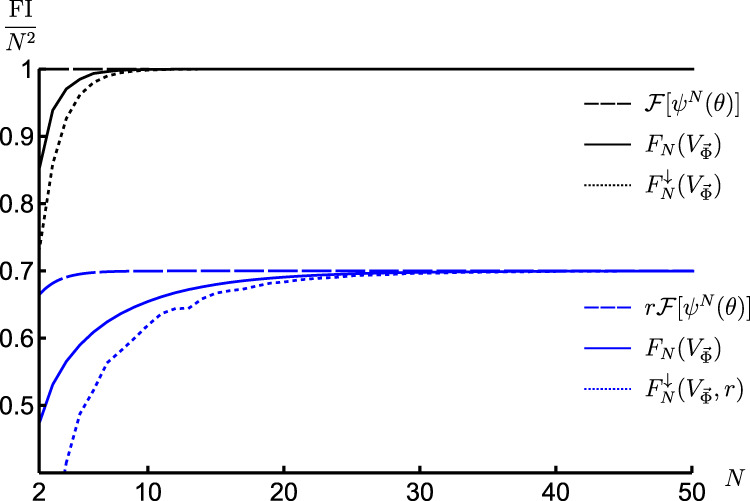
Attaining the ultimate HS of precision in presence of measurement imperfections and *global* unitary control. The case of phase estimation with *N* qubit probes is considered, which are initialised in a GHZ state, whereas the outcomes of ideal local measurements undergo an asymmetric bit-flip channel with ${\mathsf{p}}$ = 0.95 and ${\mathsf{q}}$ = 0.9. The black solid line is the exact (numerical) FI for a specific choice of the control global unitary ${V}_{\vec{\Phi }}$, while the black dotted line is its lower bound ${F}_{N}^{\downarrow }({V}_{\vec{\Phi }})$ defined in Eq. (20)—both converge to the optimal achievable ${{{{{{{\mathcal{F}}}}}}}}[{\psi }^{N}(\theta )]={N}^{2}$ (black dashed line). The family of lines in blue are the corresponding FIs for the case of a distorted GHZ state, with an admixture of white noise (with *r* = 0.7 in Eq. (18)) being added.

### Local control operations

We next turn our attention to canonical multi-probe scenarios with unitary encoding, ${\rho }^{N}(\theta )={{{{{{{{\mathcal{U}}}}}}}}}_{\theta }^{\otimes N}[{\rho }^{N}]$, in which only local control operations are allowed, ${\otimes }_{\ell=1}^{N}{{{{{{{{\mathcal{V}}}}}}}}}_{{\vec{\phi }}_{\ell }}^{(\ell )}$ with every ${{{{{{{{\mathcal{V}}}}}}}}}_{{\vec{\phi }}_{\ell }} \sim \, \{{V}_{{\vec{\phi }}_{\ell }}\}$ in Fig. 4(b), in order to verify whether these are already sufficient to compensate for measurement imperfections. We denote the corresponding imperfect channel QFI as ${\bar{{{{{{{{\mathcal{F}}}}}}}}}}_{N}^{({{{{{{{\rm{im}}}}}}}},{{{{{{{\rm{l}}}}}}}})}$. Crucially, in such a case the quantum metrology protocol of Fig. 4(a) can be recast using the formalism of *quantum-classical channels*^54^. For each probe we introduce a fictitious ∣*X*∣-dimensional Hilbert space spanned by orthogonal states $\left|x\right\rangle$ that should be interpreted as flags marking different outcomes *x* being observed. As a result, focusing first on the evolution of a single probe illustrated in Fig. 4(c), the observed outcome of the imperfect measurement may be represented by a classical state ${\rho }_{{{{{{{{\rm{cl}}}}}}}}}(\theta,\vec{\phi })={\sum }_{x}{q}_{\theta,\vec{\phi }}(x)|x\rangle \langle x|$, with the transformation $\rho \to {\rho }_{{{{{{{{\rm{cl}}}}}}}}}(\theta,\vec{\phi })={\Lambda }_{\theta,\vec{\phi }}[\rho ]$ governed by the quantum-classical channel ${\Lambda }_{\theta,\vec{\phi }}$. Then, in the canonical multi-probe scenario of Fig. 4(a), each of the *N* probes is independently transformed by the quantum-classical channel ${\Lambda }_{\theta,{\vec{\phi }}_{\ell }}={\Lambda }_{{{{{{{{\mathcal{M}}}}}}}}}\circ {{{{{{{{\mathcal{V}}}}}}}}}_{{\vec{\phi }}_{\ell }}\circ {{{{{{{{\mathcal{U}}}}}}}}}_{\theta }$ (see Supplementary Note G for the explicit form of ${\Lambda }_{{{{{{{{\mathcal{M}}}}}}}}}$), and the overall input state undergoes ${\rho }^{N}{\to \bigotimes }_{\ell=1}^{N}{\Lambda }_{\theta,{\vec{\phi }}_{\ell }}^{(\ell )}[{\rho }^{N}]={\rho }_{{{{{{{{\rm{cl}}}}}}}}}^{N}(\theta,\{{\vec{\phi }}_{\ell }\})$, where the output classical state ${\rho }_{{{{{{{{\rm{cl}}}}}}}}}^{N}(\theta,\{{\vec{\phi }}_{\ell }\})$ is now diagonal in the total *N* × ∣*X*∣-dimensional fictitious Hilbert space—describing the probability distribution of all the *N* measurement outcomes. By treating quantum-classical channels as a special class of quantum maps that output diagonal states in a fixed basis, we apply the *channel extension* (CE) method introduced in Refs. 40,42,69 in order to construct the so-called *CE-bound*, i.e.,

21 $${F}_{N}\le {F}_{N}^{({{{{{{{\rm{CE}}}}}}}})}(\{{\vec{\phi }}_{\ell }\}).$$

While leaving the technical derivation and expression of ${F}_{N}^{({{{{{{{\rm{CE}}}}}}}})}(\{{\vec{\phi }}_{\ell }\})$ to Methods, let us emphasise that the CE-bound (21) is independent of the probes’ state *ρ*^*N*^ that is now arbitrary and potentially mixed. Furthermore, it allows even for extending the input—hence, the name—to include extra *N* ancillae, which do not undergo the parameter encoding but can be prepared in a state entangled with the probes before being (ideally) measured to further enhance the precision. Still, the bound (21) depends, in principle, on the setting of each (local) measurement ${\vec{\phi }}_{\ell }$, as well as the parameter *θ* itself. Nonetheless, we prove (see Methods for the prescription and Supplementary Note G for further details) the following lemma:

#### Lemma 3

(Linear scaling of the asymptotic CE bound). For unitary encoding ${U}_{\theta }^{N}={U}_{\theta }^{\otimes N}$ with *U*_*θ*_ = *e*^i*hθ*^, we may further define the asymptotic CE bound ${F}_{N}^{({{{{{{{\rm{CE}}}}}}}},{{{{{{{\rm{as}}}}}}}})}$, which satisfies ${F}_{N}\le {F}_{N}^{({{{{{{{\rm{CE}}}}}}}})}(\{{\vec{\phi }}_{\ell }\})\le {F}_{N}^{({{{{{{{\rm{CE}}}}}}}},{{{{{{{\rm{as}}}}}}}})}(\{{\vec{\phi }}_{\ell }\})$ and $\mathop{\lim }\limits_{N\to \infty }{F}_{N}^{({{{{{{{\rm{CE}}}}}}}})}={F}_{N}^{({{{{{{{\rm{CE}}}}}}}},{{{{{{{\rm{as}}}}}}}})}$, whenever there exists a set of Hermitian operators ${\{{A}_{x}({\vec{\phi }}_{\ell })\}}_{x}$ such that for each ${\vec{\phi }}_{\ell }$:

22 $$h=\mathop{\sum }\limits_{x=1}^{|X|}{V}_{{\vec{\phi }}_{\ell }}^{{{{\dagger}}} }\sqrt{{M}_{x}}{A}_{x}({\vec{\phi }}_{\ell })\sqrt{{M}_{x}}{V}_{{\vec{\phi }}_{\ell }}.$$

Moreover, upon optimising ${F}_{N}^{({{{{{{{\rm{CE}}}}}}}},{{{{{{{\rm{as}}}}}}}})}$ over all local control unitaries, we obtain23where 0≤*c* < *∞* is an *N*-independent constant factor that is fully determined by a single copy of the channel ${\Lambda }_{\theta,\vec{\phi }}$, and Eq. (23) applies for any local control $\{{\vec{\phi }}_{\ell }\}$.

Note that the structure of the derivation in Methods suggests Eq. (23) to hold also for adaptive protocols, in which the *ℓ*th control unitary ${{{{{{{{\mathcal{V}}}}}}}}}_{{\vec{\phi }}_{\ell }}$ may be adjusted based on the outcomes of local measurements performed on the previous probes (1, 2,…,*ℓ* − 1). Meanwhile, although the condition (22) may look abstract, it actually has an intuitive meaning, when considering imperfect measurement that arises due to some noisy detection channel ${{{{{{{\mathcal{P}}}}}}}}$, such that *M*_*x*_ = ∑_*i*_*p*(*x*∣*i*)Π_*i*_. Let us call a detection channel ${{{{{{{\mathcal{P}}}}}}}}$ *non-trivial* if its transition probabilities *p*(*x*∣*i*) are such that for all pairs of ‘inaccessible’ outcomes $i,i^{\prime}$, there is at least one ‘observable’ outcome *x* such that $p(x|i)p(x|i^{\prime} ) > 0$. Then, we have (see Supplementary Note H for an explicit proof):

#### Corollary 2

(No-go theorem for HS with imperfect measurements and local control). Consider the canonical multi-probe scenario depicted in Fig. 4(a) that incorporates a non-trivial noisy detection channel ${{{{{{{\mathcal{P}}}}}}}}$, whose impact one may only compensate for by means of local control unitaries, see Fig. 4(b). Then, the condition (22) can always be satisfied and, as Eq. (23) implies that ${\Delta }^{2}{\tilde{\theta }}_{N}\ge \varepsilon /N$ for some *ε* > 0, the HS cannot be attained with the MSE following at best the SS.

**Fig. 6:**
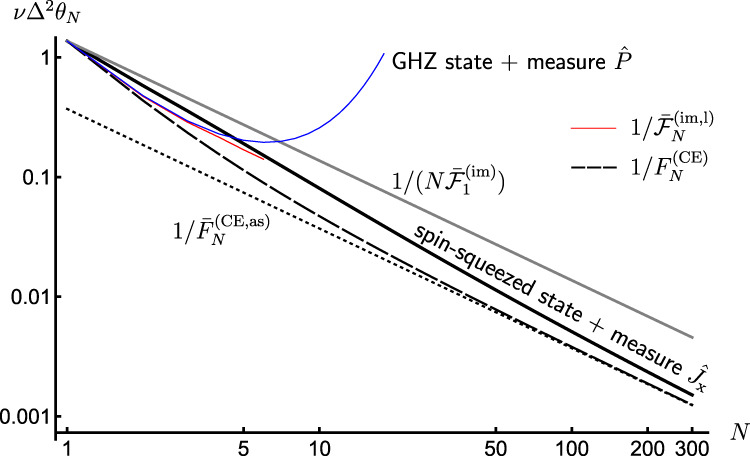
Attaining the optimal SS of precision in presence of measurement imperfections and *local* unitary control. The thick solid black line depicts the MSE in estimating phase *θ* from an imperfect measurement of the angular momentum operator ${\hat{J}}_{{{{{{{{\rm{x}}}}}}}}}$, while the *N* qubit probes are prepared in a one-axis spin-squeezed state^57^, optimised by local control (see Methods). The noisy detection channel corresponds to an asymmetric bit-flip map with probabilities ${\mathsf{p}}=0.95$ and ${\mathsf{q}}=0.9$. The dotted black line denotes the asymptotic CE bound with ${\bar{F}}_{N}^{({{{{{{{\rm{CE}}}}}}}},{{{{{{{\rm{as}}}}}}}})}$ given by Eq. (24), while the thin red solid line is the exact achievable precision $1/{\bar{{{{{{{{\mathcal{F}}}}}}}}}}_{N}^{({{{{{{{\rm{im}}}}}}}},{{{{{{{\rm{l}}}}}}}})}$, which we compute numerically up to *N* = 6 by brute-force heuristic methods. The dashed black line corresponds to $1/{F}_{N}^{{{{{{{{\rm{(CE)}}}}}}}}}$ in Eq. (21) applicable in absence of control (${\forall }_{\ell }:\ {V}_{{\vec{\phi }}_{\ell }}^{(\ell )}={\mathbb{1}}$). At small *N* (≲4), the ultimate precision can be attained by performing (imperfect) parity measurements with input GHZ states (thin blue line). For comparison, we also include the optimal precision attained by uncorrelated probe states, $1/(N{\bar{{{{{{{{\mathcal{F}}}}}}}}}}_{1}^{({{{{{{{\rm{im}}}}}}}})})$, (solid gray).

In order to illustrate our result, let us consider again the canonical multi-qubit scenario with every qubit being subject to a projective measurement, whose outcome suffers an asymmetric bit-flip noise parametrised by ${{{\rm{p}}}}$ and ${{{\rm{q}}}}$, see Fig. 4. We evaluate the corresponding asymptotic CE bound (see Supplementary Note I):

24 $${\bar{F}}_{N}^{({{{{{{{\rm{CE}}}}}}}},{{{{{{{\rm{as}}}}}}}})}=N{\left(\frac{\sqrt{{\mathsf{p}}(1-{\mathsf{p}})}-\sqrt{{\mathsf{q}}(1-{\mathsf{q}})}}{{\mathsf{p}}-{\mathsf{q}}}\right)}^{2},$$

which, however, must be further verified to be asymptotically attainable. Indeed, in Methods we show this to be true even for a simple inference strategy, in which an (imperfect) measurement of the total angular momentum ${\hat{J}}_{{{{{{{{\rm{x}}}}}}}}}$ is performed with the *N* probes prepared in an *one-axis spin-squeezed state*^57^ with the correct amount of squeezing and rotation, as illustrated graphically in Fig. 6. Moreover, we demonstrate that up to *N* ≲ 4, the ultimate precision determined numerically can also be attained by considering the parity observable incorporating the imperfect measurement, with probes being prepared in a GHZ state rotated at an optimal angle (see Supplementary Note J for details).

Note that our recipe to construct the bound (23) applies generally, not relying on any properties of the imperfect measurement ${{{{{{{\mathcal{M}}}}}}}}$, e.g., see Methods for its application to the photonic setting in which ∣*X*∣>*d*. Still, for the above multi-qubit case with detection bit-flip noise, for which *d* = ∣*X*∣ = 2, we observe (see Supplementary Note I) that the corresponding bound (24) can be postulated based on a conjecture of the optimal local controls corresponding to *phase-covariant* rotations^65^, what allows then to invoke the results of “standard” noisy metrology^39^.

## Discussion

We have analysed the impact of measurement imperfections on quantum metrology protocols and, in particular, the prospects of recovering the ideal quantum enhancement of sensitivity, e.g., the Heisenberg scaling (HS) of precision, despite the readout noise. The contrasting results obtained with global or local control operations can be understood by the following simple intuition.

With global control operations available, one may effectively construct a global measurement tailored to the two-dimensional subspace containing the information about any tiny changes of the parameter. Importantly, thanks to the exponential increase of the overall dimension with the number of probes, one may then distinguish (exponentially) better and better the two states lying in this two-dimensional subspace, within which the effective amount of readout noise diminishes, and the perfect optimal scaling prevails. Our work, thus, motivates explicitly the use of variational approaches in identification of such global unitary control not only at the level of state preparation^70,71^, but also crucially in the optimisation of local measurements^31,72^. On the other hand, it demonstrates that control operations form the key building-block in fighting measurement imperfections in quantum metrology. Although we have provided control strategies that allow to maintain the HS both in the qubit and photonic settings, these employ *N*-body interactions, while it is known that 2-body interactions suffice in presence of Gaussian blurring arising in cold-atom experiments^34–37^. Thus, we believe that our results open an important route of investigating the complexity of such global control required, depending on the form of the readout noise encountered.

On the contrary, there is no exponential advantage gained when only local control operations are available. Hence, as the overall amount of noise also rises limitlessly as we increase the number of probes, the asymptotic scaling of sensitivity is constrained to be classical. Note that this conclusion is valid also in the Bayesian scenario, as by the virtue of the Bayesian CRB^73^ also the *average* MSE is then lower-bounded by $\langle {\Delta }^{2}\tilde{\theta } \rangle \, \approx \, 1/\langle {\bar{{{{{{{{\mathcal{F}}}}}}}}}}_{N}^{({{{{{{{\rm{im}}}}}}}})}\rangle \, \gtrsim \, 1/N$, where 〈…〉 denotes now the averaging over some prior distribution of the parameter.

Finally, although we have primarily focussed here on phase-estimation protocols, let us emphasise once more that Lemma 1 and, hence, Thm. 1 applies to any quantum metrology scheme involving pure states and imperfect measurements. Hence, it holds also when sensing, e.g., ‘critical’ parameters at phase transitions with noisy detection^60^. Still, generalisation to the case with mixed states (beyond product-state admixtures) remains open. This would allow us, for instance, to approach quantum thermometry protocols utilising thermalised (Gibbs) probe states with the temperature being then estimated despite coarse-graining of measurements^74^. In such cases, one should then also characterise the (mixed) states for which the imperfect QFI is actually guaranteed to converge to the perfect QFI in the asymptotic *N* limit.

## Methods

### Phase sensing with an NV centre

Within this protocol, the NV centre is firstly initialised into some superposition state $\rho=|\psi \rangle \langle \psi |$ of the *m*_*s*_ = 0 (corresponding to $|0\rangle$) and *m*_*s*_ = 1 (corresponding to $\left|1\right\rangle$) ground-state energy levels with help of a Ramsey pulse. The NV spin is then used to sense a magnetic field of strength *B* in the z-direction for time *t* (usually chosen to be as long as the decoherence allows for, i.e., ${T}_{2}^{*}$ or *T*_2_ for either static or alternating fields), gaining the relative phase *θ* = −*tγB*, where *γ* is the gyromagnetic ratio characteristic to the NV centre^51,75^. For our purpose we assume the evolution time to be perfectly known (and so the gyromagnetic ratio), so that the problem of estimating the field strength *B* is effectively equivalent to estimating the relative phase *θ*. Effectively then, the encoding channel is ${{{{{{{{\mathcal{U}}}}}}}}}_{\theta } \sim \, \{{U}_{\theta }={{{{{{{{\rm{e}}}}}}}}}^{{{{{{{{\rm{i}}}}}}}}h\theta }\}$, with *h* = *σ*_z_/2, where *σ*_*ℓ*_ is the usual Pauli-*ℓ* operators with *ℓ* = x,y,z.

In order to read out *θ*, a measurement is performed on the NV spin. Since the energy levels are fixed and not directly accessible, a microwave pulse is again applied to rotate the qubit basis, such that the phase is now carried in state populations instead. Afterwards, the NV-spin is optically excited, so that $|0 \rangle \to |0^{\prime} \rangle$ and $|1 \rangle \to |1^{\prime} \rangle$, where $\left|0^{\prime} \right\rangle$ and $\left|1^{\prime} \right\rangle$ correspond respectively to the *m*_*s*_ = 0 and *m*_*s*_ = 1 excited energy levels. While the optical transitions between the two *m*_*s*_ = 0 energy levels are essentially exclusive, there is a metastable singlet state to which the excited *m*_*s*_ = 1 energy state can decay non-radiatively. As a consequence, when performing now the measurement of photon emissions in such a spin-dependent fluorescence process over a designated time window, a dark signal indicates the original NV spin to be projected onto $|1 \rangle$, while a bright signal corresponds to the projection onto $\left|0\right\rangle$. That is, within our general formalism, ${\Pi }_{1}=|0 \rangle \langle 0|$ and ${\Pi }_{2}=|1 \rangle \langle 1|$, so that after fixing the second Ramsey pulse to e.g., ${V}_{\vec{\phi }}={{{{{{{{\rm{e}}}}}}}}}^{{{{{{{{\rm{i}}}}}}}}\pi {\sigma }_{{{{{{{{\rm{x}}}}}}}}}/4}$, we have ${\Pi }_{1(2),\vec{\phi }}=|\pm \rangle \langle \pm |$, with ${\sigma }_{{{{{{{{\rm{x}}}}}}}}}\left|\pm \right\rangle=\pm \left|\pm \right\rangle$.

The bright versus dark distinction is however not perfect: the *m*_*s*_ = 1 excited state could still decay radiactively into the ground state, with the dark signal typically reducible to about 65% of the bright signal. Moreover, as the photon emissions are spontaneous and random, the same photon-number being recorded can actually come from both the dark and bright signals, albeit with different probabilities. These for the readout of an NV-centre are modelled as two Poissonian distributions of distinct means, depending also on the number of QND repetitions^76,77^, often approximated by Gaussians^53^—see Fig. 1(c). As a result, the ‘observed’ outcomes correspond to the number of collected photons, *X* = {0, 1, 2,...}, which are distributed according to the two Poissonian distributions $p(x|1)={{{{{{{{\rm{e}}}}}}}}}^{-{\lambda }_{\left|0\right\rangle }}{({\lambda }_{\left|0\right\rangle })}^{x}/x!$ and $p(x|2)={{{{{{{{\rm{e}}}}}}}}}^{-{\lambda }_{|1 \rangle }}{({\lambda }_{|1 \rangle })}^{x}/x!$, whose means, ${\lambda }_{|0 \rangle }$ and ${\lambda }_{|1 \rangle }$, differ depending on which energy state the NV spin was previously projected onto by ${\Pi }_{1,\vec{\phi }}$ or ${\Pi }_{2,\vec{\phi }}$.

### Estimating the FI with binning strategies

In this section we discuss in more depth the binning method for estimating the FI for the single-probe scenario. Firstly, let us remark that for the strategy with two bins and *δ* = 0 in Eq. (11), we deal with a symmetric bit-flip channel mixing the two outcomes regardless of the choice of measurement basis. In this special case, the noisy detection affecting the measurement has exactly the same effect as if a dephasing noise acted before an ideal measurement. Indeed, taking the limit *δ*→0 in Eqs. (11) and (12), the optimal state angle becomes *ϕ*_opt_ = −*θ* and ${\bar{F}}_{{{{{{{{\rm{2-bin}}}}}}}}}^{*}={\eta }^{2}$, agreeing with the well known result for the dephasing noise^41,42^. Still, for any asymmetric bit-flip detection with p ≠ q, the imperfect measurement model can no longer be interpreted as decoherence affecting rather the parameter encoding.

Secondly, let us note that when adopting a binning strategy one can freely choose the boundaries that define the bins. For binary binning ${F}_{{{{{{{{\rm{2-bin}}}}}}}}}^{*}$ and ${\bar{F}}_{{{{{{{{\rm{2-bin}}}}}}}}}^{*}$ depend on a single boundary (“threshold”^52,53^) x* via the parameters *δ* and *η*, so that upon maximising the choice of x* we can also define:

25 $${F}_{2{{{{{{{\rm{-bin}}}}}}}}}=\mathop{\max }\limits_{{x}^{*}}{F}_{{{{{{{{\rm{2-bin}}}}}}}}}^{*},\quad {\bar{F}}_{2{{{{{{{\rm{-bin}}}}}}}}}=\mathop{\max }\limits_{{x}^{*}}{\bar{F}}_{2{{{{{{{\rm{-bin}}}}}}}}}^{*}.$$

Intuitively, one should choose x* such that the distributions *p*(*x*∣*i*) have the smallest overlap with the bins that yield errors in inferring the outcome *i*. Indeed, for the NV-sensing problem, the optimal choice of x* is located around the point where the two Poissonians cross in Fig. 4(a), *p*(*x*∣1) = *p*(*x*∣2), so that the probability of *x* < x* occurring when *i* = 2 is minimised (and similarly for *x* > x* when *i* = 1). More generally, in case of *k*-binning strategy with the corresponding FIs: ${F}_{k{{{{{{{\rm{-bin}}}}}}}}}^{*},{\bar{F}}_{k{{{{{{{\rm{-bin}}}}}}}}}^{*},{F}_{k{{{{{{{\rm{-bin}}}}}}}}},{\bar{F}}_{k{{{{{{{\rm{-bin}}}}}}}}}$; constituting natural generalisations of Eqs. (11) and (25) and x* being now a (*k* − 1)-entry vector specifying boundaries between all the bins. Consistently, the more bins are considered the closer the corresponding FIs are to the exact *F* (and ${\bar{{{{{{{{\mathcal{F}}}}}}}}}}^{({{{{{{{\rm{im}}}}}}}})}$) defined in Eq. (9).

**Fig. 7:**
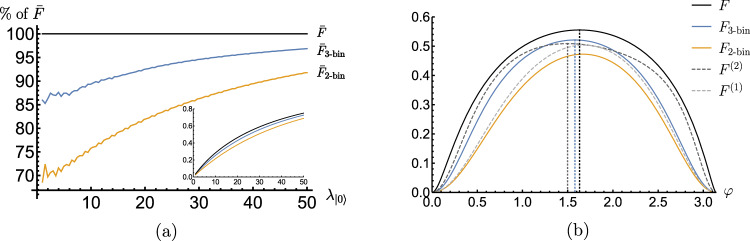
FI for phase *θ* with imperfect measurements affected by the Poissonian noise. **a** With binning strategies: the corresponding FI—${\bar{F}}_{{{{{{{{\rm{2-bin}}}}}}}}}$ (orange) and ${\bar{F}}_{{{{{{{{\rm{3-bin}}}}}}}}}$ (blue) with optimal binning into two and three categories, respectively—compared against the exact ${\bar{{{{{{{{\mathcal{F}}}}}}}}}}^{({{{{{{{\rm{im}}}}}}}})}$ (ratio in %) computed by performing large enough cut-off (*x*≤100) in Eq. (9). The ratio of means for the Poissonnian distributions is set to ${\lambda }_{|1 \rangle }/{\lambda }_{|0 \rangle }=0.65$^76^, while ${\lambda }_{|0 \rangle }$ is varied. The inset shows the absolute values of FIs. **b** With binning strategies and the moment method: The FIs (*F*–black, *F*_2-bin_–orange, *F*_3-bin_–blue) presented now as a function of the input state angle *ϕ* = *φ* − *θ* (for ${\lambda }_{|1 \rangle }/{\lambda }_{|0 \rangle }=0.65$ and ${\lambda }_{\left|0\right\rangle }=27$^76^) in comparison to the lower bounds on *F* constructed by taking into account up to the second (*F*^(1)^, light gray dash) and fourth moment (*F*^(2)^, dark gray dash) of the distribution describing the observed outcomes, ${q}_{\theta,\vec{\phi }}$. The vertical dotted lines indicate the (optimal) state angle at each of the respective quantities is maximised. Note that when the measurement is perfect, the FI is unity for all choices of the angle *φ* (not shown).

For illustration, we revisit the case of sensing the relative phase with a NV spin, with the measurement suffering a Poissonian noise. In Fig. 7(a), the performances of the optimal FIs for two- and three-binning strategies, ${\bar{F}}_{{{{{{{{\rm{2-bin}}}}}}}}}$ and ${\bar{F}}_{{{{{{{{\rm{3-bin}}}}}}}}}$, are investigated and compared against the exact maximal FI, $\bar{F}$, which we numerically approximate by maximising *F* with *x* summed in Eq. (9) up a cut-off large enough (*x* ≤ 100) to be effectively ignorable. Within the plot the optical contrast is fixed to the typical experimental value of 0.35, i.e., ${\lambda }_{\left|1\right\rangle }/{\lambda }_{\left|0\right\rangle }=0.65$^76^, while the FIs are plotted as a fraction of $\bar{F}$ for different values of ${\lambda }_{\left|0\right\rangle }$, which can be varied experimentally by having different repetitions of the QND measurement^52,53,76^. From the figure, we see that despite their simplicity, the strategy of binning into just two (orange) or three outcomes (blue) is pretty effective, as they are able to account for at least 70% of $\bar{F}$, and reach 90% with increasing ${\lambda }_{\left|0\right\rangle }$ already at ${\lambda }_{\left|0\right\rangle }\approx 50$. Then, similar to Fig. 2, in Fig. 7(b) we further include *F*_3-bin_ in the plot of FI for different choices of input state angles *ϕ* = *φ* − *θ*, for the specific value of ${\lambda }_{\left|0\right\rangle }=27$ which has been experimentally used in Ref. 76.

### Lower-bounding the FI via the moments of a probability distribution

It can be shown (see Supplementary Note K for derivation) that by including up to the first 2*K* moments of the distribution ${q}_{\theta,\vec{\phi }} \sim \, \{{q}_{\theta,\vec{\phi }}(x)\}$ a lower bound on the corresponding FI, ${F}^{(K)}\le F[{q}_{\theta,\vec{\phi }}]$ in Eq. (2), can be constructed that corresponds to an inner product of two *K* × *K* matrices:26where *B* = ***bb***^*T*^ with ${{{{{{{\boldsymbol{b}}}}}}}}={\left(0,\dot{{\mathbb{E}}}[x],\cdots,\dot{{\mathbb{E}}}[{x}^{K}]\right)}^{T}$, and

27 $$A=\left(\begin{array}{cccc}1&{\mathbb{E}}[x]&\cdots &{\mathbb{E}}[{x}^{K}]\\ {\mathbb{E}}[x]&{\mathbb{E}}[{x}^{2}]&\cdots &{\mathbb{E}}[{x}^{K+1}]\\ \vdots &&\ddots &\\ {\mathbb{E}}[{x}^{K}]&{\mathbb{E}}[{x}^{K+1}]&\cdots &{\mathbb{E}}[{x}^{2K}]\end{array}\right)$$

with ${\mathbb{E}}[{x}^{j}]={\sum }_{x}{q}_{\theta,\vec{\phi }}(x){x}^{j}$, $\dot{{\mathbb{E}}}[{x}^{j}]={\sum }_{x}{\dot{q}}_{\vec{\phi },\theta }(x){x}^{j}$ and ${\dot{q}}_{\vec{\phi },\theta }(x)={\partial }_{\theta }{q}_{\theta,\vec{\phi }}(x)$. Note that for the simplest case of *K* = 1, one obtains ${F}^{(1)}=\dot{{\mathbb{E}}}{[x]}^{2}/({\mathbb{E}}[{x}^{2}]-{\mathbb{E}}{[x]}^{2})=|{\partial }_{\theta } < X > {|}^{2}/{{{{{{{\rm{Var[X]}}}}}}}}$ that constitutes the standard lower-bound on *F* formed by considering the error-propagation formula applied to the distribution of the outcomes *X*^56^. Evidently, we have the hierarchy *F*^(*K*)^≤*F*^(*K*+1)^, whereby the more we know about its moments, the more we recover the underlying probability distribution, and *F*^(*K*)^ converges to $F[{q}_{\theta,\vec{\phi }}]$. For demonstration on the improvement of FI lower bound with higher moments considered, in Fig. 7(b), we reproduce Fig. 2, with now *F*^(2)^ included as well.

### Upper-bounding the imperfect QFI given the *G*-covariance of a conjugate-map decomposition

We formalise the condition when the results of “standard” noisy metrology^39^, in which the decoherence affects the parameter encoding, can be applied to the setting of imperfect measurements by resorting to the notion of symmetry, in particular, the *G*-covariance^78,79^.

Given a compact group *G*, we say that a quantum channel ${{{{{{{\mathcal{E}}}}}}}}$ is *G*−*covariant* if^78,79^

28 $${\forall }_{g\in G}:{{{{{{{\mathcal{E}}}}}}}}\circ {{{{{{{{\mathcal{V}}}}}}}}}_{g}={{{{{{{{\mathcal{W}}}}}}}}}_{g}\circ {{{{{{{\mathcal{E}}}}}}}},$$

where ${{{{{{{{\mathcal{V}}}}}}}}}_{g}$, ${{{{{{{{\mathcal{W}}}}}}}}}_{g}$ form some unitary representations of *G*.

Now, by denoting the FI in Eq. (5) as $F[{q}_{\theta,\vec{\phi }}]\equiv F[{{{{{{{{\mathcal{V}}}}}}}}}_{\vec{\phi }}[\rho (\theta )],{{{{{{{\mathcal{M}}}}}}}}]$ to separate its dependence on the state and the POVM, we formulate the following observations:

#### Observation 1

(Imperfect measurement with a *G*-covariant conjugate-map decomposition). Given an imperfect measurement with a conjugate-map decomposition ${{{{{{{\mathcal{M}}}}}}}}={\Lambda }^{{{{\dagger}}} }[\Pi ]$, and a parametrised state *ρ*(*θ*), if the following conditions are satisfied:

(a) Λ is covariant with respect to a compact group *G*.

(b) the optimal unitary that yields the imperfect QFI (${{{{{{{{\mathcal{V}}}}}}}}}_{{{{{{{{\rm{opt}}}}}}}}}$ in Fig. 3) is guaranteed to be in *G*, so that

29 $${{{{{{{{\mathcal{F}}}}}}}}}^{{{{{{{{\rm{(im)}}}}}}}}}=\mathop{\max }\limits_{g\in G}F\left[{{{{{{{{\mathcal{V}}}}}}}}}_{g}[\rho (\theta )],{\Lambda }^{{{{\dagger}}} }[\Pi ]\right],$$

then

30 $${{{{{{{{\mathcal{F}}}}}}}}}^{{{{{{{{\rm{(im)}}}}}}}}}\le {{{{{{{\mathcal{F}}}}}}}}\left[\Lambda [\rho (\theta )]\right]$$

If further *G* = *SU*(*d*), then equality in Eq. (30) is assured.

Moreover, if the parameter encoding is provided in a form of a quantum channel, $\rho (\theta )={{{{{{{{\mathcal{E}}}}}}}}}_{\theta }[\rho ]$, and the optimal unitary ${{{{{{{{\mathcal{V}}}}}}}}}_{{{{{{{{\rm{opt}}}}}}}}}$ remains within *G* for the optimal input state, the upper bound (30) applies also to the corresponding imperfect channel QFI, i.e., ${\bar{{{{{{{{\mathcal{F}}}}}}}}}}^{({{{{{{{\rm{im}}}}}}}})}\le \bar{{{{{{{{\mathcal{F}}}}}}}}}[\Lambda \circ {{{{{{{{\mathcal{E}}}}}}}}}_{\theta }]$.

However, in case the parameter encoding satisfies the *G*-covariance property itself, we independently have that:

#### Observation 2

(Imperfect channel QFI for *G*-covariant parameter encodings). Given an imperfect measurement with some conjugate-map decomposition ${{{{{{{\mathcal{M}}}}}}}}={\Lambda }^{{{{\dagger}}} }[\Pi ]$ and the parameter encoding $\rho (\theta )={{{{{{{{\mathcal{E}}}}}}}}}_{\theta }[\rho ]$, if the following conditions are satisfied:

(a) both ${{{{{{{{\mathcal{E}}}}}}}}}_{\theta }$ and ${\dot{{{{{{{{\mathcal{E}}}}}}}}}}_{\theta }\equiv {\partial }_{\theta }{{{{{{{{\mathcal{E}}}}}}}}}_{\theta }$ are *G*-covariant.

(b) the optimal unitary that yields the optimal channel QFI (${{{{{{{{\mathcal{V}}}}}}}}}_{{{{{{{{\rm{opt}}}}}}}}}$ in Fig. 3) is guaranteed to be in *G*, so that

31 $${\bar{{{{{{{{\mathcal{F}}}}}}}}}}^{({{{{{{{\rm{im}}}}}}}})}=\mathop{\max }\limits_{\rho }\mathop{\max }\limits_{g\in G}F\left[{{{{{{{{\mathcal{V}}}}}}}}}_{g}[{{{{{{{{\mathcal{E}}}}}}}}}_{\theta }[\rho ]],{\Lambda }^{{{{\dagger}}} }[\Pi ]\right];$$

then

32 $${\bar{{{{{{{{\mathcal{F}}}}}}}}}}^{({{{{{{{\rm{im}}}}}}}})}\le \bar{{{{{{{{\mathcal{F}}}}}}}}}[\Lambda \circ {{{{{{{{\mathcal{E}}}}}}}}}_{\theta }].$$

We refer the reader to the Supplementary Note D for explicit proofs and further discussions of the above conditions.

### Upper-bounding the FI with the CE method for quantum-classical channel

A thorough account on the CE method is available at Refs. 40,42,69; here we simply highlight the general idea. In the CE method, when the probe state *ρ* undergoes an effective encoding described by a given channel ${{{{{{{{\mathcal{E}}}}}}}}}_{\theta }$, such that $\rho (\theta )={{{{{{{{\mathcal{E}}}}}}}}}_{\theta }[\rho ]$, the corresponding FI for *θ* is bounded by considering an enlarged space with a corresponding input state *ρ*_ext_, such that $\mathop{\max }\limits_{\rho }{{{{{{{\mathcal{F}}}}}}}}[\rho (\theta )]\le \mathop{\max }\limits_{{\rho }_{{{{{{{{\rm{ext}}}}}}}}}}{{{{{{{\mathcal{F}}}}}}}}[({{{{{{{{\mathcal{E}}}}}}}}}_{\theta }\otimes {\mathbb{1}})[{\rho }_{{{{{{{{\rm{ext}}}}}}}}}]]$, where the r.h.s. can be shown to be equal to $4\mathop{\min }\limits_{\tilde{\kappa }}\parallel {\alpha }_{\tilde{\kappa }}\parallel$, with $\tilde{\kappa }=\{{\tilde{\kappa }}_{i}\}$ denoting all the equivalent sets of Kraus operators for 

In order to apply the CE method to the canonical multi-probe metrology scheme with local control unitaries and local imperfect measurements, which has the corresponding product quantum-classical channel  with ${\Lambda }_{\theta,\vec{\phi }}={\Lambda }_{{{{{{{{\mathcal{M}}}}}}}}}\circ {{{{{{{{\mathcal{V}}}}}}}}}_{\vec{\phi }}\circ {{{{{{{{\mathcal{U}}}}}}}}}_{\theta }$ as depicted in Fig. 4(b), we first specify the ‘canonical’ set of Kraus operators for ${\Lambda }_{\theta,\vec{\phi }}$, $K(\theta,\vec{\phi })=\{{K}_{x,j}(\theta,\vec{\phi })=|x \rangle \langle j|\sqrt{{U}_{\theta }^{{{{\dagger}}} }{M}_{x,\vec{\phi }}{U}_{\theta }}\}$, given some orthonormal basis of states ${\{|j \rangle \}}_{j=1}^{d}$ spanning the qudit (*d*-dimensional) probe space. Importantly also, as the output classical state is diagonal in the flag basis, its QFI corresponds just to the (classical) FI of the eigenvalue distribution^16^ which we denote simply as ${F}_{N}={{{{{{{\mathcal{F}}}}}}}}[{\rho }_{{{{{{{{\rm{cl}}}}}}}}}^{N}(\theta,\vec{\phi })]$, with the corresponding (quantum-classical) channel QFI reads  Hence, upon further restricting the domain of minimisation over $\tilde{{\kappa }_{i}}$, where we only consider Kraus operators of ${{{{{{{\mathcal{E}}}}}}}}(\theta,\{{\vec{\phi }}_{\ell }\})$ with the product structure ${\tilde{\kappa }}_{i}(\theta,\{{\vec{\phi }}_{\ell }\}){=\bigotimes }_{\ell=1}^{N}{\tilde{K}}_{{x}_{\ell },{j}_{\ell }}^{(\ell )}(\theta,{\vec{\phi }}_{\ell })$, where $\tilde{K}(\theta,{\vec{\phi }}_{\ell })=\{{\tilde{K}}_{{x}_{\ell },{j}_{\ell }}^{(\ell )}(\theta,{\vec{\phi }}_{\ell })\}$ is the set of Kraus operators for ${\Lambda }_{\theta,{\vec{\phi }}_{\ell }}^{(\ell )}$, it is then straightforward to arrive at

33 $${F}_{N}\le 4\mathop{\min }\limits_{\{\tilde{K}(\theta,{\vec{\phi }}_{\ell })\}}\left |\left| \mathop{\bigoplus }\limits_{\ell=1}^{N}{\alpha }_{\tilde{K}(\theta,{\vec{\phi }}_{\ell })}^{(\ell )}+\mathop{\bigoplus }\limits_{\ell \, \ne \, m}^{N}{\beta }_{\tilde{K}(\theta,{\vec{\phi }}_{\ell })}^{(\ell )}{\beta }_{\tilde{K}(\theta,{\vec{\phi }}_{m})}^{(m)}\right | \right |,$$

where3435with ${\dot{\tilde{K}}}_{x,j}\equiv {\partial }_{\theta }{\tilde{K}}_{x,j}$, and ∣∣⋯∣∣ is the operator norm. Finally then, we obtain our CE-bound in Eq. (21) directly from applying the triangle inequality of the operator norm to the r.h.s. of Eq. (33), which gives36and the minimisation in both Eqs. (33) and (36) is performed independently for each ${\vec{\phi }}_{\ell }$ over all possible single-probe Kraus representations $\tilde{K}(\theta,{\vec{\phi }}_{\ell })$.

As we prove in the Supplementary Note H, whenever the noisy detection channel, ${{{{{{{\mathcal{P}}}}}}}} \sim \, \{p(x|i)\}$ such that ∀_*x*_:*M*_*x*_ = ∑_*x*_*p*(*x*∣*i*)Π_*i*_, is non-trivial, we can always find a Kraus representation such that ${\beta }_{\tilde{K}(\theta,{\vec{\phi }}_{\ell })}=0$ for all *ℓ*. Then, we define the asymptotic CE bound by37which evidently satisfies ${F}_{N}^{({{{{{{{\rm{CE}}}}}}}})}(\{{\vec{\phi }}_{\ell }\})\le {F}_{N}^{({{{{{{{\rm{CE}}}}}}}},{{{{{{{\rm{as}}}}}}}})}(\{{\vec{\phi }}_{\ell }\})$ and ${F}_{N}^{({{{{{{{\rm{CE}}}}}}}})}\mathop{\to}\limits_{N \to \infty} {F}_{N}^{({{{{{{{\rm{CE}}}}}}}},{{{{{{{\rm{as}}}}}}}})}$. Finally, upon optimising ${F}_{N}^{({{{{{{{\rm{CE}}}}}}}},{{{{{{{\rm{as}}}}}}}})}$ further over all local control unitaries gives us38such that ${\bar{{{{{{{{\mathcal{F}}}}}}}}}}_{N}^{({{{{{{{\rm{im}}}}}}}},{{{{{{{\rm{l}}}}}}}})}\le {\bar{F}}_{N}^{({{{{{{{\rm{CE}}}}}}}},{{{{{{{\rm{as}}}}}}}})}$, where39is a constant factor that requires maximisation over $\vec{\phi }$ describing only a single local unitary, and can be proven to be bounded, given the condition ${\beta }_{\tilde{K}(\theta,\vec{\phi })}=0$ is fulfilled.

### Saturating ${\bar{F}}_{N}^{({{{{{{{\rm{CE}}}}}}}},{{{{{{{\rm{as}}}}}}}})}$ with an angular momentum measurement and spin-squeezed states

To obtain the optimal asymptotic CE bound (24), we consider the measurement operators ${\Pi }_{1(2),\vec{\phi }}=|\pm \rangle \langle \pm |$, followed by an asymmetric bit-flip channel ${{{{{{{\mathcal{P}}}}}}}}$ with *p*(1∣1) = ${\mathsf{p}}$, *p*(2∣2) = ${\mathsf{q}}$ for all qubits. As a result, the measurements whose outcomes are actually observed read: ${M}_{1,\vec{\phi }}={\mathsf{p}}{\Pi }_{1,\vec{\phi }}+(1-{\mathsf{q}}){\Pi }_{2,\vec{\phi }}=(1+\delta ){\mathbb{1}}/2+\eta {\sigma }_{{{{{{{{\rm{x}}}}}}}}}/2$, ${M}_{2,\vec{\phi }}=(1-{\mathsf{p}}){\Pi }_{1,\vec{\phi }}+{\mathsf{q}}{\Pi }_{2,\vec{\phi }}=(1-\delta ){\mathbb{1}}/2-\eta {\sigma }_{{{{{{{{\rm{x}}}}}}}}}/2$, where *η* = ${\mathsf{p}}$ + ${\mathsf{q}}$ − 1 and *δ* = ${{\mathsf{p}}-{\mathsf{q}}}$. Constructing a qubit observable taking values ± 1/2 depending on the outcomes *x* = 1 or *x* = 2, it is not hard observe that when measured in parallel on each of the *N* probes and summed, one effectively conducts a measurement of the operator $\hat{O}=N\delta {\mathbb{1}}/2+\eta {\hat{J}}_{{{{{{{{\rm{x}}}}}}}}}$ that constitutes a modification of the total angular momentum ${\hat{J}}_{{{{{{{{\rm{x}}}}}}}}}=\mathop{\sum }\nolimits_{\ell=1}^{N}\frac{{\sigma }_{{{{{{{{\rm{x}}}}}}}}}^{(\ell )}}{2}$, being tailored to the (binary bit-flip) noisy detection channel. A simple estimator of *θ* may then be directly formed by inverting the expectation-value relation $O(\theta )={{{{{{{\rm{Tr}}}}}}}}\{\rho (\theta )\hat{O}\}$ from the outcomes (repeating the protocol *ν* ≫ 1 times).

As derived in the Supplementary Note J, the MSE of such an estimator, given sufficiently large number *ν* of measurement repetitions, is well approximated by the (generalised) error-propagation formula,

40 $$\nu {\Delta }^{2}{\tilde{\theta }}_{N}=\frac{{\Delta }^{2}{\hat{J}}_{{{{{{{{\rm{x}}}}}}}}}}{{\left|{\partial }_{\theta }\langle {\hat{J}}_{{{{{{{{\rm{x}}}}}}}}}\rangle \right|}^{2}}-\frac{\delta \langle {\hat{J}}_{{{{{{{{\rm{x}}}}}}}}}\rangle }{\eta {\left|{\partial }_{\theta }\langle {\hat{J}}_{{{{{{{{\rm{x}}}}}}}}}\rangle \right|}^{2}}+\frac{N}{4{\eta }^{2}}\frac{1-{\eta }^{2}-{\delta }^{2}}{{\left|{\partial }_{\theta }\langle {\hat{J}}_{{{{{{{{\rm{x}}}}}}}}}\rangle \right|}^{2}},$$

where $$\langle \ldots \,\rangle={{{{{{{\rm{Tr}}}}}}}}\{{\rho }^{N}(\theta )\ldots \,\}$$ and $${\Delta }^{2}{\hat{J}}_{{{{{{{{\rm{x}}}}}}}}}=\langle {\hat{J}}_{{{{{{{{\rm{x}}}}}}}}}^{2}\rangle -{\langle {\hat{J}}_{{{{{{{{\rm{x}}}}}}}}}\rangle }^{2}$$.

Consider now ${\rho }^{N}=\left|\phi,\mu \right\rangle \left\langle \phi,\mu \right|$ with $\left|\phi,\mu \right\rangle={{{{{{{{\rm{e}}}}}}}}}^{{{{{{{{\rm{i}}}}}}}}\phi {\hat{J}}_{{{{{{{{\rm{z}}}}}}}}}}\left|\mu \right\rangle$ and

41 $$\left | \mu \right\rangle={W}_{\mu }{{{{{{{{\rm{e}}}}}}}}}^{-{{{{{{{\rm{i}}}}}}}}{\Theta }_{\mu }{W}_{\mu }^{{{{\dagger}}} }{\hat{J}}_{{{{{{{{\rm{y}}}}}}}}}{W}_{\mu }}{|j,{m}_{y}=j\rangle }_{{{{{{{{\rm{y}}}}}}}}}$$

being the one-axis spin-squeezed state^57^ expressed in the angular momentum eigenbasis defined by the ${\hat{J}}^{2}$ and ${\hat{J}}_{{{{{{{{\rm{y}}}}}}}}}$ operators, where ${W}_{\mu }={{{{{{{{\rm{e}}}}}}}}}^{-{{{{{{{\rm{i}}}}}}}}\mu {\hat{J}}_{{{{{{{{\rm{z}}}}}}}}}^{2}/2}$ is the unitary squeezing operation of strength *μ*, while Θ_*μ*_ = *π*/2−*ϵ* with $\epsilon=\arctan (b/a)$, $a=1-{\cos }^{2j-2}\mu$ and $b=4\sin (\mu /2){\cos }^{2j-2}(\mu /2)$. For our purpose we will consider states (41) obtained by squeezing a completely polarised ensemble spins along the *y*-axis, i.e., prepared in a state ${|j,{m}_{y}=j\rangle }_{{{{{{{{\rm{y}}}}}}}}}$ with *j* = *m*_*j*_ = *N*/2. Substituting such choice into the error-propagation expression (40), we arrive after lengthy but straightforward algebra at

42 $$\nu {\Delta }^{2}{\tilde{\theta }}_{N}=	\frac{{\cos }^{2}\varphi {\left({\Delta }^{2}{\hat{J}}_{{{{{{{{\rm{x}}}}}}}}}\right)}_{\mu }+{\sin }^{2}\varphi {\left({\Delta }^{2}{\hat{J}}_{{{{{{{{\rm{y}}}}}}}}}\right)}_{\mu }}{{\cos }^{2}\varphi \,{\langle {\hat{J}}_{{{{{{{{\rm{y}}}}}}}}}\rangle }_{\mu }^{2}}\\ 	-\frac{\delta \sin \varphi {\langle {\hat{J}}_{{{{{{{{\rm{y}}}}}}}}}\rangle }_{\mu }}{\eta {\cos }^{2}\varphi {\langle {\hat{J}}_{{{{{{{{\rm{y}}}}}}}}}\rangle }_{\mu }^{2}}+\frac{N}{4{\eta }^{2}}\frac{1-{\eta }^{2}-{\delta }^{2}}{{\cos }^{2}\varphi {\langle {\hat{J}}_{{{{{{{{\rm{y}}}}}}}}}\rangle }_{\mu }^{2}},$$

where the subscripts *μ* indicate expectations to be evaluated w.r.t. the state $\left|\mu \right\rangle$ in Eq. (41), having defined *φ* ≔ *ϕ* + *θ* as in Eq. (9). For large *N*, we find that after choosing the squeezing strength to scale as *μ* ∼ *N* ^−8/9^, one has ${({\Delta }^{2}{\hat{J}}_{{{{{{{{\rm{x}}}}}}}}})}_{\mu } \sim \, {N}^{7/9},{({\Delta }^{2}{\hat{J}}_{{{{{{{{\rm{y}}}}}}}}})}_{\mu } \sim \, {N}^{4/9}/128,{\langle \, {\hat{J}}_{{{{{{{{\rm{y}}}}}}}}}\rangle }_{\mu } \sim \, N/2$, and therefore:

43 $$\nu {\Delta }^{2}{\tilde{\theta }}_{N} \sim \, \frac{1}{N}\frac{1}{{\cos }^{2}\varphi }\left(\frac{1-{\delta }^{2}-{\eta }^{2}}{{\eta }^{2}}-\frac{2\delta }{\eta }\sin \varphi \right).$$

Finally, by choosing now *φ* = *φ*_opt_ as the angle derived in single-probe (qubit) setting in Eq. (12), the r.h.s. of Eq. (43) converges exactly to $1/{\bar{F}}_{N}^{({{{{{{{\rm{CE}}}}}}}},{{{{{{{\rm{as}}}}}}}})}$ with ${\bar{F}}_{N}^{({{{{{{{\rm{CE}}}}}}}},{{{{{{{\rm{as}}}}}}}})}$ stated in Eq. (24). In other words, the asymptotic ultimate precision is achieved by (imperfectly) measuring the total angular momentum in the x-direction, while preparing the *N* probes (spin-1/2s) in a spin-squeezed state rotated by the same optimal angle (12) as in the single-probe scenario, with squeezing parameter scaling as *μ* ∼ *N*^−8/9^ with *N*.

### Application to lossy photonic interferometry with dark counts

**Fig. 8:**
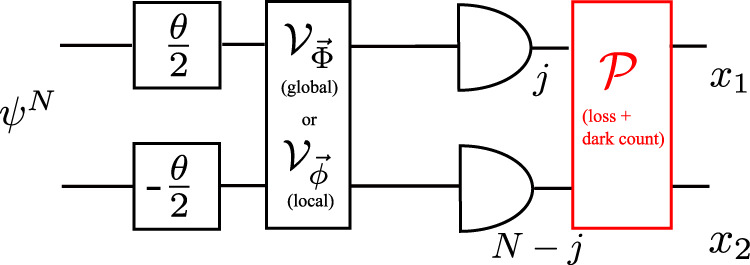
Sensing the relative phase *θ* with a pure *N*-photon state, *ψ*^*N*^, in a lossy interferometer with dark counts. Either a global (affects all the photons) or local (affects each photon separately) control unitary is allowed. The imperfect measurement is described by an ideal photon-number detection (*j*, *N*−*j*) at the output modes, followed by a noisy detection channel ${{{{{{{\mathcal{P}}}}}}}}$ that incorporates losses and dark counts, resulting in (*x*_1_, *x*_2_) registered detection counts.

In this section, we consider another standard problem in quantum metrology, namely, two-mode interferometry involving *N*-photon quantum states of light. The effective full Hilbert space is thus spanned by the Fock basis ${\{|j,N-j \rangle \}}_{j=0}^{N}$. The detection channel is two identical photodetectors measuring each mode, which independently suffer from losses and dark counts. Specifically, we quantify the losses by efficiency *η*, i.e., a photon is detected (or lost) with probability *η* (or 1−*η*)^80^. Moreover, we assume that each photodetector may experience a single dark count with probability p per each photon that enters the interferometer—in case the *N* photons arrive in distinct time-bins, this corresponds to observing at most one dark count per time-bin. Note that this results in extending the effective measured Hilbert space to $\{|{x}_{1},{x}_{2}\rangle \}$ with 0≤*x*_1_, *x*_2_≤2*N* and 0≤*x*_1_ + *x*_2_≤3*N*. A schematic of the problem is depicted in Fig. 8.

Given the above imperfect measurement model, let us find the corresponding ${\gamma }_{{{{{{{{{\mathcal{M}}}}}}}}}^{\otimes N}}$ in Eq. (7) that effectively determines the imperfect (channel) QFI (6), while requiring a global control unitary to be performed, ${{{{{{{{\mathcal{V}}}}}}}}}_{\vec{\Phi }}$ in Fig. 8. Note that, the photon-counting measurement is local w.r.t. the Hilbert spaces associated with each photon, while the global control may now in principle require *N*-photon interactions, i.e., may be highly non-linear within the second quantisation^81^. Firstly, we ignore the dark counts, so that 0≤*x*_1_ + *x*_2_≤*N* and

44 $${\gamma }_{{{{{{{{{\mathcal{M}}}}}}}}}^{\otimes N}}	=\mathop{\max }\limits_{\left|\xi \right\rangle,\left|{\xi }_{\perp }\right\rangle }\mathop{\sum}\limits_{{x}_{1},{x}_{2}}\frac{{{{{{{{\rm{Re}}}}}}}}{\left\{\langle {\xi }_{\perp }|{M}_{{x}_{1},{x}_{2}}|\xi \rangle \right\}}^{2}}{\langle \xi|{M}_{{x}_{1},{x}_{2}}|\xi \rangle }\\ 	=\mathop{\max }\limits_{{{{{{{{\bf{a}}}}}}}}\cdot {{{{{{{\bf{b}}}}}}}}={{{{{{{\bf{0}}}}}}}},{\left|{{{{{{{\bf{a}}}}}}}}\right|}^{2}={\left|{{{{{{{\bf{b}}}}}}}}\right|}^{2}=1}\mathop{\sum}\limits_{{x}_{1},{x}_{2}}\frac{{\left({\sum }_{j}p\left({x}_{1},{x}_{2}|j,N-j\right){a}_{j}{b}_{j}\right)}^{2}}{{\sum }_{j}p\left({x}_{1},{x}_{2}|j,N-j\right){a}_{j}^{2}},$$

where $|\xi \rangle=\mathop{\sum }\nolimits_{j=0}^{N}{a}_{j}|j,N-j\rangle$, $|{\xi }_{\perp } \rangle=\mathop{\sum }\nolimits_{j=0}^{N}{b}_{j}|j,N-j \rangle$ are some orthogonal vectors with real coefficients. The imperfect photon-count measurement corresponds then to the POVM: ${{{{{{{{\mathcal{M}}}}}}}}}^{\otimes N} \sim \{{M}_{{x}_{1},{x}_{2}}=\mathop{\sum }\nolimits_{j=0}^{N}p({x}_{1},{x}_{2}|j,N-j)|j \rangle \langle j |\otimes |N-j \rangle \langle N-j |\}$, whose mixing coefficients are defined by the noisy detection channel responsible for *photon loss*, ${{{{{{{{\mathcal{P}}}}}}}}}_{{{{{{{{\rm{loss}}}}}}}}} \sim \{p\left({x}_{1},{x}_{2}|j,N-j\right)\}$ with

45 $$\begin{array}{l}p\left({x}_{1},{x}_{2}|j,N-j\right)={p}_{\eta }\left({x}_{1}|j\right){p}_{\eta }\left({x}_{2}|N-j\right)\\=\left(\begin{array}{c}j\\ {x}_{1}\end{array}\right)\left(\begin{array}{c}N-j\\ {x}_{2}\end{array}\right){\eta }^{{x}_{1}+{x}_{2}}{\left(1-\eta \right)}^{N-{x}_{1}-{x}_{2}},\end{array}$$

where  is a binomial distribution arising due to the finite detection efficiency *η*.

We observe from numerical simulations that

46 $$\begin{array}{rcl}\left|\xi \right\rangle &=&\sin \varphi \left|N,0\right\rangle+\cos \varphi \left|0,N\right\rangle \\ \left|{\xi }_{\perp }\right\rangle &=&\cos \varphi \left|N,0\right\rangle -\sin \varphi \left|0,N\right\rangle \end{array}$$

are optimal for any $\varphi \in {\mathbb{R}}$. Assuming the form of $|\xi \rangle$ and $|{\xi }_{\perp } rangle$ as above, we can calculate ${\gamma }_{{{{{{{{{\mathcal{M}}}}}}}}}^{\otimes N}}$ explicitly in Eq. (44):

47 $$\begin{array}{rcl}{\gamma }_{{{{{{{{{\mathcal{M}}}}}}}}}^{\otimes N}}&=&\mathop{\sum}\limits_{{x}_{1}\ne 0}\frac{{\left({p}_{\eta }\left({x}_{1}|N\right)\sin \varphi \cos \varphi \right)}^{2}}{{p}_{\eta }\left({x}_{1}|N\right){\sin }^{2}\varphi }+\\ &&\mathop{\sum}\limits_{{x}_{2}\ne 0}\frac{{\left({p}_{\eta }\left({x}_{2}|N\right)\sin \varphi \cos \varphi \right)}^{2}}{{p}_{\eta }\left({x}_{2}|N\right){\cos }^{2}\varphi }\\ &=&\mathop{\sum}\limits_{x\ne 0}{p}_{\eta }\left(x|N\right)=1-{\left(1-\eta \right)}^{N}.\end{array}$$

The above expression has a simple interpretation: for *φ* = 0 the states $|\xi \rangle=|0,N \rangle$, $\left|{\xi }_{\perp }\right\rangle=\left|N,0\right\rangle$ remain orthogonal unless all photons are lost in both arms, what may happen only with probability ${\left(1-\eta \right)}^{N}$. Moreover, this expression coincides with the lower bound on ${\gamma }_{{{{{{{{{\mathcal{M}}}}}}}}}^{\otimes N}}$ used in Eq. (17) i.e., $1-{\sum }_{{{{{{{{\boldsymbol{x}}}}}}}}}\sqrt{{p}_{+}({{{{{{{\boldsymbol{x}}}}}}}}){p}_{-}({{{{{{{\boldsymbol{x}}}}}}}})}$ in Eq. (57) of the Supplementary Note E; this is due to the fact that for every ***x*** either *p*_+_(***x***)*p*_−_(***x***) = 0 or *p*_+_(***x***) = *p*_−_(***x***). Finally, we observe that by lifting the assumption of photodetection efficiency being equal in both arms, no longer *φ* may be arbitrary chosen in Eq. (46), but rather must also be optimised.

As a result, considering e.g., the N00N state as the the input probe *ψ*^*N*^ for which ${{{{{{{\mathcal{F}}}}}}}}[{\psi }^{N}(\theta )]={N}^{2}$, we obtain the equivalent of Eq. (17) in the form

48 $${F}_{N}({V}_{\vec{\Phi }})={N}^{2}\left[1-{\left(1-\eta \right)}^{N}\right]={N}^{2}[1-{{{{{{{{\rm{e}}}}}}}}}^{-\chi N}]$$

with $\chi=-\ln (1-\eta )$ to be compared with the one in Eq. (20). As anticipated, the HS is maintained despite the imperfect measurement, however, the necessary global control unitary, ${{{{{{{{\mathcal{V}}}}}}}}}_{\vec{\Phi }}$ in Fig. 8, must rotate the encoded state $|{\psi }^{N}(\theta ) \rangle=({{{{{{{{\rm{e}}}}}}}}}^{{{{{{{{\rm{i}}}}}}}}N\theta /2}|N,0 \rangle+{{{{{{{{\rm{e}}}}}}}}}^{-{{{{{{{\rm{i}}}}}}}}N\theta /2} |0,N \rangle )/\sqrt{2}$ and its orthogonal $|{\psi }_{\perp }^{N}(\theta ) \rangle={{{{{{{\rm{i}}}}}}}}({{{{{{{{\rm{e}}}}}}}}}^{{{{{{{{\rm{i}}}}}}}}N\theta /2}|N,0 \rangle -{{{{{{{{\rm{e}}}}}}}}}^{-{{{{{{{\rm{i}}}}}}}}N\theta /2}|0,N \rangle )/\sqrt{2}$ onto the optimal $\left|\xi \right\rangle$ and $|{\xi }_{\perp } \rangle$ in Eq. (46). Note that it is a highly non-linear operation allowing to “disentangle” N00N states. For instance, for *φ*, *θ* = 0 it rotates the input N00N state and its perpendicular component onto the desired $|0,N \rangle$ and $|N,0 \rangle$, respectively, which are product w.r.t. the Hilbert spaces associated with each photon, ${{\mathbb{C}}}_{2}^{\otimes N}$.

Consider now also the presence of dark counts parametrised by the rate ${\mathsf{p}}$; as before, this corresponds to observing at most one dark count per time-bin when the *N* photons arrive in distinct time-bins. Similarly then to ${{{{{{{{\mathcal{P}}}}}}}}}_{{{{{{{{\rm{loss}}}}}}}}}$, the detection channel responsible for *dark counts* is characterised by a binomial distribution  so that ${{{{{{{{\mathcal{P}}}}}}}}}_{{{{{{{{\rm{dc}}}}}}}}} \sim \, \{p({y}_{1},{y}_{2}|N)\}$ with

49 $$p\left({y}_{1},{y}_{2}|N\right)={p}_{{\mathsf{p}}}\left({y}_{1}|N\right){\mathsf{p}}_{{\mathsf{p}}}\left({y}_{2}|N\right),$$

where *y*_1_ and *y*_2_ are respectively the number of dark counts in each detector. The resultant overall noisy detection channel corresponds to the composition of ${{{{{{{{\mathcal{P}}}}}}}}}_{{{{{{{{\rm{loss}}}}}}}}}$ and ${{{{{{{{\mathcal{P}}}}}}}}}_{{{{{{{{\rm{dc}}}}}}}}}$, i.e., ${{{{{{{\mathcal{P}}}}}}}} \sim \, \{p ({x}_{1},{x}_{2}|j,N-j )\}$ with elements

50 $$p\left({x}_{1},{x}_{2}|j,N-j\right)={p}_{\eta,{\mathsf{p}}}\left({x}_{1}|j\right){p}_{\eta,{\mathsf{p}}}\left({x}_{2}|N-j\right),$$

where ${p}_{\eta,{{{\rm{p}}}}} (x|k )=\mathop{\sum }\nolimits_{m=0}^{x}{p}_{\eta } (m|k ){\mathsf{p}}_{{\mathsf{p}}} (x-m|N )$, while *x*_1_ and *x*_2_ are respectively the final number of photons registered in each of the photodetectors.

Verifying numerically again that optimal $|\xi \rangle$, $\left|{\xi }_{\perp }\right\rangle$ take the form (46), we find ${\gamma }_{{{{{{{{{\mathcal{M}}}}}}}}}^{\otimes N}}$ in Eq. (44) to read

51 $$\begin{array}{c}{\gamma }_{{{{{{{{{\mathcal{M}}}}}}}}}^{\otimes N}}=\mathop{\sum}\limits_{{x}_{1},{x}_{2}}{\left(\sin \varphi \cos \varphi \right)}^{2}\times \\ \frac{{\left({p}_{\eta,\mathsf p}\left({x}_{1}|N\right){\mathsf{p}}_{\eta,\mathsf p}\left({x}_{2}|0\right)-{p}_{\eta,\mathsf p}\left({x}_{1}|0\right){\mathsf{p}}_{\eta,\mathsf p}\left({x}_{2}|N\right)\right)}^{2}}{{\sin }^{2}\varphi \,{p}_{\eta,\mathsf p}\left({x}_{1}|N\right){\mathsf{p}}_{\eta,\mathsf p}\left({x}_{2}|0\right)+{\cos }^{2}\varphi \,{p}_{\eta,\mathsf p}\left({x}_{1}|0\right){\mathsf{p}}_{\eta,\mathsf p}\left({x}_{2}|N\right)}.\end{array}$$

**Fig. 9:**
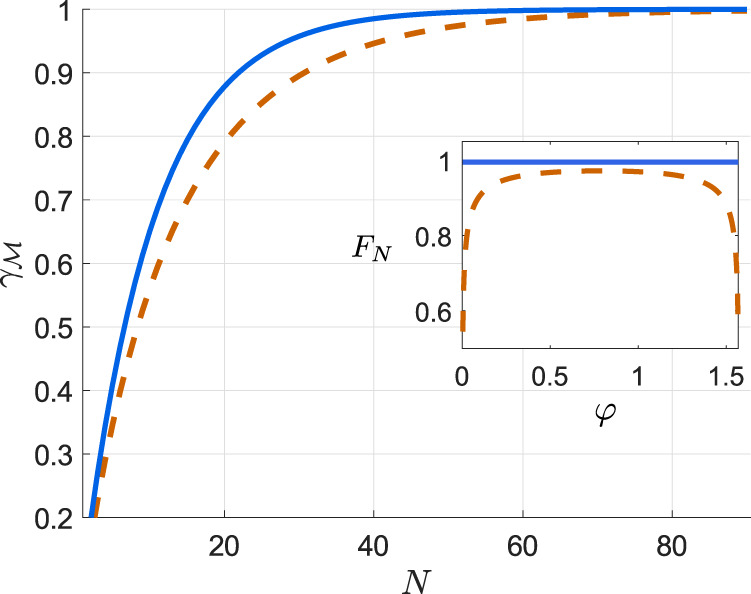
Convergence to the perfect QFI with the photon-number *N* for different noisy photodetection channels. ${\gamma }_{{{{{{{{{\mathcal{M}}}}}}}}}^{\otimes N}}$ for different dark-count rates, ${\mathsf{p}}$, given a finite detection efficiency (losses) *η* = 0.1. The blue (solid) line corresponds to losses without dark counts ${\mathsf{p}}$ = 0, while the orange dashed line also includes a dark-count rate of ${\mathsf{p}}=0.01$. *Inset:* The FI as a function of the angle *φ* between $|N,0 \rangle$ and $|0,N \rangle$ in Eq. (46) that parametrises the global control unitary ${V}_{\vec{\Phi }}$. While with only losses (solid blue line) any angle is optimal, dark counts (dashed orange line) remove this symmetry. In this illustration, *N* = 50.

The dark counts lift the degeneracy in *φ* and reduce ${\gamma }_{{{{{{{{{\mathcal{M}}}}}}}}}^{\otimes N}}$. A comparison between ${\gamma }_{{{{{{{{{\mathcal{M}}}}}}}}}^{\otimes N}}$ evaluated numerically for different levels of dark-counts rate, ${\mathsf{p}}$, is presented in Fig. 9. Importantly, ${\gamma }_{{{{{{{{{\mathcal{M}}}}}}}}}^{\otimes N}}$ still approaches unity as *N* increases, however, the optimal control unitary ${{{{{{{{\mathcal{V}}}}}}}}}_{\vec{\Phi }}$ is more involved and the convergence is slower. Finding an analytical expression for the convergence rate with dark counts, *χ* as in Eq. (48), we leave as an open challenge.

We now turn to the scenario when only local control unitary operations are allowed, i.e., ones that may affect only a single (two-mode) photon, denoted by ${{{{{{{{\mathcal{V}}}}}}}}}_{\vec{\phi }}$ in Fig. 8. For this, let us imagine a more advanced interferometry scheme in which the input *N* photons can be resolved into different time-bins, despite all of them occupying a bosonic (permutation invariant) state^82^. Within such a picture, each time-bin is represented by a qubit with basis states $\left|0\right\rangle \equiv \left|1,0\right\rangle$, $\left|1\right\rangle \equiv \left|0,1\right\rangle$ corresponding to a single photon occupying either of the two optical modes. Moreover, the ideal measurement in each time-bin is then described by projectors ({Π_1(2)_}) onto the above basis states, each yielding a “click” in either of the detectors.

We include the loss and dark-count noise within the detection process, as described by Eq. (50), but the rate of the latter, p, to be small enough, so that at most one false detection event may occur per time-bin (photon). Consequently, the noise leads to six possible ‘observable’ outcomes (i.e., *d* = 2→∣*X*∣ = 6), namely, $\left({2}\atop{0}\right),\left({0}\atop{2}\right),\left({1}\atop{1}\right),\left({0}\atop{0}\right),\left({1}\atop{0}\right)$, and $\left({0}\atop{1}\right)$, which we will respectively label as outcomes *x* = 1 to 6—by $\left({{x}_{1}}\atop{{x}_{2}}\right)$ we denote that *x*_1_ (*x*_2_) “clicks” were recorded in the upper (lower) detector. The resulting imperfect measurement ${{{{{{{\mathcal{M}}}}}}}}$ performed in each time-bin is then specified by ${M}_{x}=\mathop{\sum }\nolimits_{i=1}^{2}p(x|i){\Pi }_{i}$, where *p*(*x*∣*i*) is the (*x*, *i*)-th entry of the stochastic matrix:

52 $${{{{{{{{\mathcal{P}}}}}}}}}^{(1)}=\left(\begin{array}{cc}{\mathsf{p}}\eta &0\\ 0&{\mathsf{p}}\eta \\ {\mathsf{p}}\eta &{\mathsf{p}}\eta \\ 1-2{\mathsf{p}}-\eta+2{\mathsf{p}}\eta &1-2{\mathsf{p}}-\eta+2{\mathsf{p}}\eta \\ {\mathsf{p}}+\eta -3{\mathsf{p}}\eta &{\mathsf{p}}-{\mathsf{p}}\eta \\ {\mathsf{p}}-{\mathsf{p}}\eta &{\mathsf{p}}+\eta -3{\mathsf{p}}\eta \end{array}\right),$$

which can be obtained equivalently by evaluating the form of detection channel ${{{{{{{\mathcal{P}}}}}}}}$ defined in Eq. (50) for *N* = 1, and truncating the quadratic terms in p.

Possessing the form of the local (single-photon) detection noise (52), we follow our technique based on the CE-method^40,42,69^ to compute upper bounds on the precision in estimating *θ*, where thanks to employing the quantum-classical channel formalism we are able explicitly determine the asymptotic CE-bound, as defined in Eqs. (23) and (38), despite ∣*X*∣≠*d*, i.e., the number of ‘observable’ outcomes differing from the ‘inaccessible’ ones, i.e.,:

53 $${F}_{N}^{({{{{{{{\rm{CE}}}}}}}},{{{{{{{\rm{as}}}}}}}})}=N\frac{\eta (\eta -3{\mathsf{p}}\eta+2{{\mathsf{p}}}^{2})}{2{\mathsf{p}}+{\eta }^{2}(3{\mathsf{p}}-1)+\eta (1-4{\mathsf{p}}-2{{\mathsf{p}}}^{2})},$$

whereas the finite CE-bound, ${F}_{N}^{({{{{{{{\rm{CE}}}}}}}})}$ in Eqs. (21) and (36), can be computed efficiently via a semi-definite programme (SDP). We leave an explicit proof open, however, the derivation of Eq. (38) suggests the asymptotic CE-bound (53) to apply also to protocols involving (local) adaptive measurements^82^.

**Fig. 10:**
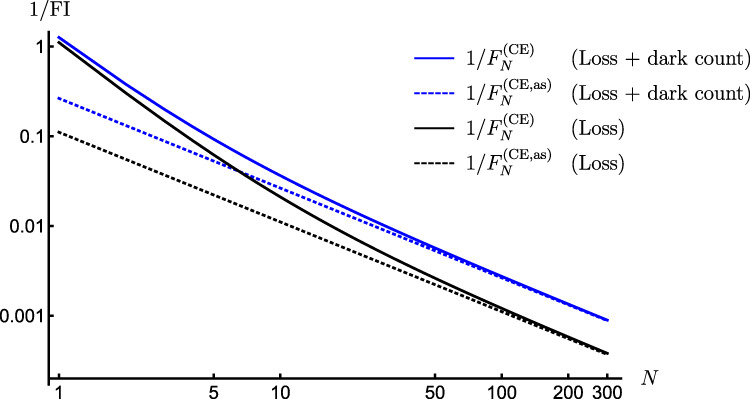
Ultimate bounds on precision in lossy photonic interferometry with dark counts and local unitary control. The solid and dotted blue lines are the inverse of the finite-*N* (evaluated via an SDP) and asymptotic (see Eq. (53)) CE bounds, respectively, for detection efficiency *η* = 0.9 and dark-count rate ${\mathsf{p}}=0.1$. Control gates may act on each individual dual-rail photon. For comparison, we also plot, solid and dotted black lines, the corresponding quantities when only photon loss is present (*η* = 0.9, ${\mathsf{p}}$ = 0).

For illustration, in Fig. 10 we plot in blue the respective inverses of ${F}_{N}^{({{{{{{{\rm{CE}}}}}}}})}$ and ${F}_{N}^{({{{{{{{\rm{CE}}}}}}}},{{{{{{{\rm{as}}}}}}}})}$ for ${\mathsf{p}}=0.1$ and *η* = 0.9. Note that, as it should, the presence of dark counts worsen the estimation precision as compared to the having just lossy detectors, which can be obtained by taking ${\mathsf{p}} \rightarrow 0$, and are plotted in black. As a side note, in the latter case, ${F}_{N}^{({{{{{{{\rm{CE}}}}}}}})}$ and ${F}_{N}^{({{{{{{{\rm{CE}}}}}}}},{{{{{{{\rm{as}}}}}}}})}=\eta /(1-\eta )$ have also been obtained by expanding the space to a qutrit state, where an auxiliary mode 3 is introduced to keep track of the $\left({{0}\atop {0}}\right)$ outcome^69^. Meanwhile, on the other extreme, when the detector has unity efficiency but only just dark count, we have ${F}_{N}^{({{{{{{{\rm{CE}}}}}}}},{{{{{{{\rm{as}}}}}}}})}=N({{\mathsf{p}}}^{-1}-1)$. Interestingly, we observe that the CE-bound for the effect of pure loss with rate 1−*η* is the same as that of a pure dark count with rate ${\mathsf{p}}=1-\eta$, true both for finite-*N* and asymptotically.

## Supplementary information

Supplementary Information

## Data Availability

All data relevant to this study are available from the corresponding authors upon request.

## Code availability

The code used for simulations is available from the corresponding authors upon request.

## References

[1] Degen CL, Reinhard F, Cappellaro P (2017). Quantum sensing. Rev. Mod. Phys..

[2] Awschalom DD, Hanson R, Wrachtrup J, Zhou BB (2018). Quantum technologies with optically interfaced solid-state spins. Nat. Photonics.

[3] Barry JF (2020). Sensitivity optimization for nv-diamond magnetometry. Rev. Mod. Phys..

[4] Pezzè L, Smerzi A, Oberthaler MK, Schmied R, Treutlein P (2018). Quantum metrology with nonclassical states of atomic ensembles. Rev. Mod. Phys..

[5] Bongs K (2019). Taking atom interferometric quantum sensors from the laboratory to real-world applications. Nat. Rev. Phys..

[6] Tse M (2019). Quantum-enhanced advanced ligo detectors in the era of gravitational-wave astronomy. Phys. Rev. Lett..

[7] Giovannetti V, Lloyd S, Maccone L (2004). Quantum-enhanced measurements: Beating the Standard Quantum Limit. Science.

[8] Leibfried D (2004). Toward Heisenberg-limited spectroscopy with multiparticle entangled states. Science.

[9] Mitchell MW, Lundeen JS, Steinberg AM (2004). Super-resolving phase measurements with a multi-photon entangled state. Nature.

[10] Estève J, Gross C, Weller A, Giovanazzi S, Oberthale MK (2008). Squeezing and entanglement in a bose-einstein condensate. Nature.

[11] Appel J (2009). Mesoscopic atomic entanglement for precision measurements beyond the standard quantum limit. Proc. Natl. Acad. Sci. U.S.A..

[12] Sewell R (2012). Magnetic sensitivity beyond the projection noise limit by spin squeezing. Phys. Rev. Lett..

[13] Hosten O, Engelsen NJ, Krishnakumar R, Kasevich MA (2016). Measurement noise 100 times lower than the quantum-projection limit using entangled atoms. Nature.

[14] Helstrom, C. W. Quantum Detection and Estimation Theory (Academic Press, 1976).

[15] Holevo, A. S.Probabilistic and Statistical Aspects of Quantum Theory (North Holland, 1982).

[16] Braunstein SL, Caves CM (1994). Statistical distance and the geometry of quantum states. Phys. Rev. Lett..

[17] Kay, S. M. Fundamentals of Statistical Signal Processing: Estimation Theory (Prentice Hall, 1993).

[18] Giovannetti V, Lloyd S, Maccone L (2006). Quantum metrology. Phys. Rev. Lett..

[19] Hammerer K, Sørensen AS, Polzik ES (2010). Quantum interface between light and atomic ensembles. Rev. Mod. Phys..

[20] Clerk AA, Devoret MH, Girvin SM, Marquardt F, Schoelkopf RJ (2010). Introduction to quantum noise, measurement, and amplification. Rev. Mod. Phys..

[21] Batalov A (2008). Temporal coherence of photons emitted by single nitrogen-vacancy defect centers in diamond using optical rabi-oscillations. Phys. Rev. Lett..

[22] Jelezko F, Wrachtrup J (2006). Single defect centres in diamond: A review. Phys. Status Solidi A.

[23] Schirhagl R, Chang K, Loretz M, Degen CL (2014). Nitrogen-vacancy centers in diamond: nanoscale sensors for physics and biology. Annu. Rev. Phys. Chem.

[24] Sete EA, Martinis JM, Korotkov AN (2015). Quantum theory of a bandpass purcell filter for qubit readout. Phys. Rev. A.

[25] Heinsoo J (2018). Rapid high-fidelity multiplexed readout of superconducting qubits. Phys. Rev. Appl..

[26] Krantz P (2019). A quantum engineer’s guide to superconducting qubits. Appl. Phys. Rev..

[27] Bergquist J, Hulet RG, Itano WM, Wineland D (1986). Observation of quantum jumps in a single atom. Phys. Rev. Lett..

[28] Nagourney W, Sandberg J, Dehmelt H (1986). Shelved optical electron amplifier: Observation of quantum jumps. Phys. Rev. Lett..

[29] Sauter T, Neuhauser W, Blatt R, Toschek PE (1986). Observation of quantum jumps. Phys. Rev. Lett..

[30] Myerson AH (2008). High-fidelity readout of trapped-ion qubits. Phys. Rev. Lett..

[31] Marciniak CD (2022). Optimal metrology with variational quantum circuits on trapped ions. Nature.

[32] Harris J, Boyd RW, Lundeen JS (2017). Weak value amplification can outperform conventional measurement in the presence of detector saturation. Phys. Rev. Lett..

[33] Xu L (2020). Approaching quantum-limited metrology with imperfect detectors by using weak-value amplification. Phys. Rev. Lett..

[34] Davis E, Bentsen G, Schleier-Smith M (2016). Approaching the heisenberg limit without single-particle detection. Phys. Rev. Lett..

[35] Fröwis F, Sekatski P, Dür W (2016). Detecting large quantum fisher information with finite measurement precision. Phys. Rev. Lett..

[36] Nolan SP, Szigeti SS, Haine SA (2017). Optimal and robust quantum metrology using interaction-based readouts. Phys. Rev. Lett..

[37] Haine SA (2018). Using interaction-based readouts to approach the ultimate limit of detection-noise robustness for quantum-enhanced metrology in collective spin systems. Phys. Rev. A.

[38] Linnemann D (2016). Quantum-enhanced sensing based on time reversal of nonlinear dynamics. Phys. Rev. Lett..

[39] Maccone L, Giovannetti V (2011). Quantum metrology: Beauty and the noisy beast. Nat. Phys..

[40] Fujiwara A, Imai H (2008). A fibre bundle over manifolds of quantum channels and its application to quantum statistics. J. Phys. A: Math. Theor..

[41] Escher BM, de Matos Filho RL, Davidovich L (2011). General framework for estimating the ultimate precision limit in noisy quantum-enhanced metrology. Nat. Phys..

[42] Demkowicz-Dobrzański R, Kołodyński J, Guţă M (2012). The elusive Heisenberg limit in quantum-enhanced metrology. Nat. Commun..

[43] Dür W, Skotiniotis M, Fröwis F, Kraus B (2014). Improved quantum metrology using quantum error correction. Phys. Rev. Lett..

[44] Arrad G, Vinkler Y, Aharonov D, Retzker A (2014). Increasing sensing resolution with error correction. Phys. Rev. Lett..

[45] Sekatski P, Skotiniotis M, Kołodyński J, Dür W (2017). Quantum metrology with full and fast quantum control. Quantum.

[46] Demkowicz-Dobrzański R, Czajkowski J, Sekatski P (2017). Adaptive Quantum Metrology Under General Markovian noise. Phys. Rev. X.

[47] Zhou S, Zhang M, Preskill J, Jiang L (2018). Achieving the Heisenberg limit in quantum metrology using quantum error correction. Nat. Commun..

[48] Maciejewski FB, Zimborás Z, Oszmaniec M (2020). Mitigation of readout noise in near-term quantum devices by classical post-processing based on detector tomography. Quantum.

[49] Bravyi S, Sheldon S, Kandala A, Mckay DC, Gambetta JM (2021). Mitigating measurement errors in multiqubit experiments. Phys. Rev. A.

[50] Maze JR (2008). Nanoscale magnetic sensing with an individual electronic spin in diamond. Nature.

[51] Taylor JM (2008). High-sensitivity diamond magnetometer with nanoscale resolution. Nat. Phys..

[52] Jiang L (2009). Repetitive readout of a single electronic spin via quantum logic with nuclear spin ancillae. Science.

[53] Neumann P (2010). Single-shot readout of a single nuclear spin. Science.

[54] Holevo AS (1998). Quantum coding theorems. Russ. Math. Surv..

[55] Greenberger, D. M., Horne, M. & Zeilinger, A. Going beyond bell’s theorem. In Kafatos, M. (ed.) *Bell’s Theorem, Quantum Theory, and Conceptions of the Universe*, vol. 37 of *Fundamental Theories of Physics*, 69–72 (Springer Netherlands, 1989).

[56] Wineland DJ, Bollinger JJ, Itano WM, Moore FL, Heinzen DJ (1992). Spin squeezing and reduced quantum noise in spectroscopy. Phys. Rev. A.

[57] Kitagawa M, Ueda M (1993). Squeezed spin states. Phys. Rev. A.

[58] Alipour S, Rezakhani AT (2015). Extended convexity of quantum fisher information in quantum metrology. Phys. Rev. A.

[59] Pang S, Brun TA (2014). Quantum metrology for a general Hamiltonian parameter. Phys. Rev. A.

[60] Mirkhalaf SS, Benedicto Orenes D, Mitchell MW, Witkowska E (2021). Criticality-enhanced quantum sensing in ferromagnetic bose-einstein condensates: Role of readout measurement and detection noise. Phys. Rev. A.

[61] Doherty MW (2013). The nitrogen-vacancy colour centre in diamond. Phys. Rep..

[62] Rondin L (2014). Magnetometry with nitrogen-vacancy defects in diamond. Rep. Prog. Phys..

[63] Cover, T. & Thomas, J. Elements of information theory (John Wiley and Sons, 1991).

[64] Jarrett RG (1984). Bounds and expansions for Fisher information when the moments are known. Biometrika.

[65] Smirne A, Kołodyński J, Huelga SF, Demkowicz-Dobrzański R (2016). Ultimate precision limits for noisy frequency estimation. Phys. Rev. Lett..

[66] Audenaert KMR (2007). Discriminating states: the quantum Chernoff bound. Phys. Rev. Lett..

[67] Calsamiglia J, Muñoz-Tapia R, Masanes L, Acin A, Bagan E (2008). Quantum Chernoff bound as a measure of distinguishability between density matrices: Application to qubit and Gaussian states. Phys. Rev. A.

[68] Audenaert KMR, Nussbaum M, Szkoła A, Verstraete F (2008). Asymptotic error rates in quantum hypothesis testing. Commun. Math. Phys..

[69] Kołodyński J, Demkowicz-Dobrzański R (2013). Efficient tools for quantum metrology with uncorrelated noise. New J. Phys..

[70] Kaubruegger R (2019). Variational spin-squeezing algorithms on programmable quantum sensors. Phys. Rev. Lett..

[71] Koczor B, Endo S, Jones T, Matsuzaki Y, Benjamin SC (2020). Variational-state quantum metrology. New J. Phys..

[72] Kaubruegger, R., Vasilyev, D. V., Schulte, M., Hammerer, K. & Zoller, P. Quantum variational optimization of Ramsey interferometry and atomic clocks. *Phys. Rev. X* **11**, 041045 (2021).

[73] van Trees, H. L. Detection, Estimation and Modulation Theory, vol. I (Wiley, 1968).

[74] Hovhannisyan KV (2021). Optimal quantum thermometry with coarse-grained measurements. PRX Quantum.

[75] Santagati R (2019). Magnetic field learning using a single electronic spin in diamond with one-photon readout at room temperature. Phys. Rev. X.

[76] Boss JM, Cujia KS, Zopes J, Degen CL (2017). Quantum sensing with arbitrary frequency resolution. Science.

[77] Schmitt S (2021). Optimal frequency measurements with quantum probes. npj Quantum Inf..

[78] Holevo AS (1993). A note on covariant dynamical semigroups. Rep. Math. Phys..

[79] Holevo AS (1996). Covariant quantum Markovian evolutions. J. Math. Phys..

[80] Datta A, Zhang L, Thomas-Peter N, Dorner U, Smith BJ, Walmsley IA (2011). Quantum metrology with imperfect states and detectors. Phys. Rev. A.

[81] Demkowicz-Dobrzański, R., Jarzyna, M. & Kołodyński, J. Quantum limits in optical interferometry. In Wolf, E. (ed.) *Progress in Optics*, vol. 60, 345–435 (Elsevier, 2015).

[82] Berry DW, Wiseman HM (2000). Optimal states and almost optimal adaptive measurements for quantum interferometry. Phys. Rev. Lett..

