# Supplementary material for: Quantum metrology with imperfect measurements
Source: Nat Commun. 2022 Nov 15;13:6971. doi: 10.1038/s41467-022-33563-8 (PMC9666656; doi:10.1038/s41467-022-33563-8)
Supplement: Supplementary file 1 — Supplementary Information [file 41467_2022_33563_MOESM1_ESM.pdf]

Supplementary information: "Quantum metrology with imperfect measurements"

Yink Loong Len,[1,*] Tuvia Gefen,[2,†] Alex Retzker,[3,4] and Jan Kołodyński[1,‡]
[1] *Centre for Quantum Optical Technologies, Centre of New Technologies, University of Warsaw, Banacha 2c, 02-097 Warszawa, Poland*
[2] *Institute for Quantum Information and Matter, Caltech, Pasadena, USA*
[3] *Racah Institute of Physics, The Hebrew University of Jerusalem, Jerusalem 91904, Givat Ram, Israel*
[4] *AWS Center for Quantum Computing, Pasadena, CA 91125, USA*
(Dated: September 20, 2022)

Supplementary Note 1. Proof of Lemma 1

Consider an imperfect measurement $\mathcal{M} \sim \{M_x\}_x$. Then, for a pure encoded state $\psi(\theta) = |\psi(\theta)\rangle\langle\psi(\theta)|$, and a control unitary $\mathcal{V}_{\vec{\phi}} \sim V_{\vec{\phi}}$ allowing a change of measurement basis, the Fisher information (FI) is given by

$$F = \sum_x \frac{[\partial_\theta(\langle\psi(\theta)|V_{\vec{\phi}}^\dagger M_x V_{\vec{\phi}}|\psi(\theta)\rangle)]^2}{\langle\psi(\theta)|V_{\vec{\phi}}^\dagger M_x V_{\vec{\phi}}|\psi(\theta)\rangle}$$
$$= \sum_x \frac{[\langle\partial_\theta\psi(\theta)|V_{\vec{\phi}}^\dagger M_x V_{\vec{\phi}}|\psi(\theta)\rangle + \text{c.c.}]^2}{\langle\psi(\theta)|V_{\vec{\phi}}^\dagger M_x V_{\vec{\phi}}|\psi(\theta)\rangle)}, \quad (1)$$

where c.c. stands for complex conjugation, and $|\partial_\theta\psi(\theta)\rangle$ is the shorthand for $\partial_\theta|\psi(\theta)\rangle$. We decompose $|\partial_\theta\psi(\theta)\rangle$ into the orthogonal and parallel parts to $|\psi(\theta)\rangle$, i.e.:

$$|\partial_\theta\psi(\theta)\rangle = |\partial_\theta\psi_\perp(\theta)\rangle + |\partial_\theta\psi_\parallel(\theta)\rangle, \quad (2)$$
$$\text{with} \quad |\partial_\theta\psi_\perp(\theta)\rangle := (\mathbb{1} - \psi(\theta))|\partial_\theta\psi(\theta)\rangle,$$
$$|\partial_\theta\psi_\parallel(\theta)\rangle := \psi(\theta)|\partial_\theta\psi(\theta)\rangle.$$

It is straightforward to show that $\langle\partial_\theta\psi_\parallel(\theta)|V_{\vec{\phi}}^\dagger M_x V_{\vec{\phi}}|\psi(\theta)\rangle + \text{c.c.} = 0$, and upon defining $|\psi_\perp(\theta)\rangle := |\partial_\theta\psi_\perp(\theta)\rangle/\sqrt{\langle\partial_\theta\psi_\perp(\theta)|\partial_\theta\psi_\perp(\theta)\rangle}$, Eq. (1) is equal to

$$F = 4\,\gamma(\vec{\phi}, \psi(\theta))\,\langle\partial_\theta\psi_\perp(\theta)|\partial_\theta\psi_\perp(\theta)\rangle, \quad (3)$$

with

$$\gamma(\vec{\phi}, \psi(\theta)) := \frac{1}{4}\sum_x \frac{[\langle\psi_\perp(\theta)|V_{\vec{\phi}}^\dagger M_x V_{\vec{\phi}}|\psi(\theta)\rangle + \text{c.c.}]^2}{\langle\psi(\theta)|V_{\vec{\phi}}^\dagger M_x V_{\vec{\phi}}|\psi(\theta)\rangle}. \quad (4)$$

Note that $4\langle\partial_\theta\psi_\perp(\theta)|\partial_\theta\psi_\perp(\theta)\rangle = \mathcal{F}[\psi(\theta)]$ is nothing but the (perfect) quantum Fisher information (QFI) of $\psi(\theta)$.

The imperfect QFI is thus:

$$\mathcal{F}^{(\text{im})} = \left[\max_{\vec{\phi}} \gamma(\vec{\phi}, \psi(\theta))\right] \mathcal{F}[\psi(\theta)]. \quad (5)$$

Let us denote $\gamma_{\mathcal{M}} := \max_{\vec{\phi}} \gamma(\vec{\phi}, \psi(\theta))$. Clearly by an appropriate choice of $\vec{\phi}$ we can map $|\psi(\theta)\rangle, |\psi_\perp(\theta)\rangle$ to any two arbitrary orthogonal states $|\xi\rangle, |\xi_\perp\rangle$. Therefore, the optimization over $\vec{\phi}$ is basically an optimization over any two orthogonal states $|\xi\rangle, |\xi_\perp\rangle$, namely:

$$\gamma_{\mathcal{M}} = \max_{|\xi\rangle,|\xi_\perp\rangle} \sum_x \frac{\text{Re}\{\langle\xi_\perp|M_x|\xi\rangle\}^2}{\langle\xi|M_x|\xi\rangle}. \quad (6)$$

Evidently, $\gamma_{\mathcal{M}}$ is completely independent of the encoding of the parameter, $\psi(\theta)$, and depends only on the imperfect measurement $\mathcal{M} \sim \{M_x\}_x$. Clearly $\gamma_{\mathcal{M}} \geq 0$, because the FI is non-negative, and $\gamma_{\mathcal{M}} \leq 1$, because the imperfect QFI cannot be bigger than the perfect QFI. The latter can also be verified by using the Cauchy-Schwarz inequality:

$$\sum_x \frac{\text{Re}\{\langle\xi_\perp|M_x|\xi\rangle\}^2}{\langle\xi|M_x|\xi\rangle} \leq \sum_x \frac{\langle\xi_\perp|M_x|\xi_\perp\rangle\langle\xi|M_x|\xi\rangle}{\langle\xi|M_x|\xi\rangle}$$
$$= \sum_x \langle\xi_\perp|M_x|\xi_\perp\rangle = 1. \quad (7)$$

Hence, $0 \leq \gamma_{\mathcal{M}} \leq 1$, and it depends solely on the imperfect measurement $\mathcal{M}$; or, in the common cases discussed in the main text, the noisy detection channel $\mathcal{P}$ that determines the effective $\mathcal{M}$, i.e., with all measurement elements then given by $M_x = \sum_i p(x|i)\,\Pi_i$, where Π is some fixed (perfect) projective measurement, so that the imperfect measurement is completely specified by the stochastic map, $\mathcal{P} \sim \{p(x|i)\}$, representing the readout noise. In such a situation, see also the classical interpretation below, we refer to $\gamma_{\mathcal{M}}$ in Eq. (6) as $\gamma_{\mathcal{P}}$. ■

Supplementary Note 2. Properties of $\gamma_{\mathcal{M}}$

In this section we discuss several properties of $\gamma_{\mathcal{M}}$.

a. Classical interpretation of $\gamma_{\mathcal{P}}$. For commuting $\{M_x\}_x$, namely when the imperfect measurement is specified by the noisy detection channel $\mathcal{P} \sim \{p(x|i)\}$ such that $\forall_x : \; M_x = \sum_i p(x|i)\,\Pi_i$, $\gamma_{\mathcal{P}} \equiv \gamma_{\mathcal{M}}$ has a classical interpretation. Note that in this case Eq. (6) reads:

$$\gamma_{\mathcal{P}} = \max_{\mathbf{a},\mathbf{b}} \sum_x \frac{\left(\sum_i p(x|i)\,a_i b_i\right)^2}{\sum_i p(x|i)\,a_i^2}, \quad (8)$$

where $\mathbf{a}, \mathbf{b}$ are real normalised, orthogonal vectors: $\mathbf{a}\cdot\mathbf{b} = 0$, $|\mathbf{a}|^2 = |\mathbf{b}|^2 = 1$. It is simple to see that $\mathbf{a}, \mathbf{b}$ can be

assumed to be real vectors. In order to gain further intuition, we can define $p_i := a_i^2$ and a 'derivative' $\mathrm{d}p_i := a_i b_i$ such that then $a_i = \sqrt{p_i}$ and $b_i = 2\mathrm{d}(\sqrt{p_i})$. As a result, it can be seen that the constraints of $|\mathbf{a}|^2 = 1$, $\mathbf{a}\cdot\mathbf{b} = 0$, $|\mathbf{b}|^2 = 1$ are equivalent to the constraints: $\sum_i p_i = 1$, $\sum_i \mathrm{d}p_i = 0$, $\sum_i \frac{\mathrm{d}p_i^2}{p_i} = 1$. In nuce, $\{p_i\}_i$ is a probability distribution, $\{\mathrm{d}p_i\}_i$ is the derivative vector of the probability distribution and the constraint of $\sum_i \frac{\mathrm{d}p_i^2}{p_i} = 1$ is a normalization constraint: the original FI equals to 1. In this new notation, Eq. (8) reads:

$$\gamma_{\mathcal{P}} = \max_{\boldsymbol{p},\mathbf{d}\boldsymbol{p}} \sum_x \frac{\left(\sum_i p(x|i)\,\mathrm{d}p_i\right)^2}{\sum_i p(x|i)\,p_i}, \tag{9}$$

with the constraint of $\sum_i \frac{\mathrm{d}p_i^2}{p_i} = 1$. Hence, $\gamma_{\mathcal{P}}$ can interpreted as the *optimal noisy classical FI*, optimised over all $\{p_i\}_i, \{\mathrm{d}p_i\}_i$ with the original FI of 1.

b. Data processing inequality $\gamma_{\mathcal{P}_2\circ\mathcal{P}_1} \leq \gamma_{\mathcal{P}_1}$. Let us consider again the setting of $\gamma_{\mathcal{P}}$ in Eq. (8) specified by the noisy detection channel, $\mathcal{P}$, that constitutes a stochastic map with entries $[\mathcal{P}]_{xi} = p(x|i)$ and yields the imperfect measurement $\{M_x = \sum_i [\mathcal{P}]_{xi}\Pi_i\}_x$. Now, considering a composition of any two noisy detection channels $\mathcal{P}_2\circ\mathcal{P}_1$ as an effective stochastic map, the corresponding imperfect measurement simply reads $\{\tilde{M}_x = \sum_i [\mathcal{P}_2\mathcal{P}_1]_{xi}\Pi_i\}_x$. We demonstrate that the resulting γ-coefficient (8) obtained via such a composition must be contractive, i.e. $\gamma_{\mathcal{P}_2\circ\mathcal{P}_1} \leq \gamma_{\mathcal{P}_1}$.

We prove this by first observing that Eq. (9) satisfies $\gamma_{\mathcal{P}} = \max_{\boldsymbol{p},\mathbf{d}\boldsymbol{p}} \sum_x \frac{\left(\sum_i p(x|i)\mathrm{d}p_i\right)^2}{\sum_i p(x|i)p_i} \leq \max_{\boldsymbol{p},\mathbf{d}\boldsymbol{p}} \sum_i \frac{\mathrm{d}p_i^2}{p_i} = 1$, which follows from the Cauchy-Schwarz inequality $\sum_x \frac{\left(\sum_i p(x|i)\mathrm{d}p_i\right)^2}{\sum_i p(x|i)p_i} \leq \sum_x p(x|i)\left(2\mathrm{d}\sqrt{p_i}\right)^2 = \sum_i \frac{\mathrm{d}p_i^2}{p_i}$. Now, if we consider Eq. (9) but for $\mathcal{P} = \mathcal{P}_2\circ\mathcal{P}_1$, we have $\gamma_{\mathcal{P}} = \max_{\boldsymbol{p},\mathbf{d}\boldsymbol{p}} \sum_x \frac{\left(\sum_{j,k} p_2(x|k)p_1(k|j)\mathrm{d}p_j\right)^2}{\sum_{j,k} p_2(x|k)p_1(k|j)p_j}$. Hence, defining $p'_k = \sum_j p_1(k|j)\,p_j$, we similarly obtain by Cauchy-Schwarz inequality: $\gamma_{\mathcal{P}} = \max_{\boldsymbol{p},\mathbf{d}\boldsymbol{p}} \sum_x \frac{\left(\sum_k p_2(x|k)\mathrm{d}p'_k\right)^2}{\sum_k p_2(x|k)p'_k} \leq \max_{\boldsymbol{p},\mathbf{d}\boldsymbol{p}} \sum_k \frac{\left(\mathrm{d}p'_k\right)^2}{p'_k} = \gamma_{\mathcal{P}_1}$, which completes the proof.

c. Monotonically increasing with the number of probes, i.e. $\forall_{\mathcal{M}}:\ \gamma_{\mathcal{M}} \leq \gamma_{\mathcal{M}\otimes I} \leq \gamma_{\mathcal{M}\otimes\mathcal{M}}$. Let us first prove that $\gamma_{\mathcal{M}\otimes I} \leq \gamma_{\mathcal{M}\otimes\mathcal{M}}$ for any imperfect measurement $\mathcal{M}$, which follows from convexity. In general, for any two positive semidefinite operators $M_1, M_2 \geq 0$:

$$\frac{\left(\mathrm{Re}\langle\xi_\perp|M_1|\xi\rangle\right)^2}{\langle\xi|M_1|\xi\rangle} + \frac{\left(\mathrm{Re}\langle\xi_\perp|M_2|\xi\rangle\right)^2}{\langle\xi|M_2|\xi\rangle} \geq \frac{\left(\mathrm{Re}\langle\xi_\perp|M_1+M_2|\xi\rangle\right)^2}{\langle\xi|M_1+M_2|\xi\rangle}, \tag{10}$$

which follows from the convexity of the function $\frac{x^2}{y}$, where we take $x_i = \mathrm{Re}\langle\xi_\perp|M_i|\xi\rangle$, $y_i = \langle\xi|M_i|\xi\rangle$. This convexity implies:

$$\sum_{j,k} \frac{\left(\mathrm{Re}\langle\xi_\perp|M_j\otimes M_k|\xi\rangle\right)^2}{\langle\xi|M_j\otimes M_k|\xi\rangle} \geq \sum_j \frac{\left(\mathrm{Re}\langle\xi_\perp|\sum_k M_j\otimes M_k|\xi\rangle\right)^2}{\langle\xi|\sum_k M_j\otimes M_k|\xi\rangle} = \sum_j \frac{\left(\mathrm{Re}\langle\xi_\perp|M_j\otimes I|\xi\rangle\right)^2}{\langle\xi|M_j\otimes I|\xi\rangle} \tag{11}$$

and, hence, $\gamma_{\mathcal{M}\otimes I} \leq \gamma_{\mathcal{M}\otimes\mathcal{M}}$.

Now, $\gamma_{\mathcal{M}} \leq \gamma_{\mathcal{M}\otimes I}$ is assured by the fact that the maximisation over all orthogonal $|\xi\rangle$ and $|\xi_\perp\rangle$ in Eq. (6) defining 2-dimensional subspace in the support of $\mathcal{M}$, is trivially contained within the maximisation over all orthogonal $|\tilde{\xi}\rangle, |\tilde{\xi}_\perp\rangle$ lying in the support of $\mathcal{M}\otimes I$. In particular, for any two $|\xi\rangle$ and $|\xi_\perp\rangle$ one may choose $|\tilde{\xi}\rangle = |\xi\rangle|\chi\rangle$ and $|\tilde{\xi}_\perp\rangle = |\xi_\perp\rangle|\chi\rangle$ with any $|\chi\rangle$, so that

$$\sum_j \frac{\left(\mathrm{Re}\langle\xi_\perp|M_j|\xi\rangle\right)^2}{\langle\xi|M_j|\xi\rangle} = \sum_j \frac{\left(\mathrm{Re}\langle\chi|\langle\xi_\perp|M_j\otimes I|\xi\rangle|\chi\rangle\right)^2}{\langle\chi|\langle\xi|M_j\otimes I|\xi\rangle|\chi\rangle}. \tag{12}$$

d. Sufficient condition for $\gamma_{\mathcal{M}} = 1$. We mention in the main text that a sufficient condition for a perfect QFI, i.e. $\gamma_{\mathcal{M}} = 1$, is perfect distinguishability between two states: there exist two orthogonal states, $|\xi\rangle$ and $|\xi_\perp\rangle$, such that for every M_x either $M_x|\xi\rangle = 0$ or $M_x|\xi_\perp\rangle = 0$. In order to see this, observe from the Cauchy-Schwarz inequality (7) that $\gamma_{\mathcal{M}} = 1$ if and only if there exist $|\xi\rangle, |\xi_\perp\rangle$ such that $\sqrt{M_x}|\xi\rangle \propto \sqrt{M_x}|\xi_\perp\rangle$ for every x. It is straightforward to see that given perfect distinguishability this condition is satisfied, with the proportionality constant being exactly zero for all x.

Supplementary Note 3. Unitary encoding with two-outcome measurement for a qubit: Optimal state and measurement

Using Bloch representation, our initial probe state is $\rho = \frac{1}{2}\left(\mathbb{1} + \mathbf{r}_0\cdot\boldsymbol{\sigma}\right)$, where the real Bloch vector has the usual constraint $|\mathbf{r}_0|^2 = \mathrm{r}_{0\mathrm{x}}^2 + \mathrm{r}_{0\mathrm{y}}^2 + \mathrm{r}_{0\mathrm{z}}^2 \leq 1$ with equality for pure state. After the encoding with $U_\theta = \mathrm{e}^{\mathrm{i}h\theta}$, $h = \sigma_z/2$, ρ evolves to $\rho(\theta) = \frac{1}{2}\left(\mathbb{1} + \mathbf{r}(\theta)\cdot\boldsymbol{\sigma}\right)$, where $\mathrm{r_x}(\theta) = \mathrm{r}_{0\mathrm{x}}\cos(\theta) + \mathrm{r}_{0\mathrm{y}}\sin(\theta)$, $\mathrm{r_y}(\theta) = -\mathrm{r}_{0\mathrm{x}}\sin(\theta) + \mathrm{r}_{0\mathrm{y}}\cos(\theta)$, and $\mathrm{r_z}(\theta) = \mathrm{r}_{0\mathrm{z}}$. Moreover, $\dot{\mathbf{r}}(\theta) = \partial_\theta\mathbf{r}(\theta)$ is perpendicular to $\mathbf{r}(\theta)$, with $|\dot{\mathbf{r}}|^2 = \mathrm{r}_{0\mathrm{x}}^2 + \mathrm{r}_{0\mathrm{y}}^2 \leq 1$. The two-outcome measurement prior to the stochastic mapping are described by the operators $\Pi_{1,\vec{\phi}} = \frac{1}{2}\left(s\mathbb{1} + \mathbf{m}(\vec{\phi})\cdot\boldsymbol{\sigma}\right)$ and $\Pi_{2,\vec{\phi}} = \frac{1}{2}\left((2-s)\mathbb{1} - \mathbf{m}(\vec{\phi})\cdot\boldsymbol{\sigma}\right)$, with the positive constraints $0 < m \equiv |\mathbf{m}| \leq s^* \equiv \min\{s, 2-s\} \leq 1$. In the main text we consider from onset projective measurements with $m = 1$, but for the sake of mathematical completeness, let us for now allow any two-outcome measurement, and show later $m = 1$ is optimal indeed.

With $\vec{\phi} = \{m, \varphi, \vartheta\}$, we parametrize $\mathbf{m}(\vec{\phi}) = m\big[\cos\varphi(\cos\vartheta\,\mathbf{b}_1 + \sin\vartheta\,\mathbf{b}_2) + \sin\varphi\,\mathbf{b}_3\big]$ in the local Cartesian basis $\{\mathbf{b}_1 = \mathbf{r}(\theta), \mathbf{b}_2 = \dot{\mathbf{r}}/|\dot{\mathbf{r}}|, \mathbf{b}_3 = \mathbf{b}_1 \times \mathbf{b}_2\}$. The respective outcome probabilities are $p_{\theta,\vec{\phi}}(1) = \frac{1}{2}\big(s + \mathbf{m}\cdot\mathbf{r}\big) = \frac{1}{2}(s + m\cos\varphi\cos\vartheta)$ and $p_{\theta,\vec{\phi}}(2) = 1 - p_{\theta,\vec{\phi}}(1)$, while $\dot{p}_{\theta,\vec{\phi}}(1) = -\dot{p}_{\theta,\vec{\phi}}(2) = \frac{1}{2}m\cos\varphi\sin\vartheta|\dot{\mathbf{r}}|$. Note that, while the θ dependence are not seen explicit here, they are present still, as φ and ϑ are defined with respect to θ. For noisy detection channel that is specified by the stochastic mapping $\mathcal{P} \sim \{p(x|i)\}$, i.e. $\mathcal{M} \sim \{M_x = \sum_i p(x|i)\Pi_i\}$, we have $q_{\theta,\vec{\phi}}(x) = \sum_i p(x|i)p_{\theta,\vec{\phi}}(i)$, and the FI, $F = \sum_x f_x$, with

$$f_x = \dot{q}_{\vec{\phi},\theta}(x)^2/q_{\theta,\vec{\phi}}(x) = \frac{\frac{1}{2}a_x^2}{b_x y^2 + c_x y}\sin^2\vartheta|\dot{\mathbf{r}}|^2 \tag{13}$$

where $y \equiv (m\cos\varphi)^{-1} \geq y^* \equiv 1/s^* \geq 1$, $a_x = p(x|1) - p(x|2), b_x = p(x|1) + (2-s)p(x|2)$, and $c_x = a_x\cos\vartheta$.

Consider now maximization of f_x over the input state and the measurement. Evidently, we should choose the input state such that $|\dot{\mathbf{r}}| = 1$, i.e., pure state that lies in the equatorial plane of the Bloch sphere. This choice also means that $\mathbf{b}_3$ is now $\mathbf{e}_\mathrm{z}$. Moreover, we should minimize the function $g_x(y) = b_x y^2 + c_x y$ in the denominator, subject to $y \geq y^*$. First then, we should choose $2 - s = s^*$ in b_x. Next, since g_x is a convex function, and the roots of g_x are 0 and $-c_x/b_x$ for which $|c_x/b_x| \leq y^*$, we have $\min_{y\geq y^*} g_x(y) = g_x(y^*)$. That is, we have $\varphi = 0$ and $m = s^*$, such that $\mathbf{m}$ has no $\mathbf{b}_3 = \mathbf{e}_\mathrm{z}$ component.

To confirm that we should always choose projective measurement before the noisy detection channel whenever possible, i.e., $m = s^* = 1$, we put in $s^* = 1/y^*$ into $g_x(y^*)$ for an explicit convex function of y^*. One can then verify readily that the roots are now not greater than 1, and therefore, the optimal choice of y^* is 1. Finally, as all the above optimizations hold for all f_x, it follows that they apply to the total FI, and therefore

$$\begin{aligned}\bar{\mathcal{F}}^{(\mathrm{im})} &= \max_\rho \max_{\vec{\phi}} F \\ &= \max_\vartheta \sum_x \frac{\frac{1}{2}\big(p(x|1) - p(x|2)\big)^2\sin^2\vartheta}{p(x|1) + p(x|2) + \big(p(x|1) - p(x|2)\big)\cos\vartheta}.\end{aligned} \tag{14}$$

Note that as Eq. (14) depends on ϑ, which is an angle defined relative to $\mathbf{r}(\theta)$, it follows that in this case here we have a freedom to fix either the measurement or the input state and optimize over the other, as long as both are restricted to the equatorial plane. In particular, we may fix $\rho = |+_\mathrm{y}\rangle\langle+_\mathrm{y}|$, i.e., $\mathbf{r}_0 = \mathbf{e}_\mathrm{y}$, and optimize over $\{\Pi_{i,\vec{\phi}} = |\Pi_i\rangle\langle\Pi_i|\}$ with $|\Pi_{1,2}\rangle = (|0\rangle \pm e^{\mathrm{i}\phi}|1\rangle)/\sqrt{2}$, i.e., $\mathbf{m} = \cos\phi\,\mathbf{e}_\mathrm{x} + \sin\phi\,\mathbf{e}_\mathrm{y}$. Equivalently, we may optimize over $\rho = |\psi\rangle\langle\psi|$ with $|\psi\rangle = e^{\mathrm{i}\phi\sigma_\mathrm{z}/2}|+_\mathrm{y}\rangle = (|0\rangle + \mathrm{i}e^{\mathrm{i}\phi}|1\rangle)/\sqrt{2}$, i.e., $\mathbf{r}_0 = -\sin\phi\,\mathbf{e}_\mathrm{x} + \cos\phi\,\mathbf{e}_\mathrm{y}$, with fixed $\Pi_{1,\vec{\phi}} = |+\rangle\langle+|, \Pi_{2,\vec{\phi}} = |-\rangle\langle-|$, i.e., $\mathbf{m} = \mathbf{e}_\mathrm{x}$. Both give the same expression with $\vartheta = \theta + \phi - \pi/2$ in Eq. (14), which turns into Eq. (9) in the main text.

Supplementary Note 4. Relations of quantum metrology with imperfect measurements to the "standard" setting of noisy parameter encoding

In this section, we discuss in detail the differences and relation between our quantum metrology with imperfect measurement model and the "standard" noisy metrology model. In the latter, the measurement is taken to be perfect, and the noise is described by a CPTP channel that acts before the measurement stage.

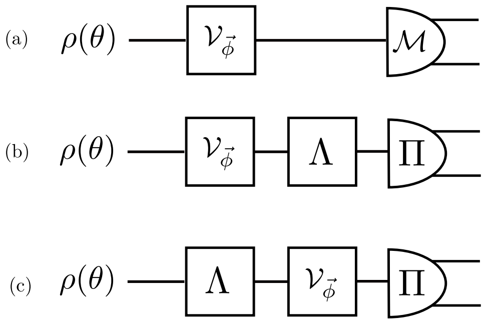

Supplementary Figure 1. **Different models for noisy quantum metrology.** (a) Quantum metrology with imperfect measurement $\mathcal{M}$. $\mathcal{V}_{\vec{\phi}}$ is some control unitary operation for optimizing the measurement basis. (b) Equivalent picture of (a), with Π being a projective measurement which can be chosen and fixed arbitrarily, and Λ is a CPTP channel. (c) Quantum metrology with "standard" noisy parameter encoding, where the noise, described by a CPTP channel Λ, is *independent* of the choice of the measurement settings $\vec{\phi}$.

Firstly, let us denote $\mathcal{B}(\mathcal{H}_d)$ as the set of bounded linear operators on the Hilbert space $\mathcal{H}_d$ with dimension d. Then, recall that our quantum metrology with imperfect measurement on the physical level is as depicted in Suppl. Fig. 1(a): an encoded qudit described by the state $\rho(\theta) \in \mathcal{B}(\mathcal{H}_d)$, undergoes a control unitary operation $\mathcal{V}_{\vec{\phi}} \sim \{V_{\vec{\phi}}\}$ such that $\rho(\theta) \to \mathcal{V}_{\vec{\phi}}[\rho(\theta)] = V_{\vec{\phi}}\rho(\theta)V_{\vec{\phi}}^\dagger$, which is then subject to an imperfect measurement $\mathcal{M} \sim \{M_x\}_{x=1}^{|X|}$. Here, all the M_x and the $V_{\vec{\phi}}$ are elements of $\mathcal{B}(\mathcal{H}_d)$ as well. The probability of getting the outcome x is given by the Born's rule, $q_{\theta,\vec{\phi}}(x) = \mathrm{Tr}\{M_x V_{\vec{\phi}}\rho(\theta)V_{\vec{\phi}}^\dagger\}$.

We can also think of it in a mathematically equivalent picture, as in Suppl. Fig. 1(b), where we describe the imperfect measurement $\mathcal{M} \sim \{M_x\}_{x=1}^{|X|}$ rather by a noisy CPTP channel Λ that is followed by a perfect projective measurement $\Pi \sim \{\Pi_x = |x\rangle\langle x|\}_{x=1}^{|X|}$. That is, we can always find some quantum (CPTP) map $\Lambda \sim \{k_\ell\}_\ell$ that allows for the *conjugate-map decomposition* of $\mathcal{M}$, i.e.:

$$\forall_{x=1,\cdots,|X|}: \quad M_x = \Lambda^\dagger[\Pi_x] = \sum_\ell k_\ell^\dagger \Pi_x k_\ell, \tag{15}$$

or, rewriting the above in a compact way:

$$\mathcal{M} = \Lambda^\dagger[\Pi]. \tag{16}$$

Note that: (i) the channel $\Lambda : \mathcal{B}(\mathcal{H}_d) \to \mathcal{B}(\mathcal{H}_{|X|})$ acts on operators of d dimension and outputs operators of $|X|$ dimension; (ii) given $\mathcal{M}$, there are multiple Λ (and Π) satisfying Eq. (16); and (iii) Λ is defined *independently* of the control unitary $\mathcal{V}_{\vec{\phi}}$—one can consider the l.h.s. of Eq. (15) as $V_{\vec{\phi}}^\dagger M_x V_{\vec{\phi}}$ instead, and define $\vec{\phi}$-dependent Λ, but doing so is neither necessary nor helpful.

One can prove that the condition (15), or (16), can always be satisfied, by considering as an example the *quantum-classical channel* that we used as well in the main text (Lemma 3 and Corollary 2) [1], namely,

$$\Lambda \sim \left\{ |x\rangle \langle i| \sqrt{M_x} \right\}_{x,i} \qquad x = 1, \dots, |X|; \quad i = 1, \cdots, d. \tag{17}$$

Another example of a CPTP map that also always satisfies Eq. (16) is

$$\Lambda \sim \left\{ \sum_{x=1}^{|X|} |x\rangle \langle i| \sqrt{M_x} \right\}_{i=1}^{d}, \tag{18}$$

whose rank is now d, rather than $d \cdot |X|$ in Eq. (17).

Note that, both the orthogonal sets of basis ket $\{|x\rangle\}_x$ and bra $\{\langle i|\}_i$ in Eqs. (17) and (18) can essentially be understood as flags, and can be chosen arbitrarily. Importantly, despite this equivalent picture in Fig.1(b), it is however still not the same as the "standard" noisy metrology scheme as depicted in Fig.1(c), where the noise, described by a CPTP map Λ, acts *independently* of the choice of the control unitary $\mathcal{V}_{\vec{\phi}}$. The crucial difference is that, in the former the control operation $\mathcal{V}_{\vec{\phi}}$ is applied *before* the channel Λ, whereas in the latter it is applied *after* the channel Λ. Then, unless $\mathcal{V}_{\vec{\phi}}$ commutes with Λ, which is hardly ever true, the two noise model will not be equivalent.

Let us still study more closely the two models from the perspective of parameter estimation, whereby the focus is on the QFI and channel QFI (perfect versus imperfect). Denote $F[\rho(\theta), \mathcal{M}]$ as the classical Fisher information for θ with the state $\rho(\theta)$ and measurement $\mathcal{M} \sim \{M_x\}_x$, i.e., $F[\rho(\theta), \mathcal{M}] = \sum_x \frac{(\partial_\theta \mathrm{Tr}\{\rho(\theta) M_x\})^2}{\mathrm{Tr}\{\rho(\theta) M_x\}}$. Then, for the imperfect measurement model, the imperfect QFI is given by (see Eq. (5) in the main text):

$$\begin{aligned}\mathcal{F}^{(\mathrm{im})} &= \max_{\vec{\phi}} F[\mathcal{V}_{\vec{\phi}}[\rho(\theta)], \Lambda^\dagger[\Pi]] \\ &:= F[\mathcal{V}_{\mathrm{opt}}[\rho(\theta)], \Lambda^\dagger[\Pi]] = F[(\Lambda \circ \mathcal{V}_{\mathrm{opt}})[\rho(\theta)], \Pi].\end{aligned} \tag{19}$$

Note that the optimal control unitary $\mathcal{V}_{\mathrm{opt}} \sim \{V_{\mathrm{opt}}\}$ generally depends on the given $\rho(\theta)$, though for simplicity of the notation we will not write out this dependence explicitly for now. It then follows that

$$\begin{aligned}\mathcal{F}^{(\mathrm{im})} &\le \max_W F[(\Lambda \circ \mathcal{V}_{\mathrm{opt}})[\rho(\theta)], W \Pi W^\dagger] \\ &:= \mathcal{F}[(\Lambda \circ \mathcal{V}_{\mathrm{opt}})[\rho(\theta)]],\end{aligned} \tag{20}$$

where W is a unitary operator in $\mathcal{B}(\mathcal{H}_{|X|})$, and so we may formally upper bound the imperfect QFI by the QFI of $(\Lambda \circ \mathcal{V}_{\mathrm{opt}})[\rho(\theta)]$, which can be interpreted as a state that undergoes a "standard" noisy channel $\Lambda \circ \mathcal{V}_{\mathrm{opt}}$. Moreover, given the imperfect measurement $\mathcal{M}$, one can further optimize over different possible Λ that satisfies Eq. (16), and get

$$\mathcal{F}^{(\mathrm{im})} \le \min_{\substack{\Lambda \\ \Lambda^\dagger[\Pi] = \mathcal{M}}} \mathcal{F}[(\Lambda \circ \mathcal{V}_{\mathrm{opt}})[\rho(\theta)]]. \tag{21}$$

Despite the established formal relations Eqs. (20) and (21), note that $\Lambda \circ \mathcal{V}_{\mathrm{opt}}$ is defined using the knowledge about optimal control unitary. However, if we had known $\mathcal{V}_{\mathrm{opt}}$, we would have already in fact obtained $\mathcal{F}^{(\mathrm{im})}$, and there is no need for the upper bounds. In other words, the formal bounds Eqs. (20) and (21) are not that meaningful in practice.

A. Proof of the Observation 1 in Methods

Still, there is a special case worth mentioning, where we can indeed meaningfully evaluate a QFI-based upper bound without solving exactly for the $\mathcal{V}_{\mathrm{opt}}$. Suppose that from the symmetry of the estimation problem we know that the optimal control operation must also carry it, so that its optimisation may be restricted to elements in some compact group G, i.e., $\mathcal{V}_{\mathrm{opt}} \in G$. Then, if Λ satisfying Eq. (16) is known to be G-*covariant*, i.e.,

$$\forall_{g \in G}: \ \Lambda \circ \mathcal{V}_g = \mathcal{W}_g \circ \Lambda, \tag{22}$$

where $\mathcal{W}_g$ is some unitary representation of G in $\mathcal{H}_{|X|}$— in particular, for $\mathcal{W}_{\mathrm{opt}}$ correspondingly to $\mathcal{V}_{\mathrm{opt}}$ in Eq. (22), we have from Eq. (20) that

$$\mathcal{F}^{(\mathrm{im})} \le \mathcal{F}[(\mathcal{W}_{\mathrm{opt}} \circ \Lambda)[\rho(\theta)]] = \mathcal{F}[\Lambda[\rho(\theta)]], \tag{23}$$

where the equality in (23) follows from the fact that QFI is invariant under parameter-independent unitary transformation. ■

Extension to channel QFI is straightforward. Let us denote the (perfect) parameter-encoding channel as $\mathcal{E}_\theta$, such that $\rho(\theta) = \mathcal{E}_\theta[\rho]$ for the input probe state ρ, which we will eventually optimize over. For the imperfect measurement case, we have by definition (see Eq. (5) in the main text):

$$\begin{aligned}\bar{\mathcal{F}}^{(\mathrm{im})} &= \max_\rho \max_{\vec{\phi}} F[(\mathcal{V}_{\vec{\phi}} \circ \mathcal{E}_\theta)[\rho], \Lambda^\dagger[\Pi]] \\ &:= F[(\Lambda \circ \mathcal{V}_{\mathrm{opt}} \circ \mathcal{E}_\theta)[\rho_{\mathrm{opt}}], \Pi] \\ &= \sum_x \frac{(\mathrm{Tr}\{(\Lambda \circ \mathcal{V}_{\mathrm{opt}} \circ \dot{\mathcal{E}}_\theta)[\rho_{\mathrm{opt}}] \Pi_x\})^2}{\mathrm{Tr}\{(\Lambda \circ \mathcal{V}_{\mathrm{opt}} \circ \mathcal{E}_\theta)[\rho_{\mathrm{opt}}] \Pi_x\}},\end{aligned} \tag{24}$$

where $\mathcal{V}_{\rm opt}$ and $\rho_{\rm opt}$ are respectively the optimal control unitary and input state, and $\dot{\mathcal{E}}_\theta = \partial_\theta \mathcal{E}_\theta$ is the derivative of the encoding channel w.r.t. the parameter, such that for any $\rho(\theta) = \mathcal{E}_\theta[\rho]$, $\partial_\theta\rho(\theta) = \dot{\mathcal{E}}_\theta[\rho]$. Similarly to Eq. (20), one then obtains

$$\begin{aligned}\bar{\mathcal{F}}^{(\rm im)} &\leq \max_\sigma \max_W F[(\Lambda\circ\mathcal{V}_{\rm opt}\circ\mathcal{E}_\theta)[\sigma], W\Pi W^\dagger] \\ &:= \bar{\mathcal{F}}[(\Lambda\circ\mathcal{V}_{\rm opt}\circ\mathcal{E}_\theta)], \end{aligned} \tag{25}$$

where σ is some state in $\mathcal{H}_d$, W is a unitary operator in $\mathcal{B}(\mathcal{H}_{|X|})$, and $\bar{\mathcal{F}}$ is the channel QFI. Again, although Eq. (25) now provides a formal relation between the imperfect channel QFI and the channel QFI of a "standard" noisy encoding $(\Lambda\circ\mathcal{V}_{\rm opt}\circ\mathcal{E}_\theta)$, it requires knowledge of the optimal control unitary $\mathcal{V}_{\rm opt}$ (which implicitly depends on the optimal state $\rho_{\rm opt}$), and is hence in general not immediately applicable.

Still, when we know that, thanks to some symmetry of the problem, the optimisation over control $\mathcal{V}_{\vec{\phi}}$ can be restricted to elements of some compact group G, meaningful upper bound on the imperfect channel QFI can be formulated. Firstly, following directly from the Observation 1, in case the conjugate map Λ satisfies not only Eq. (16) but also the G-covariant condition Eq. (22), we can immediately conclude by maximising Eq. (23) over the input probe-states that $\bar{\mathcal{F}}^{(\rm im)} \leq \bar{\mathcal{F}}[(\Lambda\circ\mathcal{E}_\theta)]$.

B. *Proof of the Observation 2 in Methods*

Secondly, suppose that the encoding channel $\mathcal{E}_\theta$, as well as its derivate, $\dot{\mathcal{E}}_\theta = \partial_\theta\mathcal{E}_\theta$, are both G-covariant locally around the parameter value θ, i.e.,

$$\begin{aligned}\forall_{g\in G}:\ \mathcal{V}_g\circ\mathcal{E}_\theta &= \mathcal{E}_\theta\circ\mathcal{W}_g, \\ \mathcal{V}_g\circ\dot{\mathcal{E}}_\theta &= \dot{\mathcal{E}}_\theta\circ\mathcal{W}_g,\end{aligned} \tag{26}$$

where $\mathcal{W}_g$ is some unitary representation of G in $\mathcal{H}_d$— in particular, $\mathcal{W}_{\rm opt}$ correspondingly for $\mathcal{V}_{\rm opt}$. In this case, from (25),

$$\bar{\mathcal{F}}^{(\rm im)} \leq \bar{\mathcal{F}}[(\Lambda\circ\mathcal{E}_\theta\circ\mathcal{W}_{\rm opt})] = \bar{\mathcal{F}}[(\Lambda\circ\mathcal{E}_\theta)], \tag{27}$$

where the equality in (27) follows from the fact that channel QFI is invariant under parameter-independent unitary transformation on the input probe state. Note that it is necessary to include the local G-covariant condition for $\dot{\mathcal{E}}_\theta$ here, as FI is not a function of the state alone but also its derivative, c.f. explicitly Eq. (24) and Eq. (28) below. ■

C. *Computing $\bar{\mathcal{F}}[(\Lambda\circ\mathcal{E}_\theta)]$ in Eq. (27) as an SDP via a 'seesaw' method*

The QFI is defined as a function of a quantum state $\rho\equiv\rho(\theta)$ and its derivative $\dot{\rho}\equiv\partial_\theta\rho(\theta)$, as follows [2]:

$$\mathcal{F}[\rho,\dot{\rho}] := \begin{array}{c}\mathrm{Tr}\{\rho L^2\} = \mathrm{Tr}\{\dot{\rho}L\} \\ \text{s.t. } \dot{\rho} = \frac{1}{2}\{\rho, L\}\end{array}. \tag{28}$$

Equivalently, it may be expressed as the maximisation of the *error propagation formula* over all the quantum observables, i.e., Hermitian operators $O = O^\dagger$, as [3]:

$$\mathcal{F}[\rho,\dot{\rho}] = \max_{O=O^\dagger}\frac{\left|\left\langle\dot{O}\right\rangle\right|^2}{\Delta^2 O} = \max_{O=O^\dagger}\frac{|\mathrm{Tr}\{\dot{\rho}O\}|^2}{\mathrm{Tr}\{\rho O^2\} - \mathrm{Tr}\{\rho O\}^2}, \tag{29}$$

which is always maximised by $O_{\rm opt} = L - \mathrm{Tr}\{\rho L\}$ with L being the SLD operator defined implicitly in Eq. (28).

However, the fraction in Eq. (29) can always be rewritten by introducing another maximisation, i.e.:

$$\frac{\left|\left\langle\dot{O}\right\rangle\right|^2}{\Delta^2 O} = \max_{\alpha\in\mathbb{R}}\left\{-\alpha^2\Delta^2 O + 2\alpha\left|\left\langle\dot{O}\right\rangle\right|\right\}, \tag{30}$$

with the maximum occurring at $\alpha_{\rm opt} = \frac{|\langle\dot{O}\rangle|}{\Delta^2 O}$. Hence, we may write again Eq. (29) as

$$\begin{aligned}\mathcal{F}[\rho,\dot{\rho}] &= \max_{O=O^\dagger}\max_{\alpha\in\mathbb{R}}\left\{-\alpha^2\Delta^2 O + 2\alpha\left|\left\langle\dot{O}\right\rangle\right|\right\} \qquad (31)\\ &= \max_{O'=O'^\dagger}\left\{-\Delta^2 O' + 2\left|\left\langle\dot{O}'\right\rangle\right|\right\}, \qquad (32)\end{aligned}$$

while noticing that the two maximisations can be recast into one by defining $O' := \alpha O$. Moreover, for any O' above we may define a shifted operator $X := O' - \langle O'\rangle$, so that substituting $O' = X + \mathrm{Tr}\{\rho O'\}$ into Eq. (32), we obtain [4]:

$$\begin{aligned}\mathcal{F}[\rho,\dot{\rho}] &= \max_{X=X^\dagger}\left\{-\left\langle X^2\right\rangle + 2\left|\left\langle\dot{X}\right\rangle\right|\right\} \qquad (33)\\ &= \max_{X=X^\dagger}\left\{-\left\langle X^2\right\rangle + 2\left\langle\dot{X}\right\rangle\right\}, \qquad (34)\end{aligned}$$

where the maximum is now performed over all Hermitian operators $X = X^\dagger$. We have also dropped the absolute value, as $\left\langle\dot{X}\right\rangle = \mathrm{Tr}\{\dot{\rho}X\} = \partial_\theta\mathrm{Tr}\{\rho X\}$ is real, while the first (quadratic) term above is unaffected by the $X\to -X$ transformation—and so must be the maximal value attained in Eq. (34).

Let us note that Eq. (34) constitutes a valid lower bound on the QFI for any fixed X, i.e.

$$\forall_{X=X^\dagger,\rho,\dot{\rho}}:\ -\mathrm{Tr}\{\rho X^2\} + 2\mathrm{Tr}\{\dot{\rho}X\} \ \leq\ \mathcal{F}[\rho,\dot{\rho}], \tag{35}$$

while the optimal $X_{\rm opt}$, yielding the maximum in Eq. (34), is related to the actual optimal observable $O_{\rm opt}$

in Eq. (29) via

$$X_{\rm opt} = O'_{\rm opt} - \langle O'_{\rm opt}\rangle = \alpha_{\rm opt}\left(O_{\rm opt} - \langle O_{\rm opt}\rangle\right) \quad (36)$$

$$= \frac{\left|\left\langle \dot{O}_{\rm opt}\right\rangle\right|}{\Delta^2 O_{\rm opt}}\left(O_{\rm opt} - \langle O_{\rm opt}\rangle\right). \quad (37)$$

Hence, by substituting further $O_{\rm opt} = L - {\rm Tr}\{\rho L\}$, we can explicitly relate $X_{\rm opt}$ to the SLD and the QFI, i.e.:

$$X_{\rm opt} = \frac{\mathcal{F}[\rho,\dot\rho]}{|{\rm Tr}\{\dot\rho L\}|}\left(L - {\rm Tr}\{\rho L\}\right). \quad (38)$$

Let us consider for our purposes the case of unitary parameter encoding, i.e. $\mathcal{E}_\theta = \mathcal{U}_\theta \sim \{{\rm e}^{-{\rm i}\theta H}\}$ so that $\dot\rho = {\rm i}[\rho, H]$, but the following analysis can be directly generalised to allow for arbitrary $\mathcal{E}_\theta$ and $\dot{\mathcal{E}}_\theta$. Then, using the expression (28) for the QFI, we may rewrite the potentially valid—e.g. given the G-covariance (22) or (26)—upper bound on the imperfect QFI $\bar{\mathcal{F}}^{(\rm im)}$ in Eq. (27) for the encoding $U_\theta = {\rm e}^{-{\rm i}\theta H}$ as:

$$\bar{\mathcal{F}}[H,\Lambda] := \bar{\mathcal{F}}[(\Lambda\circ\mathcal{U}_\theta)] \quad (39)$$

$$= \max_{\sigma\geq 0}\ \mathcal{F}[\Lambda[\sigma], \Lambda[{\rm i}[\sigma,H]]] \quad (40)$$

$$= \max_{\sigma\geq 0}\ \begin{array}{c} {\rm i}\,{\rm Tr}\{[\sigma,H]\,\Lambda^\dagger[L]\} \\ {\rm s.t.}\ \Lambda[{\rm i}[\sigma,H]] = \frac{1}{2}\{\Lambda[\sigma], L\}\end{array}. \quad (41)$$

Moreover, we may now use Eq. (34) to further re-express the above channel QFI (for $\Lambda\circ\mathcal{U}_\theta$) as

$$\bar{\mathcal{F}}[H,\Lambda] = \max_{\sigma\geq 0}\max_{X=X^\dagger}\ {\rm Tr}\{\sigma\left(-\Lambda^\dagger[X^2] + 2{\rm i}\left[H, \Lambda^\dagger[X]\right]\right)\}. \quad (42)$$

As a result, we can formulate a numerical *'seesaw' algorithm* that allows us to compute the channel QFI (41) by exploiting Eq. (42), as follows [4]:

1. Select (randomly) a starting state σ_0 and calculate the corresponding QFI $\mathcal{F}$ as well as the SLD L it would lead to in Eq. (41), i.e. without performing the maximisation over the input states σ.

2. Use the obtained $\mathcal{F}$ and L to compute the optimal operator $X_0 = X_{\rm opt}$ according to Eq. (38), which then maximises Eq. (42) for the fixed state σ_0.

3. Maximise the expression (42) over the input states with the operator X_0 being now fixed, in order to determine the best state σ_1 that then yields the tightest lower bound (35) on QFI for X_0.

4. Return to step 1 and use σ_1 as the new starting state.

The above procedure is computationally efficient, as $\mathcal{F}$ and L in step 1 are obtained solving a linear programme, as in Eq. (28), while finding the optimal input state in step 3 corresponds to solving the maximal eigenvalue of a Hermitian operator defined within $(\dots)$ of Eq. (42). Although the convergence of the algorithm is generally assured [4], even if its rate is slow, at any stage it yields a valid lower bound (35) on the channel QFI (42).

D. Maximising $\bar{\mathcal{F}}[(\Lambda\circ\mathcal{E}_\theta)]$ in Eq. (27) further, over all conjugate-map decompositions of an imperfect measurement

Recall that Λ in Eq. (27), and so in Eq. (42), corresponds to some valid conjugate-map decomposition of a given imperfect measurement $\mathcal{M}$, for which it must fulfil the condition (16). As we now show, the above 'seesaw' formulation allows naturally to incorporate in Eq. (42) also the maximisation over all such conjugate maps, i.e. all quantum (CPTP) channels Λ satisfying $\mathcal{M} = \Lambda^\dagger[\Pi]$ for some projective measurement Π.

Let us define the corresponding maximum as

$$\bar{\mathcal{F}}[H,\mathcal{M}] := \max_{\Lambda\in{\rm CPTP}}\ \begin{array}{c}\bar{\mathcal{F}}[H,\Lambda] \\ {\rm s.t.}\ \Lambda^\dagger[\Pi] = \mathcal{M}\end{array}, \quad (43)$$

$$= \max_{\substack{\Lambda\in{\rm CPTP}\\ \sigma\geq 0}}\ {\rm s.t.}\ \begin{array}{c}{\rm i}\,{\rm Tr}\{[\sigma,H]\,\Lambda^\dagger[L]\} \\ \Lambda^\dagger[\Pi] = \mathcal{M} \\ \Lambda\left[{\rm i}[\sigma,H]\right] = \frac{1}{2}\left\{\Lambda\left[\sigma\right], L\right\}\end{array} \quad (44)$$

where have substituted for $\bar{\mathcal{F}}[H,\Lambda]$ according to Eq. (41). Note that in the above expression any projective measurement, Π, can be used as a reference, because $\bar{\mathcal{F}}[H,\mathcal{M}]$ is invariant under the transformation $\Pi \to W\Pi W^\dagger$, $L\to WLW^\dagger$ and $\Lambda[\bullet]\to W\Lambda[\bullet]W^\dagger$, which implies $\Lambda^\dagger[\bullet]\to\Lambda^\dagger[W^\dagger\bullet W]$, for any unitary W.

Now, similarly to Eq. (42), we rewrite Eq. (44) as

$$\bar{\mathcal{F}}[H,\mathcal{M}] = \max_{\substack{\Lambda\in{\rm CPTP}\\ \sigma\geq 0, X=X^\dagger}}\ \begin{array}{c}{\rm Tr}\{\sigma\left(-\Lambda^\dagger[X^2] + 2{\rm i}[H,\Lambda^\dagger[X]]\right)\} \\ {\rm s.t.}\ \Lambda^\dagger[\Pi] = \mathcal{M}\end{array}, \quad (45)$$

which we simplify further by denoting the action of any CPTP map Λ via its Choi-Jamiołkowski (CJ) state [5].

In particular, for any input state σ it is true that $\Lambda[\sigma] = {\rm Tr}_{\rm B}\{\varrho_\Lambda(\mathbb{I}\otimes\sigma^T)\}$, where the CJ state of the map Λ is defined as $\varrho_\Lambda := \Lambda\otimes\mathcal{I}\,[|\mathbb{I}\rangle\rangle\langle\langle\mathbb{I}|]$. Here, any square matrix A defines a bipartite state $|A\rangle\rangle = \sum_{ij} A_{ij}\,|i,j\rangle_{\rm AB} = \sum_{ij} A_{ij}\,|i\rangle_{\rm A}\,|j\rangle_{\rm B}$, so that $|\mathbb{I}\rangle\rangle = \sum_i |i,i\rangle_{\rm AB}$ is the (unnormalised) maximally entangled state. Then, it is straightforward to prove that the CJ state for the conjugate map of Λ, i.e. $\Lambda^\dagger$, is nothing but $\varrho_{\Lambda^\dagger} = \mathbb{S}_{\rm AB}\varrho^*_\Lambda\mathbb{S}_{\rm AB}$, where $\mathbb{S}_{\rm AB}$ is the swap operator such that $\mathbb{S}_{\rm AB}\,|\psi\rangle_{\rm A}\,|\phi\rangle_{\rm B} = |\phi\rangle_{\rm A}\,|\psi\rangle_{\rm B}$ for any states $|\phi\rangle$ and $|\psi\rangle$. Consequently, the action of $\Lambda^\dagger$ corresponds to

$$\forall_{X=X^\dagger}:\ \Lambda^\dagger[X] = {\rm Tr}_{\rm B}\{\varrho_{\Lambda^\dagger}(\mathbb{I}\otimes X^T)\} \quad (46)$$

$$= {\rm Tr}_{\rm B}\{\mathbb{S}_{\rm AB}\varrho^*_\Lambda\mathbb{S}_{\rm AB}(\mathbb{I}\otimes X^T)\mathbb{S}_{\rm AB}\mathbb{S}_{\rm AB}\}$$

$$= {\rm Tr}_{\rm A}\{\varrho^*_\Lambda(X^T\otimes\mathbb{I})\}, \quad (47)$$

and the TP-property of Λ, imposing ${\rm Tr}_{\rm A}\varrho_\Lambda = \mathbb{I}$, ensures consistently that ${\rm Tr}_{\rm B}\varrho_{\Lambda^\dagger} = {\rm Tr}_{\rm B}\{\mathbb{S}_{\rm AB}\varrho^*_\Lambda\mathbb{S}_{\rm AB}\} = ({\rm Tr}_{\rm A}\{\varrho_\Lambda\})^\star = \mathbb{I}$, so that the conjugate map is indeed always unital, i.e. $\Lambda^\dagger[\mathbb{I}] = \mathbb{I}$.

Finally, using Eq. (47) to replace the maximisation over quantum maps Λ in Eq. (45) by the corresponding CJ states ϱ_Λ, we obtain

$$\bar{\mathcal{F}}[H,\mathcal{M}] = \max_{\varrho_\Lambda\geq 0}\max_{\sigma\geq 0}\max_{X=X^\dagger} \begin{array}{l} -\mathrm{Tr}\{\sigma\,\mathrm{Tr}_{\mathrm{A}}\{\varrho_\Lambda^*\left((X^2)^T\otimes\mathbb{I}\right)\}\} + 2\mathrm{i}\mathrm{Tr}\{\sigma\left[H,\mathrm{Tr}_{\mathrm{A}}\{\varrho_\Lambda^*(X^T\otimes\mathbb{I})\}\right]\} \\ \text{s.t. } \forall_x:\ \mathrm{Tr}_{\mathrm{A}}\{\varrho_\Lambda^*\left((\Pi_x)^T\otimes\mathbb{I}\right)\} = M_x,\ \mathrm{Tr}_{\mathrm{A}}\varrho_\Lambda = \mathbb{I} \end{array}$$
$$= \max_{\varrho_\Lambda\geq 0}\max_{\sigma\geq 0}\max_{X=X^\dagger} \begin{array}{l} -\mathrm{Tr}\{\varrho_\Lambda^*\left((X^2)^T\otimes\sigma\right)\} + 2\mathrm{i}\mathrm{Tr}\{\varrho_\Lambda^*(X^T\otimes[\sigma,H])\} \\ \text{s.t. } \forall_x:\ \mathrm{Tr}_{\mathrm{A}}\{\varrho_\Lambda^*\left((\Pi_x)^T\otimes\mathbb{I}\right)\} = M_x,\ \mathrm{Tr}_{\mathrm{A}}\varrho_\Lambda = \mathbb{I} \end{array}, \quad (48)$$

which we evaluate by adding to the aforementioned 'see-saw' algorithm one more step in which we maximise over $\varrho_\Lambda \geq 0$ (given the linear constraints to reproduce the elements of the imperfect measurement), while fixing the input state, σ, and the Hermitian operator, X.

E. *Example 1: Single-qubit phase sensing with bit-flip noise*

Let us first illustrate the subtleties of G-covariance, and limited applicability of the Observation 1 discussed in Supplementary Note 4 A for the *imperfect QFI*, $\mathcal{F}^{(\mathrm{im})}$, by considering the unitary encoding $\mathcal{E}_\theta \sim \{\mathrm{e}^{\mathrm{i}\theta\sigma_z/2}\}$ on a qubit, with imperfect measurement $\mathcal{M} \sim \{M_1 = \mathsf{p}|+\rangle\langle+| + (1-\mathsf{q})|-\rangle\langle-|, M_2 = \mathsf{q}|+\rangle\langle+| + (1-\mathsf{p})|-\rangle\langle-|\}$ with $0 \leq \mathsf{p},\mathsf{q} \leq 1$. Consider also the input probe state in the equatorial Bloch plane, such that for example the encoded state is $\rho(\theta) = |\psi(\theta)\rangle\langle\psi(\theta)|, |\psi(\theta)\rangle = \frac{1}{\sqrt{2}}(|0\rangle + \mathrm{e}^{\mathrm{i}\theta}|1\rangle)$.

As we have established above in Supplementary Note 3, the optimal control unitary in this case must have the structure $V_{\mathrm{opt}} = \mathrm{e}^{\mathrm{i}\varphi_{\mathrm{opt}}\sigma_z}$ for some φ_{opt} and, hence, belongs to the U(1) group with unitary representation: $\mathcal{V}_g \sim \{\mathrm{e}^{\mathrm{i}g\sigma_z}\}$ and $g \in [0,2\pi)$. Consequently, when Λ can be chosen to not only fulfil (16) but also be *phase-covariant* [6–8], i.e. $\forall_g : [\Lambda,\mathcal{V}_g] = 0$, the G-covariance condition (22) is satisfied. For the special case of symmetric mixing with $\mathsf{p} = \mathsf{q}$ and any $0 \leq \mathsf{p} \leq 1$, one can show that the dephasing channel fulfilling (16), $\Lambda_{\mathrm{dep}} \sim \{\sqrt{\mathsf{p}}\mathbb{1}, \sqrt{1-\mathsf{p}}\sigma_z\}$, is phase-covariant and, hence, the upper bound (23) is applicable. Moreover, in this case the bound (23) is tight: by taking the limit $\delta \to 0$ in Eq. (11) in the main text, $\mathcal{F}^{(\mathrm{im})}$ coincides with $\mathcal{F}[\Lambda_{\mathrm{dep}}[\rho(\theta)]] = (2\mathsf{p}-1)^2$. This is demonstrated by the blue dot in Suppl. Fig. 2 for $\mathsf{p} = \mathsf{q} = 0.9$.

For asymmetric mixing $\mathsf{p} \neq \mathsf{q}$, however, one can show that for a wide range of p and q any channel Λ satisfying Eq. (16) cannot exhibit phase-covariance. In particular, Λ can only be phase-covariant when there exists $\phi \in [0,2\pi)$ such that

$$|\cos\phi| \geq |\delta| \quad \text{and} \quad 1 \geq \frac{4\eta^2}{\sin^2\phi} + \frac{\delta^2}{\cos^2\phi}, \qquad (49)$$

where $\eta := \mathsf{p}+\mathsf{q}-1$ and $\delta := \mathsf{p}-\mathsf{q}$ as in the main text, and the above conditions originate from the CP-constraints on any phase-covariant channel (see e.g. Ref. [8]). Nonetheless, note that if conditions (49) can be fulfilled for a given pair of $\mathsf{p} \neq \mathsf{q}$, there may exist more than one valid phase-covariant conjugate map $\Lambda^\dagger$ in Eq. (16), i.e. there can be multiple solutions for ϕ satisfying the inequalities (49).

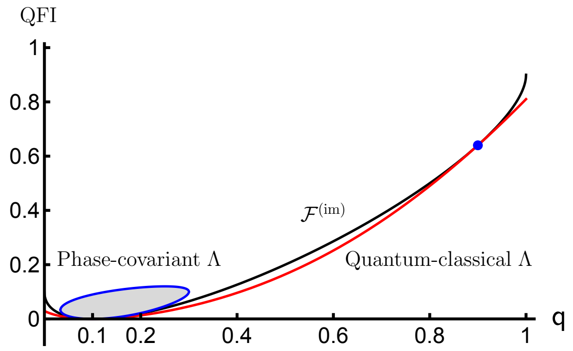

Supplementary Figure 2. **Imperfect QFI $\mathcal{F}^{(\mathrm{im})}$ vs G-covariance-based bounds $\mathcal{F}[\Lambda[\rho(\theta)]]$ for a single qubit** ($N = 1$), when estimating the phase encoded onto the input state $|+\rangle$, with the projective measurement affected by asymmetric bit-flip errors parametrised by $\mathsf{p} = 0.9$ and q (horizontal axis). For any phase-covariant channel Λ that commutes with the U(1)-representation $\mathcal{V}_g \sim \{\mathrm{e}^{\mathrm{i}g\sigma_z}\}$—and, hence, the optimal unitary $\mathcal{V}_{\mathrm{opt}}$, see Supplementary Note 3—the G-covariance condition (22) applies and the Observation 1 implies that $\mathcal{F}^{(\mathrm{im})} \leq \mathcal{F}[\Lambda[\rho(\theta)]]$. Hence, the QFI (50) applicable to all phase-covariant maps Λ satisfying Eq. (16) yields upper bounds marked with the *blue solid line* encircling the shaded region, as well as the *blue dot* at $\mathsf{p} = \mathsf{q}$, that consistently lie above the *black solid line* denoting the true $\mathcal{F}^{(\mathrm{im})}$, as given by Eq. (11) in the main text. Still, the phase-covariance can be ensured only for a narrow range of q—beyond which any Λ that fulfils Eq. (16) may yield $\mathcal{F}[\Lambda[\rho(\theta)]] < \mathcal{F}^{(\mathrm{im})}$, e.g. the quantum-classical channel defined in Eq. (17) that leads to the *red curve* above. However, we also observe that the channel defined in Eq. (18) yields $\mathcal{F}[\Lambda[\rho(\theta)]] = \mathcal{F}^{(\mathrm{im})}$ despite not being phase-covariant (the resulting QFI coincides with the *black line*).

In general, for any phase-covariant Λ satisfying Eq. (16), $\mathcal{F}^{(\mathrm{im})} \leq \mathcal{F}[\Lambda[\rho(\theta)]]$ holds with $\mathcal{F}^{(\mathrm{im})}$ given by Eq. (11) in the main text, while

$$\mathcal{F}[\Lambda[\rho(\theta)]] = \frac{\eta^2}{\sin^2\phi} \qquad (50)$$

depending on the choice of ϕ satisfying constraints (49). In Suppl. Fig. 2, we mark such a region by grey shading (blue oval boundary) with all values of $\mathcal{F}[\Lambda[\rho(\theta)]]$ in Eq. (50) lying consistently above the true $\mathcal{F}^{(\mathrm{im})}$ (black line). Note that this is possible *only* for relatively small

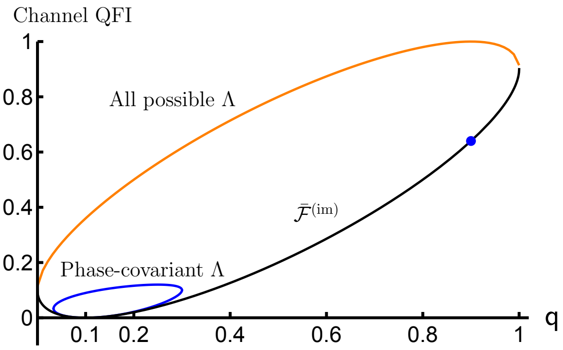

Supplementary Figure 3. **Imperfect channel QFI $\bar{\mathcal{F}}^{(\mathrm{im})}$ vs G-covariance-based bounds $\bar{\mathcal{F}}[(\Lambda\circ\mathcal{E}_\theta)]$ in phase sensing with a single qubit** ($N=1$), with the projective measurement affected by asymmetric bit-flip errors parametrised by $\mathsf{p}=0.9$ and q (horizontal axis). As the phase encoding $\mathcal{E}_\theta \sim \{\mathrm{e}^{\mathrm{i}\theta\sigma_z/2}\}$ commutes with the U(1)-representation $\mathcal{V}_g \sim \{\mathrm{e}^{\mathrm{i}g\sigma_z}\}$—and, hence, the optimal unitary $\mathcal{V}_{\mathrm{opt}}$, see Supplementary Note 3—the G-covariance condition (26) is satisfied and the Observation 2 implies that $\bar{\mathcal{F}}^{(\mathrm{im})} \leq \bar{\mathcal{F}}[(\Lambda\circ\mathcal{E}_\theta)]$ for *any* choice of Λ satisfying Eq. (16), For any such Λ, the upper bound can then always be efficiently computed by resorting to the 'seesaw' method via Eq. (42). This is illustrated by choosing Λ to represent phase-covariant channels (*blue line*, see Eq. (50), and *blue dot* at $\mathsf{q}=\mathsf{p}$), the quantum-classical channel (17) and the channel (18), for both of which we observe $\bar{\mathcal{F}}[(\Lambda\circ\mathcal{E}_\theta)] = \bar{\mathcal{F}}^{(\mathrm{im})}$ (coinciding with the *black line* depicting the true imperfect channel QFI). However, by resorting to Eq. (48), we also perform optimisation over *all possible* conjugate-map decompositions, Λ satisfying Eq. (16), in order to observe that the so-determined $\bar{\mathcal{F}}[(\Lambda\circ\mathcal{E}_\theta)]$ (*orange line*) is not even convex in q, being tight only at $\mathsf{q}=0,1$.

q (apart from special $\mathsf{q}=\mathsf{p}$), given the value of $\mathsf{p}=0.9$ chosen.

On the contrary, within the range of $\mathsf{p}\neq\mathsf{q}$ in Suppl. Fig. 2 that yield imperfect measurements $\mathcal{M}$ whose valid conjugate-map decompositions in Eq. (16) may *not* exhibit phase-covariance (or more generally, G-covariance)—range of q without any solution marked in blue—the Observation 1 is not applicable and the upper bound (23) can no longer be taken for granted. Indeed, as demonstrated by the red solid line in Suppl. Fig. 2 for the quantum-classical channel (15), which yields a valid conjugate-map decomposition for any q, its QFI no longer upper-bounds the imperfect QFI with, in fact, $\mathcal{F}^{(\mathrm{im})} \geq \mathcal{F}[\Lambda[\rho(\theta)]]$. It is so, as $\mathcal{F}[\Lambda[\rho(\theta)]]$ equals then the classical FI for the imperfect measurement $\mathcal{M}$ with no control ($\mathcal{V}_{\vec{\phi}}=\mathbb{1}$) and, hence, by definition is always smaller or equal to $\mathcal{F}^{(\mathrm{im})}$. Interestingly, in this particular qubit example with asymmetric bit-flip noise, $\mathcal{F}[\Lambda[\rho(\theta)]]$ coincides with the imperfect QFI, $\mathcal{F}^{(\mathrm{im})}$, when Λ is chosen to be the channel defined in Eq. (18).

The same exemplary model can also be used to illustrate the applicability of the Observation 2 discussed in Supplementary Note 4 A, which applies rather to the *imperfect channel QFI*, $\bar{\mathcal{F}}^{(\mathrm{im})}$, incorporating maximisation over the input states in Eqs. (41) and (42). As explained in the main text, see particularly Fig. 2 therein, when allowing for arbitrary control any input state lying on the equator of the Bloch sphere is optimal, so that $\bar{\mathcal{F}}^{(\mathrm{im})} = \mathcal{F}^{(\mathrm{im})}$ for the input $|+\rangle$ and the corresponding curve (*black solid line*) in Suppl. Fig. 3 is just the same as the one for $\mathcal{F}^{(\mathrm{im})}$ in Suppl. Fig. 2 (given by Eq. (11) in the main text). Now, as the phase encoding $\mathrm{e}^{\mathrm{i}\theta\sigma_z/2}$ commutes with the group of control unitaries $\mathrm{e}^{\mathrm{i}\varphi\sigma_z}$ for any φ, the G-covariance condition (26) is satisfied instead, and at the level of the imperfect channel QFI the inequality $\bar{\mathcal{F}}^{(\mathrm{im})} \leq \bar{\mathcal{F}}[(\Lambda\circ\mathcal{E}_\theta)]$ holds for *any* channel Λ satisfying Eq. (16).

In Fig. 3, with help of the 'seesaw' algorithm introduced in Supplementary Note 4 C, we compute $\bar{\mathcal{F}}[(\Lambda\circ\mathcal{E}_\theta)]$ in Eq. (45) with Λ representing: phase-covariant channels, quantum-classical channel (17) and the channel (18). In the first case, as any state from the equator is still optimal thanks to the phase-covariance property, we can equivalently utilise Eq. (50) and recover the same upper bounds as in Suppl. Fig. 2 (*blue oval and dot*). However, in the latter two cases, we find the resulting upper bounds to coincide with the imperfect channel QFI, i.e. $\bar{\mathcal{F}}^{(\mathrm{im})} = \bar{\mathcal{F}}[(\Lambda\circ\mathcal{E}_\theta)]$ with Λ of Eq. (17) or Eq. (18). Note that this contrasts the case of quantum-classical channel in Suppl. Fig. 2, which thanks to the optimisation of the input state (for each value of q) yields now a valid "upper-bound"—actually reproducing the exact $\bar{\mathcal{F}}^{(\mathrm{im})}$.

Furthermore, we resort to the generalisation of the 'seesaw' algorithm that includes maximisation over *all possible* conjugate-map decompositions of the imperfect measurement, as discussed in Supplementary Note 4 D. In this way, we obtain $\bar{\mathcal{F}}[(\Lambda\circ\mathcal{E}_\theta)]$ maximised over all Λ satisfying Eq. (16) for each bit-flip error values p and q—*orange line* in Suppl. Fig. 3. We observe that such an upper is not even convex as the true imperfect channel QFI, $\bar{\mathcal{F}}^{(\mathrm{im})}$ (*black line*) and, hence, tight only at the extremal values of $\mathsf{q}=0$ or 1.

F. *Example 2: $N=2$ qubits phase sensing with bit-flip noise*

In this example, we demonstrate a scenario where now the G-covariance condition (26) is not satisfied and, hence, also Observation 2 is not applicable. We consider generalisation of the above phase-sensing example to the case of two ($N=2$) qubits, in which each qubit probe state undergoes the same encoding and imperfect measurement. For our purposes, we consider only the case of symmetric ($\mathsf{p}=\mathsf{q}$) bit-flip as the detection noise, as it is sufficient to explore the possibility of the optimal input probe state now being entangled and, crucially, the control operation $\mathcal{V}_{\vec{\phi}}$ now acting globally on both qubits,

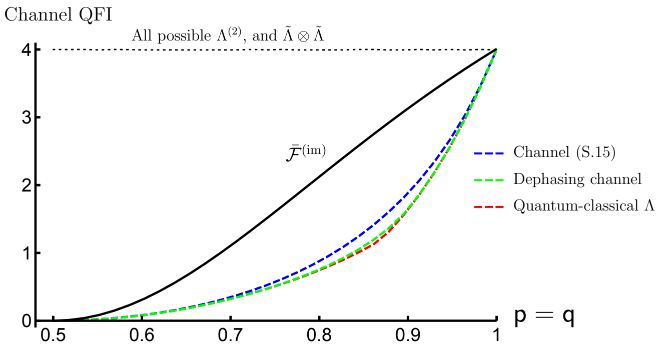

Supplementary Figure 4. **Imperfect channel QFI $\bar{\mathcal{F}}^{(\mathrm{im})}$ vs channel QFIs $\bar{\mathcal{F}}[(\Lambda\circ\mathcal{E}_\theta)^{\otimes 2}]$ in phase sensing with two qubits** ($N=2$), as a function of the probability for symmetric bit-flip errors, $\mathsf{p}=\mathsf{q}\geq 0.5$, affecting qubit projective-measurement outcomes, where Λ are some valid conjugate-map decompositions (16) of the resulting (local) imperfect measurements. The *black solid line* depicts the true imperfect channel QFI, $\bar{\mathcal{F}}^{(\mathrm{im})}$, obtained by determining numerically for each $\mathsf{p}=\mathsf{q}$ the optimal control unitary, $\mathcal{V}_{\mathrm{opt}}$, which now crucially acts globally on both qubits and may not commute with neither the conjugate-map decompositions considered, $\Lambda^{\otimes 2}$, nor the encoding, $\mathcal{E}_\theta^{\otimes 2}$. As result, both the G-covariance conditions (22) and (26) need not be satisfied. This is confirmed by observing that Eq. (27) is explicitly invalidated, i.e. $\bar{\mathcal{F}}^{(\mathrm{im})}\not\leq\bar{\mathcal{F}}[(\Lambda\circ\mathcal{E}_\theta)^{\otimes 2}]$, for conjugate-map decompositions with Λ corresponding to either: the dephasing channel (*green*), quantum-classical channel (17) (*red*) or the channel defined in Eq. (18) (*blue*)—observe all the corresponding curves lying below the one of $\bar{\mathcal{F}}^{(\mathrm{im})}$. On the other hand, the 'seesaw' algorithm allows us to find also the local and global channels $\tilde{\Lambda}^{\otimes 2}$ and $\Lambda^{(2)}$, respectively, that constitute valid conjugate-map decompositions (16) at each $\mathsf{p}=\mathsf{q}$ but are *uninformative*—yield $\bar{\mathcal{F}}[(\tilde{\Lambda}\circ\mathcal{E}_\theta)^{\otimes 2}]=\bar{\mathcal{F}}[\tilde{\Lambda}^{(2)}\circ\mathcal{E}_\theta^{\otimes 2}]=\bar{\mathcal{F}}[\mathcal{E}_\theta^{\otimes 2}]=4$ (*black dots*), i.e. the perfect quantum channel QFI. This emphasises further that, already for $N=2$, results of quantum metrology with noisy encoding may not be directly used to estimate performance with imperfect measurements.

i.e. constituting an entangling gate.

First, from Lemma 1, we know that the imperfect measurement does not change the optimal input state, which is thus: $|\Phi_+\rangle=(|00\rangle+|11\rangle)/\sqrt{2}$. Then, one may prove that the optimal two-qubit control unitary must take the form (see also the discussion below in Supplementary Note 5): $\mathcal{V}_{\mathrm{opt}}\sim\{V_{\mathrm{opt}}=W\mathrm{e}^{\mathrm{i}\varphi_{\mathrm{opt}}(\sigma_z\otimes\mathbb{1}+\mathbb{1}\otimes\sigma_z)}\}$ for some optimal real coefficient φ_{opt}, where W is the unitary that transforms the Bell basis onto the product basis $\{|++\rangle,|+-\rangle,|-+\rangle,|--\rangle\}$. That is, $W=|++\rangle\langle\Phi_+|+|+-\rangle\langle\Psi_+|+|-+\rangle\langle\Psi_-|+|--\rangle\langle\Phi_-|$, where $|\Phi_\pm\rangle=(|00\rangle\pm|11\rangle)/\sqrt{2}$ and $|\Psi_\pm\rangle=(|01\rangle\pm|10\rangle)/\sqrt{2}$. However, one can also verify that unitaries of such a form generally do not commute with the encoding $\mathcal{E}_\theta^{\otimes 2}$, so there is no straightforward identification that the G-covariance condition (26) can be satisfied. In fact, since satisfying the G-covariance condition (26) implies that Eq. (27) must hold for any Λ, finding an example of Λ that violates Eq. (27) and, hence, the Observation 2, would imply that (26) *cannot* be fulfilled.

We illustrate this explicitly in Suppl. Fig. 4, where now—contrastingly to the single-qubit case—all the channel QFIs $\bar{\mathcal{F}}[(\Lambda\circ\mathcal{E}_\theta)^{\otimes 2}]$ evaluated for the conjugate-map decompositions (16) with Λ representing: the dephasing channel (*green dashed line*), the quantum-classical channel (17) (*red dashed line*) and the channel (18) (*blue dashed line*); lie below the true imperfect channel QFI, $\bar{\mathcal{F}}^{(\mathrm{im})}$, for any $\frac{1}{2}<\mathsf{p}=\mathsf{q}<1$. Hence, all these three examples invalidate the upper bounds (23) and (27), and prove that at any such $\mathsf{p}=\mathsf{q}$, $\mathcal{V}_{\mathrm{opt}}$ must not commute with neither $\Lambda^{\otimes 2}$ nor $\mathcal{E}_\theta^{\otimes 2}$, i.e. none of the G-covariance conditions (22) and (26), respectively, may hold.

We evaluate the aforementioned channel QFIs in Suppl. Fig. 4 for the three types of conjugate-map decompositions by resorting to the 'seesaw' algorithm described in Supplementary Note 4 C. However, one should also recall from Suppl. Fig. 3 (see the maximum of the *orange line*) that in the single-qubit case there exists $\tilde{\Lambda}$ satisfying (16) at $\mathsf{p}=\mathsf{q}$, such that $\bar{\mathcal{F}}[(\tilde{\Lambda}\circ\mathcal{E}_\theta)]=\bar{\mathcal{F}}[\mathcal{E}_\theta]=1$, i.e. its corresponding channel QFI actually equals the perfect channel QFI and, hence, is useless in estimating the impact of the detection (bit-flip) noise. We verify that such a conjugate-map decomposition remains *uninformative* also in the two-qubit scenario, in which it leads to $\bar{\mathcal{F}}[(\tilde{\Lambda}\circ\mathcal{E}_\theta)^{\otimes 2}]=\bar{\mathcal{F}}[\mathcal{E}_\theta^{\otimes 2}]=4$ for any $\mathsf{p}=\mathsf{q}$—see the *black dotted line* in Suppl. Fig. 4. For consistency, by resorting to the 'seesaw' algorithm of Supplementary Note 4 D that includes maximisation over conjugate-map decompositions, we also verify that there exists a global $\Lambda^{(2)}$ that satisfies the condition (16) for $N=2$, i.e. $\mathcal{M}^{\otimes 2}=\Lambda^{(2)\dagger}[\Pi^{\otimes 2}]$, and similarly leads to $\bar{\mathcal{F}}[\tilde{\Lambda}^{(2)}\circ\mathcal{E}_\theta^{\otimes 2}]=\bar{\mathcal{F}}[\mathcal{E}_\theta^{\otimes 2}]=4$. Interestingly, we observe that neither $\tilde{\Lambda}^{\otimes 2}$ nor $\Lambda^{(2)}$ commute in general with $\mathcal{V}_{\mathrm{opt}}$, so that G-covariance condition (22) still does not apply—making the connection between metrological protocols with imperfect (local) measurements and the "standard" setting of quantum metrology with noisy encoding on multiple probes even less apparent.

Supplementary Note 5. Proof of Theorem 1 and convergence rate to the perfect QFI

As the results and expressions derived in Lemma 1 are independent of the number of probes, it follows that Equations (1-4) hold still. Applying specifically to the multi-probe scenario discussed in the main text with the structure of measurement as $\mathcal{M}^{\otimes N}$, i.e. each probe is still measured independently but in an imperfect manner, Eqs. (3) and (4) read

$$F_N = 4\,\gamma(\vec{\Phi},\psi^N(\theta))\,\langle\partial_\theta\psi_\perp^N(\theta)|\partial_\theta\psi_\perp^N(\theta)\rangle, \qquad (51)$$

with

$$\gamma(\vec{\Phi},\psi^N(\theta)) := \frac{1}{4}\sum_{\boldsymbol{x}} \frac{[\langle\psi_\perp^N(\theta)|V_{\vec{\Phi}}^\dagger M_{\boldsymbol{x}} V_{\vec{\Phi}}|\psi^N(\theta)\rangle + \text{c.c.}]^2}{\langle\psi^N(\theta)|V_{\vec{\Phi}}^\dagger M_{\boldsymbol{x}} V_{\vec{\Phi}}|\psi^N(\theta)\rangle}, \quad (52)$$

where now $\boldsymbol{x} = (x_1, x_2, \cdots, x_N)$, with $\mathcal{M}^{\otimes N} \sim \{M_{\boldsymbol{x}}\}$,

$$M_{\boldsymbol{x}} = M_{x_1} \otimes M_{x_2} \otimes \cdots \otimes M_{x_N}, \quad (53)$$

where M_{x_ℓ} is the noisy measurement operator for outcome x_ℓ for the ℓ-th probe.

A lower bound on $\gamma(\vec{\Phi},\psi^N(\theta))$, and hence on F_N, for any given choice of $V_{\vec{\Phi}}$ can be constructed as follows. Define $|\psi_\pm^N(\theta)\rangle := \frac{1}{\sqrt{2}}\Big(|\psi^N(\theta)\rangle \pm |\psi_\perp^N(\theta)\rangle\Big)$, and $p_\pm(\boldsymbol{x}) := \langle\psi_\pm^N(\theta)|V_{\vec{\Phi}}^\dagger M_{\boldsymbol{x}} V_{\vec{\Phi}}|\psi_\pm^N(\theta)\rangle$, where in order not to overload the notation, we have kept the θ and $\vec{\Phi}$ dependence implicit. Then, the numerator of $4\gamma(\vec{\Phi},\psi^N(\theta))$ is

$$\big(p_+(\boldsymbol{x}) - p_-(\boldsymbol{x})\big)^2, \quad (54)$$

while the denominator is

$$\frac{1}{2}\Big(p_+(\boldsymbol{x}) + p_-(\boldsymbol{x}) + \langle\psi_-^N|V_{\vec{\Phi}}^\dagger M_{\boldsymbol{x}} V_{\vec{\Phi}}|\psi_+^N\rangle + \langle\psi_+^N|V_{\vec{\Phi}}^\dagger M_{\boldsymbol{x}} V_{\vec{\Phi}}|\psi_-^N\rangle\Big), \quad (55)$$

which by the Cauchy-Schwartz inequality is smaller or equal to

$$\frac{1}{2}\Big(\sqrt{p_+(\boldsymbol{x})} + \sqrt{p_-(\boldsymbol{x})}\Big)^2. \quad (56)$$

Hence, we have

$$\begin{aligned}\gamma(\vec{\Phi},\psi^N(\theta)) &\geq \frac{1}{2}\sum_{\boldsymbol{x}}\Big(\sqrt{p_+(\boldsymbol{x})} - \sqrt{p_-(\boldsymbol{x})}\Big)^2 \\ &= \Big(1 - \sum_{\boldsymbol{x}}\sqrt{p_+(\boldsymbol{x})p_-(\boldsymbol{x})}\Big). \end{aligned} \quad (57)$$

Let us now denote:

$$\begin{aligned} V_{\vec{\Phi}}|\psi_+^N(\theta)\rangle &= |\zeta^N\rangle, \\ V_{\vec{\Phi}}|\psi_-^N(\theta)\rangle &= |\zeta_\perp^N\rangle, \end{aligned} \quad (58)$$

and note that $p_\pm(\boldsymbol{x})$ depends solely on our choice of $|\zeta^N\rangle, |\zeta_\perp^N\rangle$. To attain the lower bound in Eq. (17) of Thm. 1, consider a specific choice of $|\zeta^N\rangle, |\zeta_\perp^N\rangle$ (obtained by a suitable $V_{\vec{\Phi}}$) :

$$\begin{aligned} |\zeta^N\rangle &= |\zeta\rangle^{\otimes N}, \\ |\zeta_\perp^N\rangle &= |\zeta_\perp\rangle^{\otimes N} \end{aligned} \quad (59)$$

for some orthogonal single-qudit states $\{|\zeta\rangle, |\zeta_\perp\rangle\}$. Then, with $p_+(x) := \langle\zeta|M_x|\zeta\rangle, p_-(x) := \langle\zeta_\perp|M_x|\zeta_\perp\rangle$, and $c := \sum_x \sqrt{p_+(x)p_-(x)}$, we have $p_\pm(\boldsymbol{x}) = \prod_{j=1}^N p_\pm(x_j)$, and thus

$$\sum_{\boldsymbol{x}}\sqrt{p_+(\boldsymbol{x})p_-(\boldsymbol{x})} = c^N. \quad (60)$$

Evidently, we have $0 \leq c \leq 1$, and so we complete the proof of Thm 1. ■

A few remarks are in order: Given a specific choice of $\{|\zeta\rangle^{\otimes N}, |\zeta_\perp\rangle^{\otimes N}\}$ (equivalently $V_{\vec{\Phi}}$ and $p_\pm(\boldsymbol{x})$}) the FI converges to the perfect QFI exponentially fast with an approximate prefactor (see Eqs. (57) and (60)):

$$\begin{aligned} 1 - c^N &= 1 - \Big(\sum_x\sqrt{p_+(x)\,p_-(x)}\Big)^N \\ &= 1 - \Big(\sum_{\boldsymbol{x}}\sqrt{p_+(\boldsymbol{x})\,p_-(\boldsymbol{x})}\Big). \end{aligned} \quad (61)$$

Note that this prefactor is the Hellinger distance, $H(p_+(\boldsymbol{x}), p_-(\boldsymbol{x}))$, between the distributions $p_+(\boldsymbol{x})$ and $p_-(\boldsymbol{x})$ [12], while the convergence rate χ ($c \equiv \mathrm{e}^{-\chi}$) reads

$$\begin{aligned} \chi &= -\log\Big(\sum_x\sqrt{p_+(x)\,p_-(x)}\Big) \\ &= -\frac{1}{N}\log\Big(1 - H\big(p_+(\boldsymbol{x}), p_-(\boldsymbol{x})\big)\Big). \end{aligned} \quad (62)$$

For very close distributions it can be observed that the convergence rate is equal to the Fisher metric: $\sum_x \frac{(p_+(x)-p_-(x))^2}{8p_+(x)}$.

As $N \to \infty$, generally we can use the central limit theorem to approximate $H(p_+(\boldsymbol{x}), p_-(\boldsymbol{x}))$ as the Hellinger distance between two Gaussian distributions. This would then imply a convergence rate of: $\frac{1}{4}\frac{(\mu_+-\mu_-)^2}{\sigma_+^2+\sigma_-^2}$, where $\mu_\pm, \sigma_\pm$ are the average and standard deviation of $p_\pm(x)$ respectively, which of course depends on our choice of $|\zeta\rangle, |\zeta_\perp\rangle$.

We can thus apply this analysis to obtain the convergence rate for specific cases. For Poissonian channel (with coefficients $\lambda_{|0\rangle}, \lambda_{|1\rangle}$), such as in NV centres, we find a convergence rate of: $\frac{1}{2}\left(\sqrt{\lambda_{|0\rangle}} - \sqrt{\lambda_{|1\rangle}}\right)^2$. This convergence rate is achieved by taking $|\zeta^N\rangle, |\zeta_\perp^N\rangle$ to be $|0\rangle^{\otimes N}, |1\rangle^{\otimes N}$ (similar convergence rate is obtained by taking them to be any superposition of $|0\rangle^{\otimes N}, |1\rangle^{\otimes N}$). This implies that for realistic experimental values of NV centres, $\lambda_{|0\rangle} = 0.1, \lambda_{|1\rangle} = 0.07$ (without the use of nuclear spins as memory), the number of probes that obtains 95% of the perfect QFI would be ~ 2000. For a binary asymmetric bit-flip channel with probabilities p, q, we find a convergence rate of: $\frac{1}{4}\frac{(\mathsf{p}+\mathsf{q}-1)^2}{\mathsf{p}(1-\mathsf{p})+\mathsf{q}(1-\mathsf{q})}$, given a similar choice of $|\zeta\rangle = |0\rangle^{\otimes N}, |\zeta_\perp\rangle = |1\rangle^{\otimes N}$. As a side note, we may also compute c directly in this case, and it can be verified that indeed $c^N = \left(\sqrt{\mathsf{p}(1-\mathsf{q})} + \sqrt{\mathsf{q}(1-\mathsf{p})}\right)^N \approx \exp(-\chi N)$ with the said χ.

A note on optimality: Let us justify our choice of unitary, namely the choice of $|\zeta^N\rangle = |0\rangle^{\otimes N}, |\zeta_\perp^N\rangle = |1\rangle^{\otimes N}$. Given the approximated value of the convergence rate, Eq. (62), we claim that this choice yields the optimal convergence rate. That is, we would like to choose $|\zeta^N\rangle, |\zeta_\perp^N\rangle$ that maximize the Hellinger distance between $\{p_+(\boldsymbol{x}) = \langle\zeta^N|M_{\boldsymbol{x}}|\zeta^N\rangle\}_{\boldsymbol{x}}, \{p_-(\boldsymbol{x}) = \langle\zeta_\perp^N|M_{\boldsymbol{x}}|\zeta_\perp^N\rangle\}_{\boldsymbol{x}}$. As before, we focus on the classical noise channel, namely commuting M_x: $M_x = \sum_i p(x|i)\,\Pi_i$. Observe that:

- The Hellinger distance is convex in the probability distributions: $H(\lambda p_1 + (1-\lambda)p_2, q) \leq \lambda H(p_1, q) + (1-\lambda) H(p_2, q)$.
- Let $\{|j\rangle\}_j$ be the common eigenbasis of $\{M_x\}_x$, then given $|\zeta\rangle = \sqrt{\lambda}|j_1\rangle + \sqrt{1-\lambda}|j_2\rangle$: $\langle\zeta|M_x|\zeta\rangle = \lambda\langle j_1|M_x|j_1\rangle + (1-\lambda)\langle j_2|M_x|j_2\rangle$. In words: taking superpositions of the eigenstates leads to convex combinations of the probabilities.

The above two observations imply that the maximal Hellinger distance is achieved by taking $|\zeta^N\rangle, |\zeta_\perp^N\rangle$ to be elements in the common eigenbasis (and not superpositions of them). Hence, we just need to find the two basis states that yield maximal Hellinger distance. For N qudits, we thus need to find the two states $|j_1\rangle, |j_2\rangle$ with maximal Hellinger distance out of the d-dimensional eigenbasis, and then the optimal choice of $|\zeta^N\rangle, |\zeta_\perp^N\rangle$ would be $|j_1\rangle^{\otimes N}, |j_2\rangle^{\otimes N}$. For the case of NV centres with the local projective measurement $\{\Pi_1 = |0\rangle\langle 0|, \Pi_2 = |1\rangle\langle 1|\}$ for each of the N NV centres, this immediately implies that the optimal $|\zeta^N\rangle, |\zeta_\perp^N\rangle$ are $|0\rangle^{\otimes N}, |1\rangle^{\otimes N}$.

In fact this intuition together with some numerical evidence leads us to conjecture that for any classical noise channel, $M_x = \sum_i p(x|i)\,\Pi_i$ that is applied independently on each of the N probes, the optimal $|\zeta^N\rangle, |\zeta_\perp^N\rangle$ take the form of "cat states":

$$|\zeta^N\rangle = \cos(\theta)\,|j\rangle^{\otimes N} + \sin(\theta)\,|k\rangle^{\otimes N},$$
$$|\zeta_\perp^N\rangle = -\sin(\theta)\,|j\rangle^{\otimes N} + \cos(\theta)\,|k\rangle^{\otimes N},$$

where $|j\rangle, |k\rangle$ can be found numerically for just a single probe, and θ depends on N and should be found numerically. In particular for N qubits we connjecture that the optimal $|\zeta^N\rangle, |\zeta_\perp^N\rangle$ take the form of $\cos(\theta)\,|0\rangle^{\otimes N} + \sin(\theta)\,|1\rangle^{\otimes N}, -\sin(\theta)\,|0\rangle^{\otimes N} + \cos(\theta)\,|1\rangle^{\otimes N}$.

Supplementary Note 6. Proof of Lemma 2

With the encoded state $\rho_r^N(\theta) = r\,\psi^N(\theta) + (1-r)\mathbb{1}_{d^N}/d^N$, where $\psi^N(\theta) = |\psi^N(\theta)\rangle\langle\psi^N(\theta)|$, similar to the proof of Thm. 1, it is straightforward to establish that the FI can be written as

$$F_N = 4\langle\partial_\theta\psi_\perp^N(\theta)|\partial_\theta\psi_\perp^N(\theta)\rangle\gamma_r(V_{\vec{\Phi}}, |\psi^N(\theta)\rangle), \tag{63}$$

where

$$\gamma_r(\vec{\Phi}, \psi^N(\theta)) = \frac{1}{4}\sum_{\boldsymbol{x}} \frac{r^2[\langle\psi_\perp^N(\theta)|V_{\vec{\Phi}}^\dagger M_{\boldsymbol{x}} V_{\vec{\Phi}}|\psi^N(\theta)\rangle + \text{c.c.}]^2}{r\langle\psi^N(\theta)|V_{\vec{\Phi}}^\dagger M_{\boldsymbol{x}} V_{\vec{\Phi}}|\psi^N(\theta)\rangle + (1-r)p'(\boldsymbol{x})}, \tag{64}$$

with $p'(\boldsymbol{x}) := \mathrm{Tr}\{M_{\boldsymbol{x}}\}/d^N$. Note that $p'(\boldsymbol{x})$ is a legit probability distribution, i.e., $p'(\boldsymbol{x}) \geq 0\,\forall\boldsymbol{x}$, and $\sum_{\boldsymbol{x}} p'(\boldsymbol{x}) = 1$. Moreover, evidently $\gamma_r(\vec{\Phi}, \psi^N(\theta)) \leq r\gamma(\vec{\Phi}, \psi^N(\theta)) \leq r$, and so we arrive at an upper bound $F_N \leq 4r\langle\partial_\theta\psi_\perp^N(\theta)|\partial_\theta\psi_\perp^N(\theta)\rangle = r\mathcal{F}[\psi^N(\theta)]$.

Now, using essentially the same observation and notation leading to Eqs. (54-56), the following inequality holds:

$$\gamma_r(\vec{\Phi}, \psi^N(\theta)) \geq \frac{1}{4}\sum_{\boldsymbol{x}} \frac{r^2\big(p_+(\boldsymbol{x}) - p_-(\boldsymbol{x})\big)^2}{\frac{r}{2}\Big(\sqrt{p_+(\boldsymbol{x})} + \sqrt{p_-(\boldsymbol{x})}\Big)^2 + (1-r)p'(\boldsymbol{x})}. \tag{65}$$

Consider then three different ways of grouping all the $\boldsymbol{x}$. First, consider two sets, A_+ and B_+, defined as

$$A_+ := \{\boldsymbol{x}\,|\,(1-r)p'(\boldsymbol{x}) \leq rp_+(\boldsymbol{x})\},$$
$$B_+ := \{\boldsymbol{x}\,|\,(1-r)p'(\boldsymbol{x}) > rp_+(\boldsymbol{x})\}. \tag{66}$$

Similarly, define the sets

$$A_- := \{\boldsymbol{x}\,|\,(1-r)p'(\boldsymbol{x}) \leq rp_-(\boldsymbol{x})\},$$
$$B_- := \{\boldsymbol{x}\,|\,(1-r)p'(\boldsymbol{x}) > rp_-(\boldsymbol{x})\}, \tag{67}$$

as well as

$$A := \{\boldsymbol{x}\,|\,(1-r)p'(\boldsymbol{x}) \leq r\max\big(p_+(\boldsymbol{x}), p_-(\boldsymbol{x})\big)\},$$
$$B := \{\boldsymbol{x}\,|\,(1-r)p'(\boldsymbol{x}) > r\max\big(p_+(\boldsymbol{x}), p_-(\boldsymbol{x})\big)\}. \tag{68}$$

Evidently, $A_+ \cup A_- = A$, and $B_+ \cap B_- = B$. Morever, define

$$\epsilon_\pm := \sum_{\boldsymbol{x}} \min\big(rp_\pm(\boldsymbol{x}), (1-r)p'(\boldsymbol{x})\big), \tag{69}$$

which can be interpreted as the minimal error in discriminating the two probability distributions $\{p_\pm(\boldsymbol{x})\}$ and $\{p'(\boldsymbol{x})\}$ with prior r and $1-r$, respectively. Thus,

$$\begin{aligned}\epsilon_+ + \epsilon_- =& \Big(\sum_{\boldsymbol{x}\in A_+} + \sum_{\boldsymbol{x}\in A_-}\Big)(1-r)p'(\boldsymbol{x}) \\ &+ \sum_{\boldsymbol{x}\in B_+} rp_+(\boldsymbol{x}) + \sum_{\boldsymbol{x}\in B_-} rp_-(\boldsymbol{x}) \\ \geq& \sum_{\boldsymbol{x}\in A}(1-r)p'(\boldsymbol{x}) + \\ &+ \sum_{\boldsymbol{x}\in B} r\max\big(p_+(\boldsymbol{x}), p_-(\boldsymbol{x})\big).\end{aligned} \tag{70}$$

Then, for the r.h.s. of Eq. (65), we shall evaluate the sum over all $\boldsymbol{x}$ into A and B respectively. For $\boldsymbol{x} \in A$, using the identity $(1+x)^{-1} \geq 1-x$ for any $(1+x) \in \mathbb{R}_+$, we get

$$\sum_{\boldsymbol{x}\in A}(\cdots) \geq 2r \sum_{\boldsymbol{x}\in A}\left(\sqrt{p_+(\boldsymbol{x})} - \sqrt{p_-(\boldsymbol{x})}\right)^2 - 4\sum_{\boldsymbol{x}\in A}(1-r)p'(\boldsymbol{x}). \tag{71}$$

Meanwhile, for $\boldsymbol{x}$ in the set B, observe that

$$\begin{aligned} &2r\left(\sqrt{p_+(\boldsymbol{x})} - \sqrt{p_-(\boldsymbol{x})}\right)^2 \\ &\leq 2r\big(p_+(\boldsymbol{x}) + p_-(\boldsymbol{x})\big) \\ &\leq 4r \max\big(p_+(\boldsymbol{x}), p_-(\boldsymbol{x})\big). \end{aligned} \tag{72}$$

Hence, we have

$$\sum_{\boldsymbol{x}\in B}(\cdots) \geq \sum_{\boldsymbol{x}\in B} 2r\left(\sqrt{p_+(\boldsymbol{x})} - \sqrt{p_-(\boldsymbol{x})}\right)^2 - 4r\sum_{\boldsymbol{x}\in B}\max\big(p_+(\boldsymbol{x}), p_-(\boldsymbol{x})\big). \tag{73}$$

Combining Eqs. (65, 70, 71, 73), we obtain

$$\begin{aligned} &\gamma_r(\vec{\Phi}, \psi^N(\theta)) \\ &\geq \frac{1}{2} r \sum_{\boldsymbol{x}}\left(\sqrt{p_+(\boldsymbol{x})} - \sqrt{p_-(\boldsymbol{x})}\right)^2 - \epsilon_+ - \epsilon_- \\ &= r\left(1 - \sum_{\boldsymbol{x}}\sqrt{p_+(\boldsymbol{x})p_-(\boldsymbol{x})}\right) - \epsilon_+ - \epsilon_-. \end{aligned} \tag{74}$$

Finally, upon choosing $V_{\vec{\Phi}}$ as in Eq. (59), we have $F_N(V_{\vec{\Phi}}) \geq \mathcal{F}[\psi^N(\theta)]\big(r(1-c^N) - \epsilon_+ - \epsilon_-\big)$. To complete the proof of Lemma 2, note that the choice of $V_{\vec{\Phi}}$ in Eq. (59) gives us $p_\pm(\boldsymbol{x}) = \prod_{j=1}^N p_\pm(x_j)$, and since $p'(\boldsymbol{x}) = \prod_{j=1}^N p'(x_j)$ where $p'(x_j) = \mathrm{Tr}\{M_{x_j}\}/d$, $\epsilon_\pm$ is now the minimal error in discriminating two probability distributions $\{p_\pm(x_j)\}$ and $p'(x_j)$ over N repetitions, which goes to zero in the $N \to 0$ limit. ■

Supplementary Note 7. Proof of Lemma 3

A particular Kraus representation of the (single-probe) channel $\Lambda_{\theta,\vec{\phi}} = \Lambda_{\mathcal{M}} \circ \mathcal{V}_{\vec{\phi}} \circ \mathcal{U}_\theta$, with the quantum-classical channel $\Lambda_{\mathcal{M}}$ being defined in Eq. (17) for a given imperfect measurement $\mathcal{M}$, reads

$$\Lambda_{\theta,\vec{\phi}} \sim \{K_{x,j}(\theta,\vec{\phi}) = |x\rangle\langle j|\sqrt{M_x}V_{\vec{\phi}}U_\theta\}_{x,j}. \tag{75}$$

Note that as mentioned earlier, the set of orthogonal bra basis $\{\langle j|\}_{j=1}^d$ can be chosen arbitrarily, corresponding to different choice of quantum-classical channel Λ. Then, as elaborated in Methods in the main text, the asymptotic CE bound $F_N^{(\mathrm{CE,as})}$ is defined when there exists some other Kraus representation $\{\tilde{K}_{x,j}(\theta,\vec{\phi})\}$ of $\Lambda_{\theta,\vec{\phi}}$ such that $\beta_{\tilde{K}} = 0$ for any $\vec{\phi}$. Moreover, following Refs. [13–15], it suffices to consider Kraus representations that have the following properties:

$$\tilde{K}_{x,j}(\theta,\vec{\phi}) = K_{x,j}(\theta,\vec{\phi}), \tag{76}$$

$$\begin{aligned} \dot{\tilde{K}}_{x,j}(\theta,\vec{\phi}) &= \partial_\theta \tilde{K}^\dagger_{x,j}(\theta,\vec{\phi}) \\ &= \dot{K}_{x,j}(\theta,\vec{\phi}) - \mathrm{i}\sum_{x',j'} \mathsf{g}_{x,j;x',j'} K_{x',j'}(\theta,\vec{\phi}) \end{aligned} \tag{77}$$

where $\mathsf{g}(\vec{\phi})$ is an arbitrary Herimitian matrix satisfying $\mathsf{g}_{x,j';x',j'}(\vec{\phi}) = \mathsf{g}^*_{x',j';x,j}(\vec{\phi})$, and the $\beta_{\tilde{K}} = 0$ condition is equivalent to the existence of g such that

$$\mathrm{i}\sum_{x,j}\dot{K}^\dagger_{x,j}K_{x,j} - \sum_{x,j,x',j'}\mathsf{g}_{x,j;x',j'}(\vec{\phi})K^\dagger_{x,j}K_{x',j'} = 0. \tag{78}$$

Putting in Eq. (75) into Eq. (78), the $\beta_{\tilde{K}} = 0$ condition is then given by $(U_\theta = \mathrm{e}^{\mathrm{i}h\theta})$

$$h = \sum_x V^\dagger_{\vec{\phi}}\sqrt{M_x}A_x(\vec{\phi})\sqrt{M_x}V_{\vec{\phi}}, \tag{79}$$

with $A_x(\vec{\phi}) := 2\sum_{j,j'}\mathsf{g}_{x,j;x,j'}(\vec{\phi})|j\rangle\langle j'|$, which is Eq. (22) in Lemma 3—here, without specifying explicitly which probe we are referring to. ■

Supplementary Note 8. Proof of Corollary 2

We say that a detection channel $\mathcal{P}$ acts *non-trivially* on a given subset $\tilde{I} \subseteq I$ of 'inaccessible' outcomes, if for any pair $i, i' \in \tilde{I}$ there exists at least one 'observable' outcome x such that the transition probabilities of $\mathcal{P}$ satisfy $p(x|i)p(x|i') > 0$. Moreover, if this $\tilde{I}$ contains the outcomes spanning the subspace of the encoding Hamiltonian, i.e. $h = \sum_{i,i'\in\tilde{I}}\mathsf{h}_{i,i'}|i\rangle\langle i'|$ with $\det\{\mathsf{h}\} \neq 0$, we say that $\mathcal{P}$ acts non-trivially on the encoding subspace. However, for our purposes we consider $\mathcal{P}$ that act non-trivially on *all* the outcomes in I, and refer to these as *non-trivial*.

Suppose the imperfect measurement $\mathcal{M}$ is composed of a perfect von Neumann measurement, i.e., $\Pi \sim \{\Pi_i = |i\rangle\langle i|\}_i$ with $\Pi_i\Pi_{i'} = \delta_{i,i'}\Pi_i$, followed by a noisy detection channel $\mathcal{P} \sim \{p(x|i)\}$, such that $M_x = \sum_i p(x|i)\Pi_i$. As a result, the condition (79) is equivalent to $h = \sum_{i,i'\in I}V^\dagger_{\vec{\phi}}|i\rangle\sum_x\sqrt{p(x|i)}\langle i|A_x(\vec{\phi})|i'\rangle\sqrt{p(x|i')}\langle i'|V_{\vec{\phi}}$, where the entries $\langle i|A_x(\vec{\phi})|i'\rangle = 2\mathsf{g}_{x,i;x,i'}(\vec{\phi})$ can be chosen arbitrarily (of some Hermitian matrix). Now, given that $\mathcal{P}$ is *non-trivial*, so that for any pair $i, i' \in I$ there exists x such that $\sqrt{p(x|i)p(x|i')} \neq 0$, we can define a Hermitian matrix C whose *all* entries, $\mathsf{C}_{i,i'} := 2\sum_x\sqrt{p(x|i)p(x|i')}\,\mathsf{g}_{x,i;x,i'}(\vec{\phi})$ for all $i, i' \in I$, can be freely chosen (in particular, non-zero) by varying g. Consequently, we may again rewrite Eq. (79) as $\mathsf{h} = \mathsf{V}^\dagger\mathsf{C}\mathsf{V}$,

where $\mathsf{V} := \sum_{i,i'\in I} \langle i|V_{\vec{\phi}}|i'\rangle |i\rangle\langle i'|$ is an (invertible) unitary matrix. Hence, we can always satisfy the condition (79) by choosing g such that $\mathsf{C} = \mathsf{V}\mathsf{h}\mathsf{V}^\dagger$. In summary, whenever the stochastic map $\mathcal{P}$ representing the detection noise is non-trivial, Eq. (79) can always be fulfilled, which by the virtue of the asymptotic CE bound forces the MSE to asymptotically follow the SS. ∎

Supplementary Note 9. Computing $F_N^{(\mathrm{CE})}$ and $F_N^{(\mathrm{CE,as})}$ by an SDP

First, in view of the symmetry in the problem, and as suggested by the expression of $\bar{F}_N^{(\mathrm{CE,as})}$, let us focus on having the local unitary settings all being the same, i.e., $\vec{\phi}_\ell = \vec{\phi}$ for all ℓ. In this case then, we have

$$F_N^{(\mathrm{CE})}(\{\vec{\phi}_\ell\}) \Rightarrow F_N^{(\mathrm{CE})}(\vec{\phi}) := 4 \min_{\tilde{K}(\theta,\vec{\phi})} \left\{ N\|\alpha_{\tilde{K}(\theta,\vec{\phi})}\| + N(N-1)\|\beta_{\tilde{K}(\theta,\vec{\phi})}\|^2 \right\}, \tag{80}$$

and

$$F_N^{(\mathrm{CE,as})}(\{\vec{\phi}_\ell\}) \Rightarrow F_N^{(\mathrm{CE,as})}(\vec{\phi}) := 4\,N \min_{\substack{\tilde{K}(\theta,\vec{\phi})\\ \beta_{\tilde{K}(\theta,\vec{\phi})}=0}} \|\alpha_{\tilde{K}(\theta,\vec{\phi})}\|. \tag{81}$$

Then, note that the calculations for $F_N^{(\mathrm{CE})}(\vec{\phi})$ or $F_N^{(\mathrm{CE,as})}(\vec{\phi})$ can be made simpler using the fact that we are looking at estimation precision locally around some underlying true value of θ, say θ_0. Consequently, instead of considering the most general unitaries u with arbitrary θ dependence, such that $\tilde{K}_{x,j}(\theta,\vec{\phi}) = \sum_{x',j'} \mathsf{u}_{x,j;x',j'}(\theta,\vec{\phi}) K_{x',j'}(\theta,\vec{\phi})$, it suffices to consider all the Kraus representations $\tilde{K}(\theta,\vec{\phi})$ that differ from the canonical one $K(\theta,\vec{\phi})$ only by their first derivatives with respect to θ. That is, we only need to consider unitaries $\mathsf{u} = \mathrm{e}^{\mathrm{i}(\theta-\theta_0)\mathsf{g}}$ for some Hermitian generator g, such that at $\theta = \theta_0$ eventually, the Kraus operators obey Eqs (76 and 77). As result we can replace abstract minimization $\min_{\tilde{K}}$ in Eqs. (80) and (81) by $\min_{\mathsf{g}}$, i.e. minimization over all Hermitian matrices g of dimension $d|X| \times d|X|$.

The bounds $F_N^{(\mathrm{CE})}(\vec{\phi})$ and $F_N^{(\mathrm{CE,as})}(\vec{\phi})$ involve calculations of operator norms $\|\alpha_{\tilde{K}}\|$ and $\|\beta_{\tilde{K}}\|$, which can be cast as a SDP problem. We refer the readers again to Refs. ([14, 15]) for its complete derivation, and for here we shall just outline the algorithm and result. In essence, upon defining $\lambda_a^2 := \|\alpha_{\tilde{K}}\|$ and $\lambda_b^2 := \|\beta_{\tilde{K}}\|^2$, and stacking up all the Kraus operators into a vector of matrices, such that Eq. (76) now reads $\tilde{\boldsymbol{K}} = \boldsymbol{K} := [K_{x=0,j=1}(\theta,\vec{\phi}), K_{x=1,j=1}(\theta,\vec{\phi}), \cdots]^T$ and $\dot{\tilde{\boldsymbol{K}}} = \dot{\boldsymbol{K}} - \mathrm{i}\mathsf{g}\boldsymbol{K}$, we can rewrite Eq. (80) as

$$F_N^{(\mathrm{CE})}(\vec{\phi}) = 4N \min_{\mathsf{g}} \{\lambda_a^2 + (N-1)\lambda_b^2\} \quad \text{with} \quad \boldsymbol{A}, \boldsymbol{B} \geq 0, \tag{82}$$

where

$$\boldsymbol{A} = \begin{bmatrix} \sqrt{\lambda_a}\mathbb{1}_d & \dot{\tilde{\boldsymbol{K}}}^\dagger \\ \dot{\tilde{\boldsymbol{K}}} & \sqrt{\lambda_a}\mathbb{1}_{d_{\mathrm{out}}} \end{bmatrix}, \quad \boldsymbol{B} = \begin{bmatrix} \sqrt{\lambda_b}\mathbb{1}_d & (\mathrm{i}\dot{\tilde{\boldsymbol{K}}}^\dagger \boldsymbol{K})^\dagger \\ \mathrm{i}\dot{\tilde{\boldsymbol{K}}}^\dagger \boldsymbol{K} & \sqrt{\lambda_b}\mathbb{1}_{d_{\mathrm{out}}} \end{bmatrix}, \tag{83}$$

and $d_{\mathrm{out}} = d(|X|^2+1)$. In order to evaluate $F_N^{(\mathrm{CE,as})}(\vec{\phi})$ of Eq. (81), an additional constraint should just be added to Eq. (82), i.e. $\mathrm{i}\dot{\boldsymbol{K}}^\dagger \boldsymbol{K} = \boldsymbol{K}^\dagger \mathsf{g} \boldsymbol{K}$ that is simply equivalent to the condition $\beta_{\tilde{K}} = 0$ imposed in Eq. (81).

For particular simple examples analytical answers can be obtained. In particular, for the case of N qubits each sensing the phase θ in the encoding $\mathcal{U}_\theta \sim \{\mathrm{e}^{\mathrm{i}\theta\sigma_z/2}\}$, and are subject to measurement noise corresponding to binary asymmetric channel $\mathcal{P}$ mixing each binary outcome of measuring $\{\Pi_i = |\Pi_i\rangle\langle\Pi_i|\}$ with $|\Pi_{1(2)}\rangle = |\pm\rangle$, starting from the canonical Kraus representation, we find that by selecting $\mathsf{g} = r(\sigma_z \oplus \sigma_z)$ in Eq. (77) with

$$r = \frac{\delta - \sqrt{\mathsf{p}(1-\mathsf{q})} + \sqrt{\mathsf{q}(1-\mathsf{p})}}{2\delta}, \tag{84}$$

$F_N^{(\mathrm{CE,as})}(\vec{\phi})$ is equal to

$$N\left(\frac{\sqrt{\mathsf{p}(1-\mathsf{p})} - \sqrt{\mathsf{q}(1-\mathsf{q})}}{\mathsf{p}-\mathsf{q}}\right)^2. \tag{85}$$

Interestingly this result is independent of the choice of $\vec{\phi}$ with the local control unitaries taking the form $V_{\vec{\phi}} = \mathrm{e}^{\mathrm{i}\sigma_z\phi}$, which is found to be optimal over all choices of $V_{\vec{\phi}}$, and so we have actually obtained $\bar{F}_N^{(\mathrm{CE,as})} = \max_{\vec{\phi}} F_N^{(\mathrm{CE,as})}(\vec{\phi})$ in (85), and hence, Eq. (24) in the main text.

We also take note that the bound (85) can also be obtained from a conjecture, as a consequence of the G-covariance formalism (Observation 2 in main text), applied to the case with local control unitaries. That is, upon conjecturing that the optimal local control unitaries take the form of $V_{\mathrm{opt}} = \mathrm{e}^{\mathrm{i}\sigma_z\phi}$ (which as said is supported by our numerical findings), the generalisation of Eq. (27) to N independent copies of channel leads to

$$\bar{\mathcal{F}}_N^{(\mathrm{im})} \leq \bar{\mathcal{F}}[(\Lambda \circ \mathcal{U}_\theta)^{\otimes N}], \tag{86}$$

where Λ is some conjugate-map decomposition Λ satisfying the G-covariant condition (16), e.g., the quantum-classical channel (17). As $(\Lambda \circ \mathcal{U}_\theta)^{\otimes N}$ is of the form of uncorrelated noisy encoding, then, we may apply the standard technique of CE formalism to upper bound $\bar{\mathcal{F}}[(\Lambda \circ \mathcal{U}_\theta)^{\otimes N}]$ [15], which, in this case, turns out to be given exactly by (85).

Supplementary Note 10. Error-propagation formula with imperfect measurement

The mean squared error of estimators obtained from measuring the mean of some observable $\hat{O}$ with large

number of repetitions ν, is well approximated by the so-called "error-propagation formula"[16]

$$\nu\Delta^2\tilde{\theta}_N = \frac{\Delta^2\hat{O}}{\left|\frac{\partial\langle\hat{O}\rangle}{\partial\theta}\right|^2}, \tag{87}$$

where $\Delta^2 O = \langle\hat{O}^2\rangle - \langle\hat{O}\rangle^2$, with $\langle A\rangle$ being the expectation value of the operator A over the quantum state $\rho(\theta)$, i.e. $\langle A\rangle = \mathrm{Tr}\{\rho(\theta)A\}$.

For our quantum-classical channel scenario with noisy detection channel represented by the stochastic map $\mathcal{P} \sim \{p(x|i)\}$, while we have the freedom to choose the measurement basis $\Pi_{i,\vec{\phi}}$, we need to keep in mind that the only observable and effective measurement that we have is $\{M_{x,\vec{\phi}} = \sum_i p(x|i)\Pi_{i,\vec{\phi}}\}$, and it is not projective in general. The observable that we measure is thus $\hat{O} = \sum_x f_x M_{x,\vec{\phi}}$ for some $\{f_x\}$ defining the observable (which can be chosen quite arbitrarily). For N independent quantum-classical channel, we can then construct the joint observable $\hat{O} = \sum_{j=1}^N \hat{O}^{(j)} = \sum_{j=1}^N \sum_x f_x M_{x,\vec{\phi}}^{(j)}$, where j labels the different channels. Alternatively, we may also consider a second kind of joint observable, where instead of summing over the constituent single-particle operators, we perform *product* over them: $\hat{O} = \prod_{j=1}^N \hat{O}^{(j)} = \prod_{j=1}^N \left(\sum_x f_x M_{x,\vec{\phi}}\right)^{(j)}$.

While it maybe tempting, we cannot however simply use $\hat{O}^2 = \left(\sum_{j=1}^N \hat{O}^{(j)}\right)^2$ or $\hat{O}^2 = \left(\prod_{j=1}^N \hat{O}^{(j)}\right)^2$ to compute $\Delta^2\hat{O}$ in Eq. (87). The reason is, Eq. (87) uses the implicit assumption that the observable $\hat{O}$ is measured at its eigenbasis, and that is not the case here. The effective $\hat{O}^2$ that we should use in Eq. (87) is one which mimics the statistics that we would get as if we are measuring the eigenbasis, i.e., *as if* $\{M_{x,\vec{\phi}}\}$ are projective. That is, we have, for the first kind of observables,

$$\begin{aligned}\hat{O}^2 =& \sum_j \Big(\sum_x f_x M_{x,\vec{\phi}}^{(j)}\Big)^2 \\ &+ \sum_{j\neq k}\Big(\sum_x f_x M_{x,\vec{\phi}}^{(j)}\Big)\Big(\sum_x f_x M_{x,\vec{\phi}}^{(k)}\Big) \end{aligned}\tag{88}$$

$$\begin{aligned}\longrightarrow \hat{O}'^2 =& \sum_j\sum_x f_x^2 M_{x,\vec{\phi}}^{(j)} \\ &+ \sum_{j\neq k}\Big(\sum_x f_x M_{x,\vec{\phi}}^{(j)}\Big)\Big(\sum_x f_x M_{x,\vec{\phi}}^{(k)}\Big), \end{aligned}\tag{89}$$

and, for the second kind of observables,

$$\begin{aligned}\hat{O}^2 =& \prod_j \Big(\sum_x f_x M_{x,\vec{\phi}}^{(j)}\Big)^2 \\ \longrightarrow \hat{O}'^2 =& \prod_j \Big(\sum_x f_x^2 M_{x,\vec{\phi}}\Big)^{(j)} \end{aligned}\tag{90}$$

and then

$$\nu\Delta^2\tilde{\theta}_N = \frac{\langle\hat{O}'^2\rangle - \langle\hat{O}\rangle^2}{\left|\frac{\partial\langle\hat{O}\rangle}{\partial\theta}\right|^2} \tag{91}$$

for our quantum-classical channel.

We apply Eq. (91) to the case of N qubits, each of which undergoes a projective measurement $\Pi_{1,\vec{\phi}} = |+\rangle\langle+|, \Pi_{2,\vec{\phi}} = |-\rangle\langle-|$ where $\sigma_{\mathrm{x}}|\pm\rangle = \pm|\pm\rangle$, and $|X| = 2$. We consider a binary mixing channel $\mathcal{P}$ that flips the measurement outcomes with $p(1|1) = \mathsf{p}$, $p(2|2) = \mathsf{q}$, so that the effective measurements corresponding to the observed outcomes read $M_{1,\vec{\phi}} = (1+\delta)\mathbb{1}/2 + \eta\sigma_{\mathrm{x}}/2$ and $M_{2,\vec{\phi}} = (1-\delta)\mathbb{1}/2 - \eta\sigma_{\mathrm{x}}/2$ with $\eta := \mathsf{p}+\mathsf{q}-1, \delta := \mathsf{p}-\mathsf{q}$. Then, for the first kind of observables, we have

$$\hat{O} = (f_1 - f_2)\eta\hat{J}_{\mathrm{x}} + \frac{N}{2}[f_1 + f_2 + (f_1 - f_2)\delta], \tag{92}$$

$$\begin{aligned}\hat{O}'^2 =& (f_1^2 - f_2^2)\eta\hat{J}_{\mathrm{x}} + \frac{N}{2}[f_1^2 + f_2^2 + (f_1^2 - f_2^2)\delta] \\ &+ (\hat{J}_{\mathrm{x}}^2 - \frac{N}{4})\eta^2(f_1 - f_2)^2 \\ &+ (N-1)[f_1 + f_2 + (f_1 - f_2)\delta] \\ &\Big(\eta(f_1 - f_2)\hat{J}_{\mathrm{x}} + \frac{N}{4}[f_1 + f_2 + (f_1 - f_2)\delta]\Big), \end{aligned}\tag{93}$$

where $\hat{J}_\ell = \sum_{j=1}^N \frac{\sigma_\ell^{(j)}}{2}$ is the usual total angular momentum operator in the ℓ-direction with $\ell = \{\mathrm{x},\mathrm{y},\mathrm{z}\}$. After some straightforward algebra, one obtains

$$\nu\Delta^2\tilde{\theta}_N = \frac{\Delta^2\hat{J}_{\mathrm{x}}}{|\partial_\theta\langle\hat{J}_{\mathrm{x}}\rangle|^2} - \frac{\delta\langle\hat{J}_{\mathrm{x}}\rangle}{\eta|\partial_\theta\langle\hat{J}_{\mathrm{x}}\rangle|^2} + \frac{N}{4\eta^2}\frac{1-\eta^2-\delta^2}{|\partial_\theta\langle\hat{J}_{\mathrm{x}}\rangle|^2}. \tag{94}$$

Given $U_\theta = \mathrm{e}^{\mathrm{i}\theta\sigma_{\mathrm{z}}/2}$ to be the unitary encoding the estimated parameter θ onto each probe, the state of all the probes just before the measurement reads $\rho^N(\theta) := \mathrm{e}^{\mathrm{i}\theta\hat{J}_{\mathrm{z}}}\rho^N\mathrm{e}^{-\mathrm{i}\theta\hat{J}_{\mathrm{z}}}$, where ρ^N is the input N-qubit probe state. In such as case, we have that $\partial_\theta\langle\hat{J}_{\mathrm{x}}\rangle = -\langle\sin\theta\hat{J}_{\mathrm{x}} + \cos\theta\hat{J}_{\mathrm{y}}\rangle_{\rho^N} = \mathrm{Tr}\{\rho^N(\sin\theta\hat{J}_{\mathrm{x}} + \cos\theta\hat{J}_{\mathrm{y}})\}$.

For the second kind of observables, let us consider for example the (imperfect) parity operator, i.e., with $f_1 = -f_2 = 1$. Then, we have

$$\hat{O} = \hat{P} \equiv \prod_{j=1}^N \left(M_{1,\vec{\phi}} - M_{2,\vec{\phi}}\right)^{(j)} = \prod_j \left(\delta\mathbb{1} + \eta\sigma_{\mathrm{x}}\right)^{(j)}, \tag{95}$$

$$\hat{O}'^2 = \prod_{j=1}^N \left(M_{1,\vec{\phi}} + M_{2,\vec{\phi}}\right)^{(j)} = \mathbb{1}, \tag{96}$$

and finally

$$\nu\Delta^2\tilde{\theta}_N = \frac{1-\langle\hat{P}\rangle^2}{|\partial_\theta\langle\hat{P}\rangle|^2}. \tag{97}$$

Consider the measurement of the (imperfect) parity operator, $\hat{P}=\prod_{j=1}^{N}\left(M_{1,\vec{\phi}}^{(j)}-M_{2,\vec{\phi}}^{(j)}\right)$, with $M_{1,\vec{\phi}}=(1+\delta)\mathbb{1}/2+\eta\sigma_{\mathrm{x}}/2$ and $M_{2,\vec{\phi}}=(1-\delta)\mathbb{1}/2-\eta\sigma_{\mathrm{x}}/2$ as above. Then, by the error-propagation formula once more (see Supplement for details), we have

$$\nu\Delta^2\tilde{\theta}_N=\frac{1-\langle\hat{P}\rangle^2}{|\partial_\theta\langle\hat{P}\rangle|^2}, \tag{98}$$

where $\langle\hat{P}\rangle=\mathrm{Tr}\{\mathrm{e}^{\mathrm{i}\theta\hat{J}_z}\rho^N\mathrm{e}^{-\mathrm{i}\theta\hat{J}_z}\hat{P}\}$. Using the (rotated) GHZ input state, $\rho^N=|\psi\rangle\langle\psi|$, $|\psi\rangle=\mathrm{e}^{\mathrm{i}\phi\hat{J}_z}\frac{1}{\sqrt{2}}\left(|0\ldots0\rangle+|1\ldots1\rangle\right)$, we thus get

$$\nu\Delta^2\tilde{\theta}_N=\frac{1-\left(\delta^N+\eta^N\cos(N\varphi)\right)^2}{N^2\eta^{2N}\sin^2(N\varphi)}, \tag{99}$$

with $\varphi=\phi+\theta$ as before. While the parity measurement with GHZ state will perform poorly for large N by virtue of the exponential factor η^{-2N}, it does however make a good candidate for small N regime where the $1/N^2$ factor dominates. Indeed, upon optimising over ϕ, we obtain the blue curve in Fig. 6 of the main text.

Supplementary Note 11. Hierarchy of moment-based lower bounds on the FI

Using only partial information from a full probability distribution, such as considering only up to certain finite moments, one obtains a lower bound for the FI. For the case of univariate and single-parameter estimation, we provide here a simple "physicist's" reformulation for constructing such a lower bound, which consistently agrees with more abstract considerations [9–11].

We first rewrite the FI $F=\sum_x\dot{q}_{\vec{\phi},\theta}(x)^2/q_{\theta,\vec{\phi}}(x)$ by making use of essentially a simple identity: any real quadratic function $g(y)=-ay^2+2by$ with $a>0,b\in\mathbb{R}$, has its maximum given by $\max_y g(y)=g(b/a)=b^2/a$, so equivalently $F=\sum_x\max_{y_x}\{-q_{\theta,\vec{\phi}}(x)y_x^2+2\dot{q}_{\vec{\phi},\theta}(x)y_x\}$. Using a series *ansatz* $y_x=\sum_{k=0}^{K}\alpha_k w(x)^k$ for some chosen function $w(x)$ with K smaller than the cardinality of the probability distribution, we obtain a lower bound $F^{(K)}$ on FI after maximizing now over the finite set $\{\alpha_k\}_{k=0}^{K}$. By construction, we have $F^{(0)}\leq F^{(1)}\leq F^{(2)}\leq\cdots\leq F^{(K)}\leq F$, and $F^{(K)}$ can be computed straightforwardly as

$$\begin{aligned}F^{(K)}=&-\max_{\{\alpha\}}\left[\sum_{k=0}^{K}\alpha_k\sum_{j=0}^{K}\alpha_j\sum_x q_{\theta,\vec{\phi}}(x)w(x)^{k+j}\right.\\&\left.+\;2\sum_{k=0}^{K}\alpha_k\sum_x\dot{q}_{\vec{\phi},\theta}(x)w(x)^k\right]\\=&\max_{\boldsymbol{\alpha}}-\boldsymbol{\alpha}^TA\boldsymbol{\alpha}+2\boldsymbol{b}^T\boldsymbol{\alpha}=\boldsymbol{b}^TA^{-1}\boldsymbol{b},\end{aligned} \tag{100}$$

where $\boldsymbol{\alpha}=\left(\alpha_0,\alpha_1,\cdots,\alpha_K\right)^T$, and A and $\boldsymbol{b}$ are as in Eqs. (26, 27) in the main text, with the more general replacement $\mathbb{E}[x^j]\to\mathbb{E}[w(x)^j]$ and $\dot{\mathbb{E}}[x^j]\to\dot{\mathbb{E}}[w(x)^j]$. If we choose further $w(x)=x$, $F^{(K)}$ becomes a lower bound on F that takes into account up to the $2K$-th moment of the distribution $q_{\theta,\vec{\phi}}$. For $K=0$ and $K=1$, we have explicitly $F^{(0)}=0$ and $F^{(1)}=\dot{\mathbb{E}}[w(x)]^2/(\mathbb{E}[w(x)^2]-\mathbb{E}[w(x)]^2)$. For $K\geq2$, Eq. (100) can be computed numerically and efficiently by standard matrix inversion techniques.

SUPPLEMENTARY REFERENCES

* y.len@cent.uw.edu.pl
† tgefen@caltech.edu
‡ jan.kolodynski@cent.uw.edu.pl

[1] Note that, in the main text, the quantum-classical channel $\Lambda_{\theta,\vec{\phi}}$ is defined together with the encoding $\mathcal{E}_\theta$ as well as the control operation, i.e. $\Lambda_{\theta,\vec{\phi}}=\Lambda\circ\mathcal{V}_{\vec{\phi}}\circ\mathcal{E}_\theta$.

[2] Braunstein, S. L., & Caves, C. Statistical distance and the geometry of quantum states. *Phys. Rev. Lett.* **72**, 3439 (1994).

[3] Escher, B. M. Quantum noise-to-sensibility ratio. Preprint at https://arxiv.org/abs/1212.2533 (2012).

[4] Macieszczak, K. Quantum Fisher information: variational principle and simple iterative algorithm for its efficient computation. Preprint at https://arxiv.org/abs/1312.1356 (2013).

[5] Bengtsson, I, & Życzkowski, K. *Geometry of quantum states: An introduction to quantum entanglement*, (Cambridge University Press, 2006).

[6] Holevo, A. S. A note on covariant dynamical semigroups. *Rep. Math. Phys.* **32**, 211 (1993).

[7] Holevo, A. S. Covariant quantum markovian evolutions. *J. Math. Phys.* **37**, 1812 (1996).

[8] Smirne, A., Kołodyński, J., Huelga, S. F., & Demkowicz-Dobrzański, R. Ultimate precision limits for noisy frequency estimation. *Phys. Rev. Lett.* **116**, 120801 (2016).

[9] Sankaran, M. On an analogue of bhattacharya bound. *Biometrika* **51**, 268 (1964).

[10] Jarrett, R. G. Bounds and expansions for fisher information when the moments are known. *Biometrika* **71**, 101–113 (1984).

[11] Stein, M. S. Sensitivity analysis for binary sampling systems via quantitative fisher information lower bounds. Preprint at https://arxiv.org/abs/1512.03473 (2021).

[12] Basu, A., Shioya, H. & Park, C. *Statistical inference: the minimum distance approach* (Chapman and Hall/CRC, 2019).

[13] Fujiwara, A. & Imai, H. A fibre bundle over manifolds of quantum channels and its application to quantum statistics. *J. Phys. A: Math. Theor.* **41**, 255304 (2008).

[14] Demkowicz-Dobrzański, R., Kołodyński, J. & Guţă, M. The elusive Heisenberg limit in quantum-enhanced metrology. *Nat. Commun.* **3**, 1063 (2012).

[15] Kołodyński, J. & Demkowicz-Dobrzański, R. Efficient tools for quantum metrology with uncorrelated noise. *New J. Phys.* **15**, 073043 (2013).

[16] Wineland, D. J., Bollinger, J. J., Itano, W. M., Moore, F. L. & Heinzen, D. J. Spin squeezing and reduced quantum noise in spectroscopy. *Phys. Rev. A* **46**, R6797–

R6800 (1992).
